# Development of NanoLuc-targeting protein degraders and a universal reporter system to benchmark tag-targeted degradation platforms

**DOI:** 10.1038/s41467-022-29670-1

**Published:** 2022-04-19

**Authors:** Christoph Grohmann, Charlene M. Magtoto, Joel R. Walker, Ngee Kiat Chua, Anna Gabrielyan, Mary Hall, Simon A. Cobbold, Stephen Mieruszynski, Martin Brzozowski, Daniel S. Simpson, Hao Dong, Bridget Dorizzi, Annette V. Jacobsen, Emma Morrish, Natasha Silke, James M. Murphy, Joan K. Heath, Andrea Testa, Chiara Maniaci, Alessio Ciulli, Guillaume Lessene, John Silke, Rebecca Feltham

**Affiliations:** 1 The Walter and Eliza Hall Institute for Medical Research, 1G Royal Parade, Parkville, Melbourne, VIC 3052 Australia; 2 Department of Medical Biology, University of Melbourne, Parkville, VIC 3052 Australia; 3 Promega Biosciences LLC, 277 Granada Drive, San Luis Obispo, CA 93401 USA; 4 Amphista Therapeutics Ltd, Bo’Ness Road Newhouse, Glasgow, ML1 5UH UK; 5 Chemistry School of Natural and Environmental Sciences, Bedson Building, Newcastle University Edwards Walk, Newcastle, NE1 8QB UK; 6 Division of Biological Chemistry and Drug Discovery, School of Life Sciences, University of Dundee, Dow Street, Dundee, DD1 5EH UK; 7 Department of Pharmacology and Therapeutics, University of Melbourne, Parkville, VIC 3052 Australia

**Keywords:** Molecular engineering, Target validation, Chemical modification, Proteolysis

## Abstract

Modulation of protein abundance using tag-Targeted Protein Degrader (tTPD) systems targeting FKBP12^F36V^ (dTAGs) or HaloTag7 (HaloPROTACs) are powerful approaches for preclinical target validation. Interchanging tags and tag-targeting degraders is important to achieve efficient substrate degradation, yet limited degrader/tag pairs are available and side-by-side comparisons have not been performed. To expand the tTPD repertoire we developed catalytic NanoLuc-targeting PROTACs (NanoTACs) to hijack the CRL4^CRBN^ complex and degrade NanoLuc tagged substrates, enabling rapid luminescence-based degradation screening. To benchmark NanoTACs against existing tTPD systems we use an interchangeable reporter system to comparatively test optimal degrader/tag pairs. Overall, we find the dTAG system exhibits superior degradation. To align tag-induced degradation with physiology we demonstrate that NanoTACs limit MLKL-driven necroptosis. In this work we extend the tTPD platform to include NanoTACs adding flexibility to tTPD studies, and benchmark each tTPD system to highlight the importance of comparing each system against each substrate.

## Abstract (web summary)

tag-Targeted Protein Degrader (tTPD) systems are powerful tools for preclinical target validation. Here the authors extend the tTPD platform by developing NanoTACs that degrade NanoLuc tagged substrates and benchmark each tTPD system using an interchangeable tag reporter system.

## Introduction

Proteolysis targeting chimeras (PROTACs)/degraders are heterobifunctional molecules that trigger ubiquitination of cellular targets by hijacking E3 ubiquitin ligases resulting in rapid proteasomal degradation^1^. Powerful genetic tools exist to experimentally modulate the abundance of gene products, such as CRISPR-Cas9 genome editing and RNA interference^2^. However, genetic technologies are limited in their ability to assess acute changes in protein levels, and present additional challenges as protein depletion may take days depending on protein half-life and protein loss is typically irreversible, which may in turn impart a selection pressure for compensatory mechanisms. Small-molecule perturbations on the other hand are popular as they can be used to rapidly, and for the most part, reversibly assess biological responses^3^. Yet, small-molecule inhibitors can be limited by off-target effects, the disruption of discrete domain function, occupancy-driven modes of action and the cost and time associated with their development.

Degrader-mediated degradation of proteins presents exciting opportunities to explore the not-yet-drugged proteome as the majority of human proteins, such as transcription factors, non-enzymatic proteins and scaffolding proteins lack active sites rendering them difficult to inhibit with small-molecule inhibitors. Degraders consist of a target-binding ligand and an E3-ligase-binding ligand separated by a chemical linker. The design and optimisation of each of these elements can be critical to achieve efficient substrate degradation as the ligand and the linker can form favourable inter- and intramolecular interactions within the E3-degrader-substrate ternary complex^4^. For example, varying the linker length can enhance the degradation of substrates by reducing steric clashes that can occur between the E3 and substrate^5,6^.

**Fig. 1:**
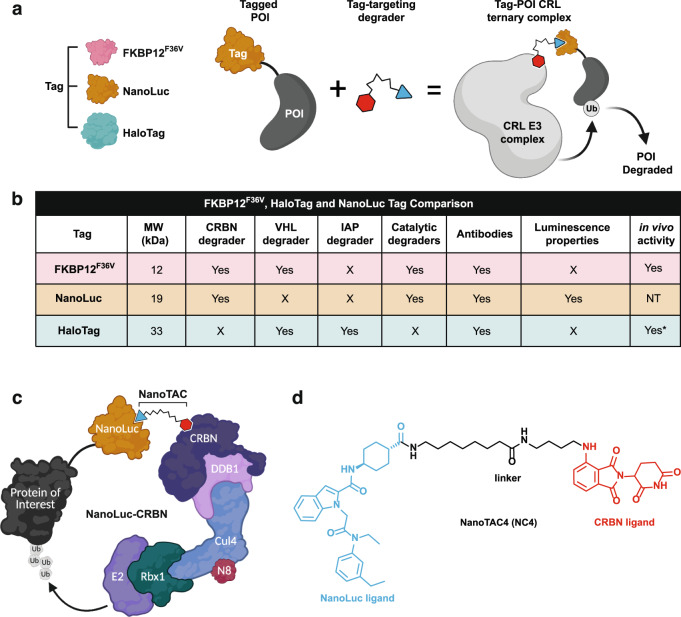
Development of NanoTACs; a NanoLuc-targeting degrader system. **a** Schematic depicting the tTPD systems. FKBP12^F36V^, NanoLuc or Halo epitope tags are fused to proteins of interest (POI) and tag-targeting heterobifunctional degrader compounds are employed to hijack cullin-RING ligase (CRL) complexes to trigger proteasomal degradation of the tagged POI. **b** Comparison of tools and properties of each tag for tag-targeted protein degradation. NT not tested. Asterisk (*) see discussion for further information. **c** Schematic depicting the NanoLuc-CRBN tTPD system. **d** Chemical structure of the heterobifunctional NanoLuc-targeting PROTAC; NanoTAC4 (NC4).

Tag-Targeted Protein Degrader (tTPD) systems direct degrader compounds to protein tags using tag-targeting heterobifunctional molecules (Fig. 1a)^7^. tTPD systems are remarkable technologies that permit rapid and reversible degradation of tagged substrate proteins through cell-permeable and in vivo compatible degraders^8–11^. tTPD systems include the monovalent molecular glue-based auxin-inducible degron (AID)^12^, ligand-inducible affinity-directed protein missile (L-AdPROM)^13^, CH6-tag directed specific and nongenetic inhibitors of apoptosis protein [IAP]-dependent protein erasers (SNIPERs)^14^, BromoTag^15^, FK506-binding protein 12 (FKBP12) and HaloTag targeting Cereblon (CRBN)- or Von Hippel-Lindau (VHL)-based systems. Degrader molecules that target FKBP12^WT^ or FKBP12^F36V^ (hereafter referred to as FKBP^F36V^) and recruit the cullin-RING ligase (CRL) complexes, CRL4^CRBN^ and CRL2^VHL^ to an FKBP-tagged substrate have the prefix dTAG (dTAG13, dTAG48, dTAG^V^-1)^9,10,16,17^. HaloTag targeting heterobifunctional degraders have been referred to as HaloPROTACs^18,19^, which utilise the CRL2^VHL^ system, or HaloTag-binding degradation inducers^20^, which utilise the IAP proteins.

For simplicity, we refer to all tag-targeting heterobifunctional degrader systems listed above as tTPD systems and have abbreviated each degrader name and the targeted tag/E3s used in this study (Table 1). We have also abbreviated all degrader names to reflect the tag and cullin substrate receptor they bind, e.g. NanoLuc-CRBN = NC (Table 1).

**Table 1:** tTPD system nomenclature.

Abb.	Tag/E3 Abb.	Tag-targeting degrader	Target (Tag)	E3-ligase complex
FC1	F36V-CRBN	dTAG13	FKBP12^F36V^	CUL4^CRBN^
FC2	F36V-CRBN	dTAG48	FKBP12^F36V^	CUL4^CRBN^
FV1	F36V-VHL	dTAG^V^-1	FKBP12^F36V^	CUL2^VHL^
NC1	NanoLuc-CRBN	NanoTAC1	NanoLuc	CUL4^CRBN^
NC2	NanoLuc-CRBN	NanoTAC2	NanoLuc	CUL4^CRBN^
NC3	NanoLuc-CRBN	NanoTAC3	NanoLuc	CUL4^CRBN^
NC4	NanoLuc-CRBN	NanoTAC4	NanoLuc	CUL4^CRBN^
NC5	NanoLuc-CRBN	NanoTAC5	NanoLuc	CUL4^CRBN^
NC*	NanoLuc-CRBN	NanoTAC*	NanoLuc	CUL4^CRBN-ME *Inactive*^
NV1	NanoLuc-VHL	NanoTAC^V^-1	NanoLuc	CUL2^VHL^
NV2	NanoLuc-VHL	NanoTAC^V^-2	NanoLuc	CUL2^VHL^
HC1	Halo-CRBN	HaloPROTAC-1	HaloTag7	CUL4^CRBN^
HC2	Halo-CRBN	HaloPROTAC-2	HaloTag7	CUL4^CRBN^
HC3	Halo-CRBN	HaloPROTAC-3	HaloTag7	CUL4^CRBN^
HV1	Halo-VHL	HaloPROTAC-A	HaloTag7	CUL2^VHL^
HV2	Halo-VHL	HaloPROTAC-E	HaloTag7	CUL2^VHL^

*Abb*. abbreviation.

FKBP12 heterobifunctional degraders initially emerged to target chimeric FKBP12^WT^-tagged proteins for degradation^17^. However, the heterobifunctional degraders dFKBP-1 and dFKBP-2 also targeted endogenous wild-type FKBP12. Loss of endogenous wild-type FKBP12 may lead to undesired biological side effects and confound experimental interpretation, so heterobifunctional degraders that targeted an engineered mutant FKBP^F36V^ were generated^9,10^. The F36V mutation creates a hole in the 12 kDa FKBP protein that allows 1000-fold selectivity over the wild-type protein by a bumped synthetic FKBP^F36V^ directed ligand^21^, and heterobifunctional tools to target FKBP^F36V^-tagged substrates for proteasomal degradation are now well established.

HaloTag-binding degradation inducers and HaloPROTACs were generated to similarly target the 33 kDa HaloTag and HaloTag7 to trigger degradation of HaloTag and HaloTag7 fusion proteins, respectively^18–20^. HaloTag is an enzyme engineered from the bacterial dehalogenase enzyme that binds covalently to an alkyl chloride moiety^22^, while HaloTag7 (hereafter referred to as Halo) is a variant of the HaloTag developed to increase the stability of the enzyme^23^.

FKBP^F36V^ degraders and HaloPROTACs trigger efficient degradation of tagged substrate proteins^9,10,18–20,24^. However, these tag-targeting systems have some drawbacks; limited tools exist to interrogate FKBP^F36V^-tagged proteins and FKBP^F36V^ has low genomic insertion efficiency^24^. Halo is a large tag relative to other protein tags and HaloPROTACs are non-catalytic in nature, i.e. they are consumed during the reaction and, therefore, cannot be recycled and reused due to the covalent binding properties, which limits their effectivity. Non-catalytic and irreversible covalent binding degraders are limited in their efficiency due to the need to achieve stoichiometric occupancy of the tagged protein to trigger complete degradation of the target protein. Degraders with non-covalent cognate binding ligands, such as FKBP^F36V^-targeting degraders are not limited in this respect as they can function catalytically at sub-stoichiometric concentrations.

NanoLuc (NLuc) is a 19 kDa luciferase enzyme that relies on the substrate furimazine to produce high-intensity glow-type bioluminescence and is one of the few commercially available luciferase enzymes^25^. In addition to its luminescence properties, NanoLuc is an attractive protein tag compared to other tags due to its stability, small size and the availability of in vivo compatible substrates^26^.

In this work, we develop and characterise catalytic NanoLuc-targeting PROTACs (NanoTACs) that hijack the CRL complexes to trigger proteasomal degradation of NanoLuc-tagged substrates to expand the tTPD repertoire. Using a universal reporter system harbouring Halo-EGFP/Firefly-NanoLuc-FKBP^F36V^ we benchmark each tTPD system in identical cellular settings. We show that all of the tTPD systems offer up the prospect of selectively and reversibly depleting proteins of interest at will. However, each of the three tags offers advantages and disadvantages (Fig. 1b), and which tag to choose really hinges on the researcher’s intentions for their tagged protein of interest.

## Results

### Development of NanoTACs; a NanoLuc-targeting degrader system

We developed heterobifunctional degraders that bind NanoLuc (NanoTACs) to trigger protein degradation of NanoLuc-tagged substrates building upon the existing tTPD systems that direct degraders to FKBP^F36V^ or Halo-tagged substrates (Fig. 1a). While each tTPD system has specific advantages, NanoLuc is the only tag that offers luminescence properties. Additionally, NanoTACs are catalytic and can act as degradation catalysts, unlike HaloPROTACs (Fig. 1b). Our most potent NanoTAC recruits the CRL4^CRBN^ E3-ligase complex to trigger the degradation of NanoLuc-tagged substrate proteins (Fig. 1c). To generate this NanoTAC (NC4) (Fig. 1d), we synthesised two series of NanoTAC analogues. NC1 and NC2 were generated by linking NanoLuc inhibitor 1, a small-molecule inhibitor of NanoLuc with a low nanomolar IC_50_ (31 nM) against the enzymatic activity of NanoLuc^27^ (Supplementary Fig. 1a), with the CRBN ligand thalidomide using varied alkyl chain linker lengths (Supplementary Fig. 1b, c). NC3, NC4, NC5 and the structurally related control analogue unable to recruit CRBN, NC*, were prepared by coupling a derivative of the more potent NanoLuc inhibitor 2, with a single-digit nanomolar IC_50_ (4.2 nM) against the enzymatic activity of NanoLuc^27^ (Supplementary Fig. 1d) with the CRBN ligand thalidomide using varied linker lengths (Supplementary Fig. 1e-g). Lastly, NV1 and NV2 were prepared by coupling NanoLuc inhibitor 2 to the widely used VHL ligand derivatives: VH298 and VH032^18,19^ (Supplementary Fig. 1h, i).

### Characterisation of NanoTACs, and selection of a potent NanoTAC: NC4

To test the degradation capacity of the first series of analogues we cloned and stably expressed through lentiviral transduction a versatile and universal tTPD reporter protein that enabled comparative studies in HEK293T cells. This reporter contains all three protein tags: Halo, FKBP^F36V^ and NanoLuc in addition to the fluorescent protein EGFP to create the artificial recombinant fusion protein Halo-EGFP-NanoLuc-FKBP^F36V^ (H-E-N-F) (Supplementary Fig. 2a). All graphs in this study have been colour-coded corresponding to the targeted tag to ease interpretation. The F36V-CRBN degrader FC1 almost completely degraded the fusion protein at a concentration of 100 nM as judged by Western blot while the NanoLuc-CRBN targeting NanoTACs NC1 and NC2 led to partial degradation of the fusion protein at 500 nM and 1 µM (Supplementary Fig. 2b).

We next tested the second series of NanoTAC analogues. To explore whether NanoTAC degraders could be improved by recruiting a different E3 ligase, we employed NV1 and NV2 that hijack the CRL2^VHL^ complex (Supplementary Fig. 2c). NV1 and NV2 were unable to trigger degradation of the H-E-N-F fusion protein over the wide range of tested concentrations up to 16 μM (Supplementary Fig. 2d, e). The lack of degradation was not due to an absence of VHL activity as the F36V-VHL targeting degrader (FV1) triggered complete degradation of the fusion protein by Western blot (Supplementary Fig. 2d, e).

**Fig. 2:**
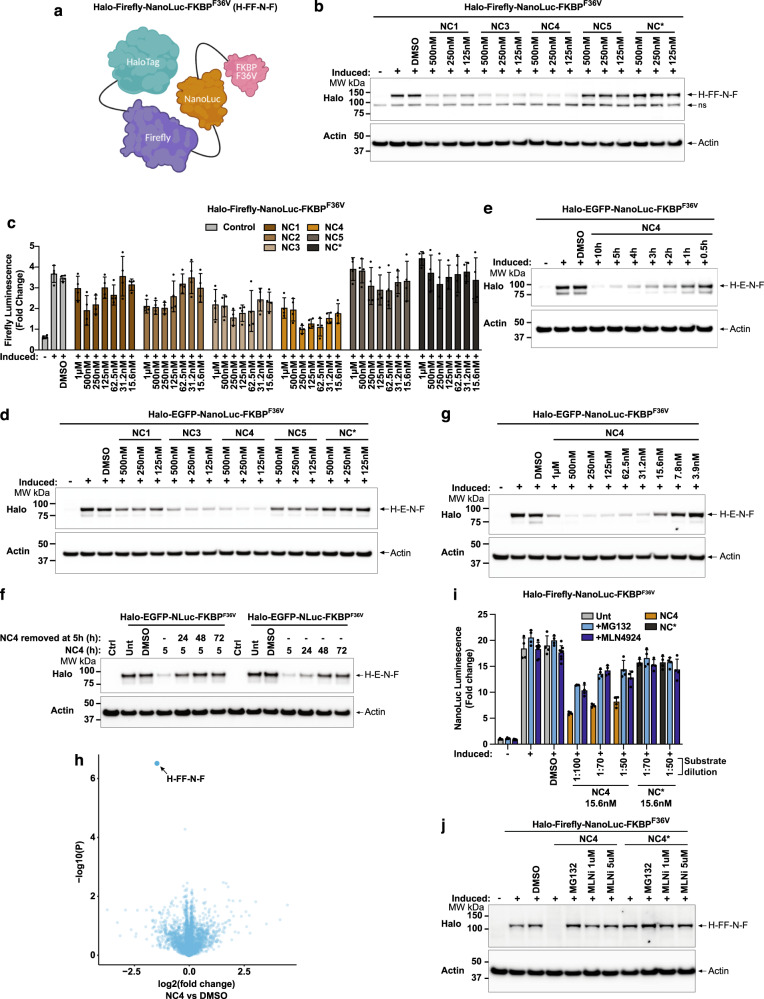
Identification of NC4 as a potent NanoTAC degrader. **a** Schematic depicting the Halo-Firefly-NanoLuc-FKBP^F36V^ (H-FF-N-F) fusion protein. **b** Western blot analysis of lysates from HEK293T cells expressing H-FF-N-F, induced with 20 ng/mL doxycycline overnight, stimulated for 5 h (ns non-specific band). **c** Firefly luminescence from cells in **b**, induced with 20 ng/mL doxycycline overnight, stimulated for 5 h. **d** Western blot analysis of lysates from HEK293T cells expressing H-E-N-F, induced with 20 ng/mL doxycycline overnight, stimulated for 5 h. **e** Western blot analysis of lysates from cells in **d**, induced with 20 ng/mL doxycycline overnight, stimulated with 125 nM of NC4 for the indicated times. **f** Western blot analysis of lysates from immortalized MDFs (iMDFs) constitutively expressing H-E-N-F or wild-type iMDFs (ctrl), cells were left untreated (Unt), stimulated with DMSO or 125 nM of NC4 for 5, 24, 48 or 72 h. NC4 was left in the culture medium for the duration of the time-course or removed from the culture medium at 5 h, and cells incubated post degrader removal for 24, 48 or 72 h. **g** Western blot analysis of lysates from cells in **d**, induced with 20 ng/mL doxycycline overnight, stimulated with NC4 for 5 h. **h** Volcano plot quantifying proteins significantly downregulated in 293 T cells expressing the H-FF-N-F reporter after treatment with DMSO or 125 nM of NC4 for 5 h. Proteins significantly downregulated (using the *q*-value) with NC4 treatment when compared to DMSO treatment are labelled and indicated with an arrow. **i** NanoLuc luminescence from cells in **b**, induced with 20 ng/mL doxycycline overnight, stimulated with 10 μM MG132 or 1 μM MLN4924 for 1 h prior to treatment with 15.6 nM NC4 or NC* for 4 h. Substrate (furimazine) was diluted 1:100, 1:70 and 1:50 prior to detecting luminescence. **j** Western blot analysis from cells in B, induced with 20 ng/mL doxycycline overnight, stimulated with 10 μM MG132, or 1 or 5 μM MLN4924 for 1 h prior to stimulation with DMSO or 125 nM of NC4 or NC* for 5 h. **c**, **i** Error bars (EB) represent mean ± SD from *N* = 4 technical repeats. All experiments were repeated independently three times and a representative Fig. is shown. Source data are provided as a Source Data file.

Luminescence is a higher throughput readout than Western blotting, however, NanoTAC degraders compete with NanoLuc substrates as they interfere with the conversion of furimazine to furimamide (Supplementary Fig. 2f). To address this, we generated a fusion protein that incorporates Firefly: Halo-Firefly-NanoLuc-FKBP^F36V^ (H-FF-N-F) (Fig. 2a). This construct allows for the direct side-by-side comparison of all tTPD systems including the NanoLuc tTPD system using a Firefly luciferase, which emits a luminescence signal that can be assayed independently from NanoLuc through the use of distinct substrates. Using the H-FF-N-F reporter protein we tested the second series of NanoLuc-CRBN targeting NanoTAC analogues NC3, NC4, NC5 and NC* (inactive control). We observed a greater reduction in Firefly luminescence, and protein levels by Western blot, upon NanoTAC treatment of cells expressing the H-FF-N-F fusion protein (Fig. 2b, c). Specifically, NC3 and NC4 showed a marked improvement in the extent of substrate degradation compared to the first series of analogues NC1 and NC2, particularly at lower concentrations (Fig. 2b, c). At higher concentrations, NC4 demonstrated the hook effect (Fig. 2c), which is a characteristic feature of heterobifunctional degraders whereby high concentrations block degradation due to ligand saturation and inability to form a ternary complex^28^. Interestingly, NC5 which harbours a linker that is just one carbon length shorter than NC4 was incapable of triggering degradation of the fusion protein, possibly due to unfavourable steric interactions as a result of the decreased linker length (Fig. 2b, c). As expected, the control compound NC* did not trigger degradation of the fusion protein (Fig. 2b, c). NanoTAC degraders were equally capable of triggering degradation of the H-E-N-F substrate protein with NC3 and NC4 consistently outperforming the first analogue NC1 (Fig. 2d). Overall, NC4 displayed the most effective degradation profile and was therefore characterised further.

NC4 exhibited approximately a 30-fold reduced inhibition of NanoLuc enzymatic activity compared to the parent inhibitor (NanoLuc inhibitor 2) indicating that conjugation of the CRBN ligand altered the binding kinetics of NC4 (Supplementary Fig. 2g). Strikingly, even though NC4 exhibited reduced inhibition of NanoLuc enzymatic activity NC4 was still capable of rapidly reducing protein levels within 2 h and degradation was sustained over a period of 10 h (Fig. 2e). Further to this, degradation by NC4 was sustained over 24 h when stimulation was maintained in the culture medium, and reversible by 24 h when stimulation was removed after 5 h of initial treatment (Fig. 2f). NC4 was also capable of triggering degradation of the fusion protein at low concentrations, ~30 nM, with a slight hook effect observed at higher concentrations, similar to that observed in Firefly luminescence-based readouts (Fig. 2c). Importantly, the global proteomic analysis demonstrated that NC4 triggers specific degradation of the target substrate with no significant off-target degradation observed when assayed against 5591 proteins (Fig. 2h). Notably, our proteomics analysis did not detect the Thalidomide-induced CRBN neosubstrates, IKZF1 or IKZF3, and therefore we cannot comment on whether these neosubstrates are altered upon NC4 treatment.

To explore whether NanoLuc luminescence could be used in conjunction with NanoTAC treatment we titrated NC4 against NC* from 2–10 h. We reasoned that the catalytic nature of NC4 would allow for substrate degradation at concentrations below that required to inhibit NanoLuc’s enzymatic luminescence activity. To this extent we compared the ability of NC4 to reduce luminescence with the control compound, NC*, which lacks the degradative capability. As predicted, we observed clear differences in NanoLuc luminescence at 31.2 nM and 15.6 nM between NC4 and NC* (Supplementary Fig. 2h). Importantly, the reduction in NanoLuc luminescence and the observed depletion of the reporter by Western blot was indicative of cullin-based proteasome-mediated degradation, as pre-treatment with the proteasome inhibitor MG132 or the NEDD8-activating enzyme inhibitor MLN4924 could rescue both the reduction in luminescence and protein levels (Fig. 2i, j). Notably, a slightly enhanced rescue was observed when the substrate furimazine concentration was increased for NanoLuc detection, consistent with the competitive nature of NanoLuc inhibition by NC4 (Fig. 2i).

### FKBP^F36V^-targeting degraders outperform other tTPD systems

**Fig. 3:**
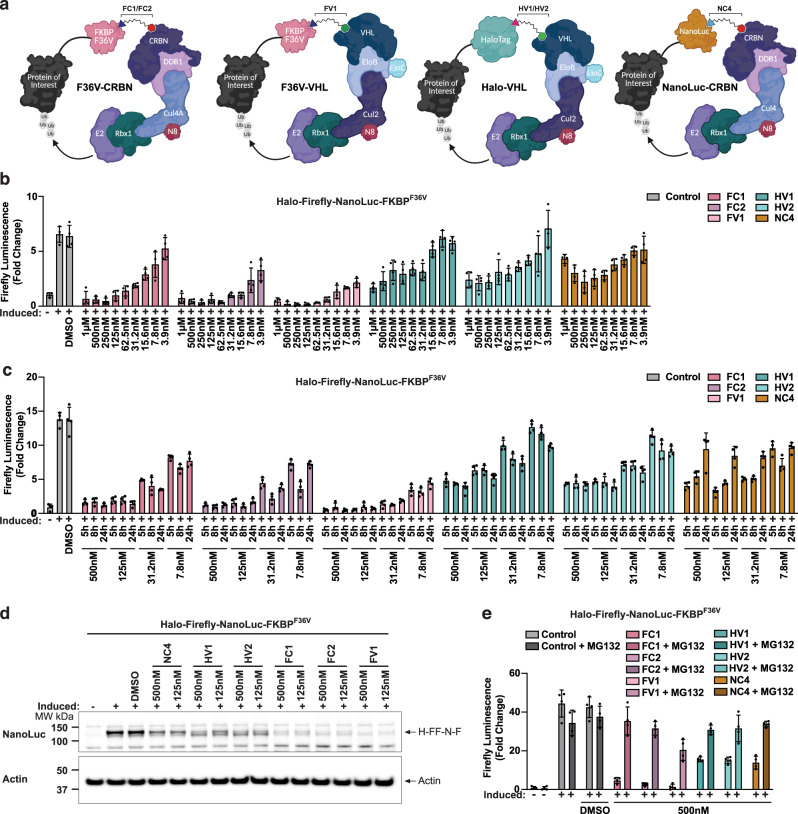
FKBP^F36V^ tTPD systems outperform other tTPD systems. **a** Schematic representation of the Halo-VHL, F36V-CRBN, F36V-VHL and NanoLuc-CRBN tag-targeting degrader systems. **b** Firefly luminescence from 293 T cells stably expressing doxycycline-inducible H-FF-N-F, treated with 20 ng/mL doxycycline overnight (induced), then stimulated with the indicated concentrations FC1 (F36V-CRBN), FC2 (F36V-CRBN), FV1 (F36V-VHL), HV1 (Halo-VHL), HV2 (Halo-VHL) or NC4 (NanoLuc-CRBN) for 5 h. **c** Firefly luminescence from cells used in **b**, treated with 20 ng/mL doxycycline overnight (induced), then stimulated with the indicated concentrations FC1, FC2, FV1, HV1, HV2 or NC4 for the indicated times. **d** Western blot analysis of total cell lysates from cells expressing H-FF-N-F, treated with 20 ng/mL doxycycline overnight (induced), then stimulated with the indicated concentrations of degraders for 5 h. **e** Firefly luminescence from cells in **b**, treated with 20 ng/mL doxycycline overnight (induced), following treatment with 20 μM MG132 for 1 h prior to stimulation with the indicated concentrations FC1, FC2, FV1, HV1, HV2 or NC4 for 5 h. **b**, **c**, **e** EB represent mean ± SD from *N* = 4 technical repeats. All experiments were repeated independently three times, and a representative Fig. is shown. Source data are provided as a Source Data file.

To benchmark existing tTPD systems against NanoTACs and assess the relative degradative performance of each system, we synthesised degraders that hijack VHL and CRBN to trigger proteasomal degradation of FKBP^F36V^ and Halo-tagged substrates^9,10,18,19^, and generated a series of Halo targeting CBRN recruiting degraders; HC1, HC2 and HC3 (Supplementary Fig. 3a). Halo-VHL degraders (HV1 and HV2) target Halo-tagged substrates^19^, while both F36V-CRBN (FC1 and FC2) degraders and the F36V-VHL (FV1) degrader target FKBP^F36V^-tagged substrates^9,10^ (Fig. 3a). Notably, similar Halo-CRBN molecules have been tested for cellular penetration using the Chloroalkane Penetration Assay (CAPA)^29^; however, degradation studies have not been performed. The Halo-CRBN molecules we generated were unable to trigger efficient substrate degradation over a wide concentration range (Supplementary Fig. 3b) possibly due to reduced cellular accumulation of these compounds or the inability to promote ternary complex formation.

**Fig. 4:**
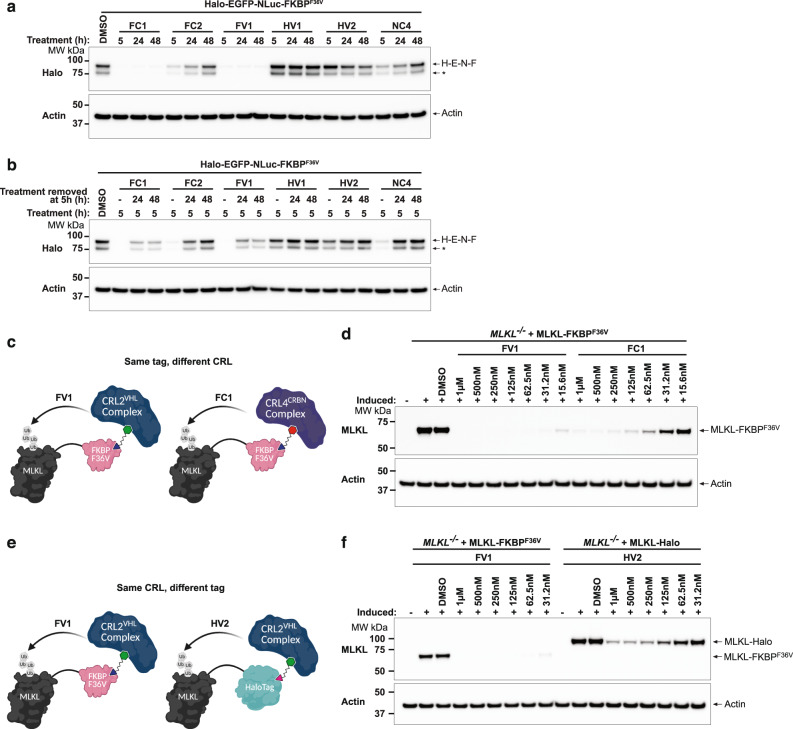
F36V-VHL degrader outperforms CRBN and Halo targeting degraders. **a** Western blot analysis of total cell lysates from immortalized MDFs (iMDFs) constitutively expressing H-E-N-F. Cells were stimulated with DMSO or stimulated with 500 nM of FC1, FC2, FV1, HV1, HV2 or NC4 degrader compounds for 5, 24 or 48 h. Asterisk (*) indicates possible cleavage product. **b** Western blot analysis of total cell lysates from immortalized MDFs (iMDFs) constitutively expressing H-E-N-F. Cells were stimulated with DMSO or stimulated with 500 nM of FC1, FC2, FV1, HV1, HV2 or NC4 degrader compounds for the first 5 h. Degraders were then removed from the culture medium at 5 h and cells were incubated for 24 or 48 h. Minus sign (−) indicates that sample was harvested at the time the treatment was removed (5 h). Asterisk (*) indicates possible cleavage product. **c** Schematic depicting the comparison of selected tTPD systems; F36V-VHL (FV1) and F36V-CRBN (FC1). **d** Western blot analysis of total cell lysates from *MLKL*^*−/−*^ HT29 cells stably expressing the doxycycline-inducible C-terminal fusion protein MLKL-FKBP^F36V^. Cells were treated with 40 ng/mL doxycycline overnight to induce the constructs, then stimulated with the indicated concentrations of FV1 (F36V-VHL) or FC1 (F36V-CRBN) for 5 h. **e** Schematic depicting the comparison of selected tTPD systems; F36V-VHL (FV1) and Halo-VHL (HV2). **f** Western blot analysis of total cell lysates from *MLKL*^*−/−*^ HT29 cells stably expressing the doxycycline-inducible C-terminal fusion protein MLKL-FKBP^F36V^ or MLKL-Halo treated with 40 ng/mL doxycycline overnight to induce the construct, then stimulated with the indicated concentrations of FV1 (F36V-VHL) or HV2 (Halo-VHL). All experiments were repeated independently three times, and a representative Fig. is shown. Source data are provided as a Source Data file.

To directly compare the NanoTAC system to the FKBP^F36V^ and Halo systems (Fig. 3a) we employed cells expressing either the H-FF-N-F or the H-E-N-F fusion proteins (Fig. 2a and Supplementary Fig. 2a). Using Firefly luminescence, we were able to directly compare the degradative capacity of the F36V-CRBN, F36V-VHL, Halo-VHL and NanoLuc-CRBN systems against the same substrate. Overall, F36V-CRBN degraders (FC1 and FC2) outperformed the Halo-VHL degraders (HV1 and HV2), while the F36V-VHL degrader (FV1) triggered the most efficient substrate depletion over a wide concentration range after 5 h (Fig. 3b). A similar degradation profile was observed from the corresponding NanoLuc luminescence detected from the H-FF-N-F and H-E-N-F substrates in the same assay (Supplementary Fig. 3c, d). Halo-VHL degraders, although less efficient at triggering substrate depletion compared to the F36V-CRBN and F36V-VHL degraders, particularly at lower concentrations, displayed slightly improved substrate degradation over 8 and 24 h at relatively low concentrations (Fig. 3c, Supplementary Fig. 3e). The NanoLuc-CRBN targeting NanoTAC, NC4, was capable of triggering degradation of the fusion protein over a wide concentration range and to levels comparable to that of the Halo and FKBP^F36V^ tTPD systems displaying a calculated D_max_ of 77% and a DC_50_ of 21.6 nM against our fusion (Fig. 3b, c). NC4, displayed a similar degradation profile to that of the HaloPROTACs (Fig. 3b), but reproducibly appeared to lose efficacy after 24 h (Fig. 3c). Consistent with our luminescence results, we observed reduced levels of the fusion proteins with all tested tTPD degrader systems by Western blot (Fig. 3d, Supplementary Fig. 3f), and importantly the observed reduction in luminescence could be rescued by co-treatment with the proteasome inhibitor MG132 (Fig. 3e, Supplementary Fig. 3g). Next we compared the durability of degradation across all tTPD systems by performing wash-out recovery experiments. We transitioned to a constitutive system where the H-E-N-F protein was expressed under the control of a ubiquitin promoter to avoid foreseeable complications with wash-out of the doxycycline in our inducible systems. Interestingly FC1 and FV1 maintained near complete degradation up to 48 h (Fig. 4a), and were capable of maintaining clear degradation for up to 48 h when the degrader was removed from the culture medium at 5 h post-treatment (Fig. 4b). FC2 and NC4 displayed very similar durability profiles with clear degradation seen up to 24 h (Fig. 4a), however, this was not maintained when the degrader was removed from the culture medium after 24 h (Fig. 4b). HaloPROTACs HV1 and HV2 were less durable in their response with depletion not maintained upon removal of the degrader from the culture medium and overall slower degradation kinetics (Fig. 4a, b).

To functionally assess the impact of the different tTPDs on a model substrate, we selected the well-characterised pro-necroptotic pseudokinase MLKL. MLKL is the terminal effector protein for necroptotic cell death^30^ which can be induced by the combined treatment of TNF, Smac mimetic (SM); compound A^31^ and z-VAD-fmk (TSZ)^32^. C-terminally tagged MLKL has previously been shown to be fully functional^33^ and MLKL knockout cells cannot undergo necroptosis unless reconstituted with an active MLKL construct^32^. We therefore generated C-terminal fusion proteins of MLKL containing either FKBP^F36V^, Halo or NanoLuc tags and stably expressed doxycycline-inducible versions of these proteins in *MLKL*^*−/−*^ HT29 cells that were generated by CRISPR/Cas9 targeting. The HT29 cell line is widely used and can undergo rapid necroptosis when stimulated with TSZ^32^. MLKL-NanoLuc expression was independently confirmed by NanoLuc luminescence (Supplementary Fig. 4a). The tagged versions of MLKL displayed reduced expression compared to untagged MLKL in *MLKL*^*−/−*^ HT29 cells, with the NanoLuc tag leading to a strong reduction in expression compared to adding a Halo or FKBP^F36V^ tag (Supplementary Fig. 4b). From this data it is unclear to what extent this will limit the application of the NanoLuc tag to other targets, and what causes the reduced expression. Nevertheless, the expression level of MLKL-NanoLuc was sufficient as MLKL-Halo, MLKL-FKBP^F36V^ and MLKL-NanoLuc could confer comparable sensitivity to necroptosis (Supplementary Fig. 4c).

The tagged MLKL substrates afforded us the opportunity to directly compare the efficiency of the CRBN and VHL degrader systems against a biologically-relevant substrate with the tags positioned at the same terminus (Fig. 4c). Interestingly the FV1 degrader that recruits F36V-VHL was faster at triggering degradation of MLKL compared to the F36V-CRBN degrader FC1, acting within 1 h (Supplementary Fig. 4d). FV1 was also more effective at triggering degradation of MLKL-FKBP^F36V^ compared to the F36V-CRBN degrader FC1 resulting in no detectable protein at 5 h at low nM concentrations (Fig. 4d). To directly compare whether the FKBP^F36V^ or Halo epitope tags could influence the degradation of tagged MLKL with the same CRL system we tested the F36V-VHL and Halo-VHL systems side-by-side (Fig. 4e). Consistent with our previous results we observed that the F36V-VHL-recruiting degrader FV1 was the most effective compound (Fig. 4f). Interestingly, we observed comparable degradation of murine MLKL-FKBP^F36V^ reconstituted into *Mlkl*^*−/−*^ mouse dermal fibroblasts (MDFs) with F36V-CRBN degraders (FC1, FC2) and the F36V-VHL degrader (FV1) (Supplementary Fig. 4e), suggesting that even slight changes in the substrate or cell type might influence the effectivity of degrader compounds. To explore this further, we inserted each tag into the genomic locus of the FBL gene that encodes for the protein Fibrillarin, which is exclusively expressed in the nucleolus. We performed concentration titrations and time courses on Fibrillarin-NanoLuc, Fibrillarin-Halo and Fibrillarin-FKBP^F36V^ expressing cell lines and calculated D_max_ values based on densitometry measurements (Supplementary Fig. 4f–m). Consistent with our results for MLKL and our fusion protein, we observed that FV1 was superior in degradation capacity compared to the other tag degraders displaying a D_max_ of ~95.9% after 10 h. FC1, HV1, HV2 and NC4 all demonstrated clear degradation of FBL over 10 h with calculated D_max_ values of ~70%, ~93.2%, ~94.8% and ~52.9%, respectively. Fibrillarin-NanoLuc predominantly localises to the nucleus (Supplementary Fig. 5a) suggesting that NC4 was capable of degrading substrates that primarily reside in the nucleus. To determine whether substrates in other cellular compartments could also be targeted by NC4 we generated a fusion construct containing NanoLuc and the plasma membrane transporter protein SLC38A2. We confirmed the location of NanoLuc-SLC38A2 in the membrane compartment (Supplementary Fig. 5b), and consistent with our previous results we observed a reduction in Nanoluc-SLC38A2 upon treatment with NC4 (Supplementary Fig. 5c).

### tTPD systems trigger physiologically relevant degradation

**Fig. 5:**
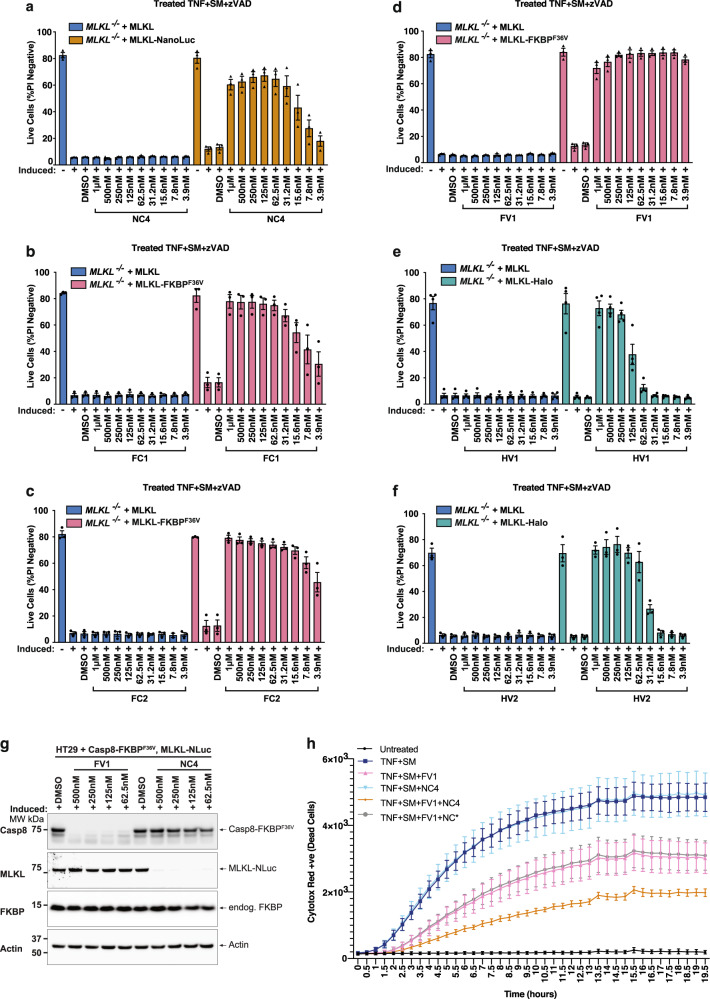
NanoTAC degraders trigger MLKL degradation and block necroptotic cell death. **a** *MLKL*^*−/−*^ HT29 cells stably expressing the doxycycline-inducible untagged MLKL or the C-terminal fusion protein MLKL- NanoLuc were induced with 40 ng/mL doxycycline overnight, then stimulated with NC4 (NanoLuc-CRBN) for 5 h before the addition of TNF (100 ng/mL) + Smac mimetic (compound A; 500 nM) + caspase inhibitor; z-VAD-fmk (10 μM), for 24 h. Cell death was assessed by flow cytometric analysis of PI exclusion. *N* = 3 independent experiments (symbols), EB represent mean ± SD. **b**–**d** *MLKL*^*−/−*^ HT29 cells expressing doxycycline-inducible untagged MLKL or the C-terminal fusion protein MLKL-FKBP^F36V^ were induced with 40 ng/mL doxycycline overnight, then stimulated with FC1, FC2 or FV1 for 5 h before the addition of TNF (100 ng/mL) + Smac mimetic (compound A; 500 nM) + caspase inhibitor; z-VAD-fmk (10 μM), for 24 h. Cell death was assessed by flow cytometric analysis of PI exclusion. *N* = 3 independent experiments (symbols), EB represent mean ± SD. **e**, **f** *MLKL*^*−/−*^ HT29 cells expressing doxycycline-inducible untagged MLKL or the C-terminal fusion protein MLKL-Halo were treated with 40 ng/mL doxycycline overnight to induce the constructs, then stimulated with the indicated concentrations HV1 or HV2 for 5 h before the addition of TNF (100 ng/mL) + Smac mimetic (compound A; 500 nM) + caspase inhibitor; z-VAD-fmk (10 μM), for 24 h. Cell death was assessed by flow cytometric analysis of PI exclusion. **e** *N* = 4, **f** *N* = 3 independent experiments (symbols), EB represent mean ± SD. **g** Western from *MLKL*^*−/−*^*CASP8*^*−/−*^ HT29 cells stably expressing the doxycycline-inducible C-terminal fusion protein, MLKL-NanoLuc (MLKL-NLuc) and constitutively expressing the C-terminal fusion protein caspase-8-FKBP^F36V^. Cells were induced with 40 ng/mL doxycycline overnight, then stimulated with FV1 and NC4 for 5 h. **h** Cells from **g** were treated with 40 ng/mL doxycycline and stimulated with 125 nM of FV1, NC4, or NC* for 5 h before the addition of TNF (100 ng/mL) + Smac mimetic (compound A; 500 nM) for 19.5 h. Cell death was assessed using an IncuCyte and Cytotox-Red uptake. *N* = 3 independent experiments, one representative experiment shown, EB represent mean ± SD. Source data are provided as a Source Data file.

To compare the biological activity of tTPD-mediated degradation across all tTPD platforms, we assessed whether our most potent NanoTAC could degrade substrates to a level that prevents necroptosis to achieve a response similar to protein knockout. Consistent with our previous results, NanoTAC degraders triggered efficient degradation of MLKL-NanoLuc, with NC4 displaying the most effective degradation profile (Supplementary Fig. 5d). Similar to the degradation of the Halo-EGFP-NanoLuc-FKBP^F36V^ fusion protein, NC4 triggered degradation of MLKL-NanoLuc within 2 h with sustained degradation over 10 h (Supplementary Fig. 5e). Impressively, NC4-induced degradation of MLKL-NanoLuc was sufficient to reduce MLKL-NanoLuc-driven necroptosis in HT29 cells in a dose-dependent manner (Fig. 5a), and importantly, NanoTAC degraders displayed no anti-necroptotic activity in *MLKL*^*−/−*^ cells expressing untagged MLKL (Fig. 5a) or wild-type HT29 cells (Supplementary Fig. 5f). F36V-CRBN targeting degraders (FC1, FC2) were capable of reducing necroptosis driven by MLKL-FKBP^F36V^ in *MLKL*^*−/−*^ HT29 cells in a dose-dependent manner, sustaining cell survival over 24 h at 62.5 and 15.6 nM, respectively (Fig. 5b, c). The F36V-VHL targeting degrader FV1 was extremely efficient at preventing necroptosis driven by MLKL-FKBP^F36V^ demonstrating a sustained and prolonged reduction in cell death at 3.9 nM over 24 h (Fig. 5d). HaloPROTACs (HV1 and HV2), although capable of reducing necroptosis driven by Halo-MLKL in *MLKL*^*−/−*^ HT29 cells, required much higher concentrations to maintain cell survival; 250 and 125 nM, respectively (Fig. 5e, f). Interestingly, HaloPROTACs also exhibited steep reductions in cell survival at lower concentrations, which may be attributed to the non-catalytic nature of these degrader compounds (Fig. 5e, f). Like NanoTAC degraders, FKBP^F36V^ degraders and HaloPROTACs displayed no anti-necroptotic activity in *MLKL*^*−/−*^ cells expressing untagged MLKL (Figs 5b–f) or wild-type HT29 cells (Supplementary Fig. 5g, h). A representative flow cytometry gating strategy that was used to analyse the induction of necroptosis is provided (Supplementary Fig. 5i). To test whether NanoTACs could be combined with other tTPD systems to modulate the levels of multiple proteins, we deleted endogenous caspase-8 via CRISPR/Cas9 targeting in HT29 cells expressing MLKL-NLuc, and re-expressed caspase-8-FKBP^F36V^. Both caspase-8-FKBP^F36V^ and MLKL-NLuc could be depleted by FV1 and NC4 treatment in these cells, respectively (Fig. 5g). TNF and Smac mimetic (TNF + SM) treatment triggers caspase-8 dependent apoptotic cell death that can be switched to necroptotic cell death when caspase-8 is inhibited or deleted from cells. Induction of MLKL-NLuc with doxycycline and constitutive expression of caspase-8-FKBP^F36V^ rendered this cell line susceptible to necroptotic cell death and apoptotic cell death upon treatment with TNF + SM (Fig. 5h). As expected, removal of MLKL-NLuc with NC4 did not impact cell death induced by TNF + SM treatment (TNF + SM + NC4), owing to active apoptotic caspase-8 signalling. Impressively, removal of caspase-8-FKBP^F36V^ with FV1 (TNF + SM + FV1) caused a clear reduction in TNF + SM induced cell death that could be further reduced with MLKL-NLuc removal with NC4, but not NC*. These data indicate that TNF + SM induced apoptosis and necroptosis driven by caspase-8-FKBP^F36V^ and MLKL-NLuc can be dynamically fine-tuned in the same cell system by combining two different tTPD degraders (Fig. 5h).

Collectively, these results suggest that efficient degradation of substrates with tag-targeting degraders is system dependent. Importantly, these results highlight that it is critical to compare each tTPD system against each substrate to identify the optimal degrader/tag pair to achieve prolonged and sustained degradation with minimal effective degrader concentration.

## Discussion

To add to the current toolbox of available tTPD systems and to compare the utility of different degrader/tag combinations we developed a new NanoLuc-targeted degrader system and a new reporter system. Our reporter system enables rapid comparative testing of optimal degrader/tag combinations that will advance the field by providing an interchangeable tag system that is amenable to a limitless number of degrader/tag pairs. We use this reporter system to test and develop NanoTACs, demonstrating that the reporter can facilitate the development of new tTPD systems. We believe this reporter system will increase the ease with which the powerful tTPD approach can be adopted and extended by other laboratories.

Using our reporter system and test substrates (MLKL & Fibrillarin) we provide side-by-side comparisons of the Halo-VHL, Halo-CRBN, F36V-CRBN and F36V-VHL tTPD systems, and directly compare these to the newly developed NanoLuc-CRBN and NanoLuc-VHL systems. We highlight the importance of comparing all tTPD systems against each substrate as each system does not degrade equally, and efficient tTPD-mediated degradation is extremely substrate-specific.

Interestingly, when we compared the degradation of human MLKL-FKBP^F36V^ and mouse MLKL-FKBP^F36V^ we observed that the CRBN-recruiting degraders were less effective at targeting human MLKL-FKBP^F36V^ compared to VHL degraders, yet mouse MLKL-FKBP^F36V^ was degraded by both systems equally. We also observed that endogenously tagged-Fibrillarin, which is localised in the nucleolus, was preferentially degraded by VHL-recruiting degraders compared to CRBN-recruiting degraders. This effect is potentially due to differences in the formation or stability of the ternary complex between the CRBN and VHL containing E3-ligase complexes and different substrate proteins, or subcellular location of VHL and CRBN, which may dictate substrate specificity for these E3-ligase complexes.

If a ternary complex is unstable or exhibits poor cooperative binding then certain degraders could be less efficient at triggering degradation, which would be consistent with previous reports that detail the importance of cooperativity for degrader-mediated protein degradation^4^. Our data highlights that each substrate needs to be assessed against all tTPD systems as it is extremely challenging to predict which substrate-CRL complex and which degrader/tag pair will lead to optimal degradation of a particular substrate. Another important consideration that may influence ubiquitination and substrate degradation is which protein terminus the tag is attached to as this may alter lysine accessibility. The optimal terminus for a particular tag is ultimately substrate-specific and some substrates cannot tolerate a tag attached to a particular terminus. However, if ubiquitin is primarily conjugated to the tag lysine residues rather than substrate lysines then the position of the tag would be unlikely to influence degradation but may still impact protein function.

Importantly, we find that the newly developed NanoLuc-CRBN degrader system functions comparably to the other tTPD systems, triggering degradation at low nM concentrations of degrader and to physiologically relevant levels. NanoTACs, however, also have some drawbacks that are important to highlight. We currently do not have efficient NanoLuc-VHL degraders in hand that would potentially increase the substrate scope of protein targets for NanoTAC degradation. NanoTACs are based on a NanoLuc inhibitor warhead, therefore, at intra-cellular concentrations approximating the IC_50_, NanoTACs interfere with nanoluciferase activity by inhibiting the conversion of furimazine to furimamide, preventing the ability to perform high-throughput degradation assays via a luminescence readout. Notably, since NanoTACs achieve efficient degradation of NLuc-tagged proteins resulting in a low cellular DC_50_, nanoluciferase luminescence can still be used to reliably read out degradation when used well below the NLuc IC_50_. However, for this purpose, we recommend that titrations are performed against the inactive analogue (NC*) and that critical controls i.e. MG132 or MLN4924 are included to separate degradation from NLuc inhibitory activity. Further investigation is also required to determine whether NanoTACs can be compatible with the in vivo nanoluciferase substrates^26^. NanoTACs are functionally similar to FKBP^F36V^-targeting degraders as NanoTACs are catalytic by nature and are not consumed during the reaction. The catalytic property of degraders is an important feature as degraders that exhibit poor cellular penetration or weak target-binding affinity can still be highly efficient degraders. It is, therefore, intriguing that NanoTACs are less effective compared to FKBP^F36V^-targeting degraders and might suggest that there are differences in the cellular accumulation of these molecules. The catalytic property of the NanoLuc/NanoTAC tTPD system is a major advantage over non-catalytic tTPD systems and further optimisation of NanoTACs to achieve improved degradation efficiency of NLuc-tagged substrates presents an exciting opportunity.

The importance of catalytic degraders for substate degradation was highlighted by elegant studies into Bruton’s tyrosine kinase (BTK) binding ibrutinib-derived degraders, demonstrating that reversible binding to BTK was essential to trigger BTK degradation^34,35^. It was postulated that irreversible covalent degraders such as HaloPROTACs are consumed once they engage and react with their target protein and hence cannot reach degradation efficiency comparable to catalytic degraders. Conversely, a number of studies argue against this theory:^18–20,36–38^. Furthermore, our data and those of others suggest that covalent HaloPROTACs can trigger efficient degradation of Halo-tagged substrates and we show the comparable activity of non-catalytic HaloPROTACs to catalytic NanoTAC degraders^8,18,19,24^. Moreover, HaloPROTACs have high affinity and selectivity for the Halo tag and we observed that HaloPROTACs maintained substrate depletion over 24 h.

Overall, we find that the FKBP^F36V^ tTPD systems are generally superior at triggering degradation of FKBP^F36V^ tagged substrates with regard to the minimal effective concentration and the time taken to achieve protein loss respective to controls, compared to the Halo- and NanoLuc-targeting systems, in our hands. Our observation that NanoTACs and HaloPROTACs degrade with slower kinetics and require higher minimal concentrations to achieve effective degradation compared to FKBP^F36V^ degraders might be explained by a variety of factors that alter the intracellular accumulation of these compounds including cell permeability, drug efflux pumps, cellular metabolism or interactions with intracellular proteins and other biomolecules. Another possible limitation in the effectiveness of HaloPROTACs is their potential reactivity with strong nucleophiles present in the cell (e.g. Glutathione) due to the chloroalkyl moiety. This irreversible side-reaction would consume the HaloPROTAC and decrease the effective concentration of the target Halo-tagged protein. HaloPROTACs and NanoTACs likely do not reach sufficient intracellular concentration in our necroptotic assays at lower assay concentrations, as our data show that these degraders are unable to provide complete protection against MLKL-Halo induced necroptosis.

The FKBP^F36V^ system is excellent for triggering degradation of FKBP^F36V^-tagged substrates, and the FKBP^F36V^ tag is small compared to the other tags at just 12 kDa, having minimal impact on fusion partner proteins. If targeted protein degradation is the primary goal, then this tTPD system is an ideal choice as the experimenter can easily and rapidly switch between the CRBN and VHL systems through the use of different degrader compounds. However, if the experimenter is interested in also understanding tagged protein biochemistry/biology, (cellular localisation, protein interactomes, tissue expression profiles ect.) then the NanoLuc (catalytic degrader available) and Halo (only non-catalytic degraders available) tags are superior as they offer the best of both worlds due to the numerous commercial tools available for these two protein tags.

Consistent with previous reports, our data detail that all current tag-targeting degraders trigger proteasomal degradation of tagged substrates. However, we did observe that the degradation of the reporter construct by FV1 was only partially reduced in the presence of the proteasome inhibitor MG132. Given that we pre-incubated the cells with MG132 for 1 hour prior to the addition of FV1, which is sufficient to block degradation triggered by the other tag-targeting degraders, it is possible that FV1 might also trigger degradation through alternative pathways, for example, the lysosomal degradation pathway. Alternatively, FV1 could simply be very efficient at triggering substrate ubiquitination and degradation, and this degradation might precede complete proteasomal inhibition by MG132, even after pre-incubation.

A comprehensive analysis in animals is yet to be conducted to compare the tTPD systems side-by-side, and in vivo pharmacokinetic and pharmacodynamic studies will need to be performed on the NanoTAC compounds. Regardless, studies that have tested individual tag-targeting degraders, or similar degraders such as RC32 comprised of the FKBP12 ligand Rapamycin conjugated to the CRBN ligand pomalidomide, in animals, detail that they are well tolerated and can trigger efficient depletion of substrates^8–11^. Notably, in vivo pharmacokinetic analysis and activity studies for the dTAG degraders FC1 and FV1 have been performed^9,10^, and one group has reported in vivo activity for one HaloPROTAC, HaloPROTAC-3^8^, which was not used in our study. Tag-targeting degrader compounds are not designed to be used therapeutically; however, tTPD systems do allow for the assessment of chemical-induced protein depletion in animal disease models, that will closely mimic targeted protein degradation of endogenous targets. With the advancement of the tTPD systems to include NanoLuc in the armamentarium, tTPD technology is leading the way for comprehensive validation studies to be conducted on prospective therapeutic targets to rationally determine which targets warrant drug discovery investment to identify ligands targeting equivalent endogenous proteins.

## Methods

### Cell culture and maintenance

HEK293T (ATCC; CRL3216), HT29 (ATCC; HTB-38) and immortalized (SV40 large-T antigen) mouse dermal fibroblast (iMDF) cells (generated in-house from C57BL/6 mouse tails) were cultured in Dubecco’s modified Eagle medium (DMEM) supplemented with 10% foetal bovine serum (FBS, Sigma), 50 U/mL penicillin (Gibco) and 50 μg/mL streptomycin (Gibco). iMDFs were isolated from C57BL/6 mouse tails and transformed with SV40 Large-T antigen. All cells were maintained at 37 °C with 10% CO_2_ in a humidified incubator.

### Plasmid constructs and stable cell lines

The cDNA for human or mouse *Mlkl* was cloned from synthetic gBlock fragments (Integrated DNA Technologies) to generate fusion proteins harbouring a Halo, FKBP^F36V^ or NanoLuc (NLuc) tag at the C-terminus of MLKL. The individual tags in the Halo-EGFP-NanoLuc-FKBP^F36V^ (H-E-N-F) reporter were ordered as gBlock fragments (Integrated DNA Technologies) and made into a single reporter construct using restriction digestion and ligation cloning. Halo refers to HaloTag7, FKBP^F36V^ refers to FKBP12^F36V^. Each protein in the reporter construct is separated by a 9-10 amino acid glycine-serine linker. To generate the H-FF-N-F reporter construct, the EGFP was excised from the H-E-N-F reporter construct and replaced with Firefly luciferase (kind gift from Joan Heath) using In-Fusion cloning (Takara Bio). *MLKL*^*-/-*^ HT29 cells were generated by CRISPR-Cas9 targeting using lentiviral transduction, reported previously^32^, while *Mlkl*^*-/-*^ mouse dermal fibroblasts (MDFs) were generated from *Mlkl*^*−/−*^ mice described previously^39^. Stable cell lines were generated using the Lentiviral packaging constructs (VSVg, RSV-Rev, pMDL) and the pFTRE3G doxycycline-inducible vector and selected with Puromycin (2 μg/mL). pX330A-FBL/PITCh was a gift from Takashi Yamamoto (Addgene plasmid # 63671; http://n2t.net/addgene:63671; RRID:Addgene_63671)^40^. pCRIS-PITChv2-FBL was a gift from Takashi Yamamoto (Addgene plasmid # 63672; http://n2t.net/addgene:63672; RRID:Addgene_63672)^40^. pCRIS-PITChv2-FBL was modified to contain either the NanoLuc, Halo or FKBP^F36V^ tag sequences to enable genomic insertion of a tag-T2A-PuroR sequence at the FBL locus to produce C-terminal NanoLuc-, Halo- or FKBP^F36V^- tagged-Fibrillarin. For knock-in cell lines, 63671 or modified 63,672 vectors were co-transfected into 293Ts with lipofectamine, stably integrated cells were selected with puromycin (1 μg/ml).

### NanoLuc and Firefly luciferase assays

For high-throughput assessment of tTPD-mediated degradation, cells expressing NanoLuc or Firefly fusion proteins were seeded into 384-well flat bottom, clear bottom white-walled plates (Corning) at 1–1.5 × 10^4^ cells/well in 50 μL of DMEM/FCS. A final concentration of 20 ng/mL and 40 ng/mL of doxycycline was used for HEK293T and HT29 cells, respectively, followed by overnight incubation (16–24 h) to induce construct expression. Cells were then treated with either vehicle control (DMSO) or degrader compound as indicated. In compound titration experiments, all vehicle control amounts were equivalent to the highest degrader concentration. For MG132 and MLN4924 rescue experiments, cells were pre-treated for 30 min–1 h with MG132 (10 μM, Selleck Chemicals) or MLN4924 (1–5 μM; Tocris) prior to the addition of the degrader compounds. Time-course experiments were conducted as reverse time courses so that luminescence could be detected at the same cell density. At the stated timepoints, luminescence was induced using the Nano-GLO Luciferase assay system (Promega) for NanoLuc luminescence, or Nano-Glo Dual-Luciferase Reporter assay system (Promega) for NanoLuc/Firefly dual luminescence. Clear plate bottoms were taped with coloured tape to prevent bleeding between wells. For IC_50_ measurements recombinant NanoLuc enzyme was prepared to a concentration of 0.2 nM, and NanoLuc LCS dilution buffer was diluted 1:30 in TBS + 0.01% BSA. NC4 or NanoLuc inhibitor 2 were diluted in the LCS solution and combined with the diluted NanoLuc enzyme. Samples were incubated for 6 min at room temperature before assaying for luminescence. Luminescence was then measured (0.1 s/well, filter 470-480) on a microplate reader (CLARIOstar Plus, BMG Labtech).

### Immunoblotting

Cells were seeded in 24-well plates ±doxycycline (20 ng/mL for HEK293T, 40 ng/mL for HT29) and incubated overnight (16–24 h) prior to degrader treatments, as stated in the Fig. legends. All timepoint experiments were performed as reverse timepoints in order to harvest all cells at the same time. At the indicated time points cells were lysed in NuPAGE LDS lysis buffer (Invitrogen) diluted to 1× in DISC lysis buffer (20 mM Tris-HCl pH7.5, 150 mM NaCl, 2 mM EDTA pH 8.0, 1% Triton X-100, 10% glycerol, H_2_0)) supplemented with β-Mercaptoethanol (2%), protease and phosphatase inhibitors. Lysates were run through polypropylene columns (Pierce) to shred DNA. Proteins were separated by SDS-PAGE on 4-12% gradient gels (Invitrogen) and transferred onto Immoblon-E polyvinyl difluoride membranes (Merck). Membranes were blocked in 5% skim milk (Devondale) in TBS-T (TBS, 0.1% Tween-20) for 1 h prior to immunoblotting with primary antibodies overnight at 4 °C. Unless stated otherwise, all primary antibodies were diluted in TBS-T (TBS, 0.1% Tween-20) containing 5% BSA (Sigma A8022) and 0.04% sodium azide: HaloTag (1:1000, Promega; G9211), NanoLuc (1:500, Promega; N7000), β-actin (1:20,000, Sigma; A-1798), MLKL (1:1000, produced in-house; 3H1 clone)^39^, FKBP (1:1000, R&D systems; 422513), caspase-8 (1:1000, Proteintech; 13423-1-AP), Histone H3 (1:1000, Abcam; ab10799), Cadherin (1:1000, Cell Signalling Technologies; 4068 T). Membranes were washed 3 × 5–10 min in TBS-T (TBS, 0.1% Tween-20) prior to the addition of the appropriate secondary antibodies conjugated with horseradish peroxidase (Jaxon laboratories). All secondary antibodies were diluted at 1:10,000 in TBS-T (TBS, 0.1% Tween-20) containing 5% skim milk and incubated at room temperature for 1 h. Final washes of 4 × 5–10 min with TBS-T (TBS, 0.1% Tween-20) were conducted before ECL development (Millipore, Bio-Rad) and protein detection using the ChemiDoc Touch Imaging System (Bio-Rad). All images were processed using Image Lab software.

### Necroptosis assays

To simulate necroptosis, cells were seeded into 96-well plates ±40 ng/mL doxycycline, to induce construct expression, and treated with 100 ng/mL FLAG-TNF (recombinant human, in-house), 500 nM compound A Smac mimetic (kindly gifted by TetraLogics Pharmaceuticals) and 5 μM IDN-6556 (Cayman) or 10 uM Z-VAD-fmk (Selleckchem) overnight (16–24 h). Degrader compounds or a DMSO vehicle control were added for 5 h, as stated in the Fig. legends. Cells were detached using Trypsin-EDTA (Merck) and resuspended in cell supernatants containing 10 μg/mL propidium iodide (PI). PI exclusion analysis was performed using an LSR II flow cytometer (Becton Dickinson, NJ) with 10,000 single-cell events per sample. Flow cytometry data were analysed using WEASEL version 2.7 software (Frank Battye).

### Quantitative proteomics

Cell pellets were extracted with 5% SDS (including 100 mM TEAB) and processed using micro S-traps as described by the manufacturer (Protifi). For liquid chromatography-tandem mass spectrometry (LC-MS/MS) analysis, approximately 200 ng of sample was injected onto an Acuity M-class UPLC (Waters) connected to a timsTOF pro II (Bruker). Peptides were separated using a 112 min gradient (solvent A, 0.1% formic acid; solvent B, 99.9% acetonitrile/0.1% formic acid) on a C18 analytical column (IonOpticks, Aurora Series Emitter Column, AUR2-25075C18A 25 cm × 75 µm). Data-dependent PASEF acquisition was performed (100–1700 *m/z* scan range and 0.6–1.6 mobility range) and the data searched against the reviewed Homo sapiens uniprot database (UP000005640) with MSfragger (v3.1) within the Fragpipe framework (v17.0) using strict trypsin cleavage and up to two missed cleavages. Precursor and fragment ion tolerances were both set to 20 ppm and the minimum peptide length set at seven. Peptide and protein level FDR was set at 1% and protein quantification determined using the MaxLFQ algorithm40. Data were processed and visualised with the DEP R package, where statistical significance was determined using the moderated *t*-statistic from the limma package 41. Unadjusted *P*-values were plotted and statistical significance was determined using the density-based q-value in fdrtools^41^.

### Animal handling

C57BL/6 J mice were maintained in-house under specific pathogen-free conditions at the Walter and Eliza Hall Institute of Medical Research (WEHI), Australia. Animal rooms were maintained at approximately 21 °C ± 3 °C at 40–70% humidity with a timed 14/10 h light-dark cycle. Wild-type C57BL/6 J mice were bred at WEHI and/or obtained from WEHI animal supplies (Kew, Australia). None of the mice used in our experiments had been previously used for other procedures. The animals presented with a healthy status and were selected independently of their gender for generating MDFs. Female and male mice were at least 6 weeks old at the time of experimentation. All procedures for this study were approved by the Walter and Eliza Hall Institute (WEHI) Animal Ethics Committee, Australia. All research complied with all relevant ethical regulations for animal testing and research.

### Generation of NanoTAC heterobifunctional compounds

See supplementary methods.

### Reporting summary

Further information on research design is available in the Nature Research Reporting Summary linked to this article.

## Supplementary information

Supplementary Information

Reporting Summary

## Data Availability

The data that support this study are available from the Reagents are available upon request. All NanoTACs are available upon request. Uncropped Western blots are provided in the source data file. The mass spectrometry proteomics data generated in this study have been deposited in the ProteomeXchange Consortium via the PRIDE^42^ partner repository with the dataset identifier PXD031371. Source data are provided with this paper.

## Source data

Source Data

## References

[1] Burslem GM, Crews CM (2020). Proteolysis-targeting chimeras as therapeutics and tools for biological discovery. Cell.

[2] Komor AC, Badran AH, Liu DR (2017). CRISPR-based technologies for the manipulation of eukaryotic genomes. Cell.

[3] Khera N, Rajput S (2017). Therapeutic potential of small molecule inhibitors. J. Cell Biochem..

[4] Gadd MS (2017). Structural basis of PROTAC cooperative recognition for selective protein degradation. Nat. Chem. Biol..

[5] Cyrus K (2011). Impact of linker length on the activity of PROTACs. Mol. Biosyst..

[6] Zorba A (2018). Delineating the role of cooperativity in the design of potent PROTACs for BTK. Proc. Natl Acad. Sci. USA.

[7] Yesbolatova A, Tominari Y, Kanemaki MT (2019). Ligand-induced genetic degradation as a tool for target validation. Drug Discov. Today Technol..

[8] BasuRay S, Wang Y, Smagris E, Cohen JC, Hobbs HH (2019). Accumulation of PNPLA3 on lipid droplets is the basis of associated hepatic steatosis. Proc. Natl Acad. Sci. USA.

[9] Nabet B (2020). Rapid and direct control of target protein levels with VHL-recruiting dTAG molecules. Nat. Commun..

[10] Nabet B (2018). The dTAG system for immediate and target-specific protein degradation. Nat. Chem. Biol..

[11] Sun X (2019). A chemical approach for global protein knockdown from mice to non-human primates. Cell Discov..

[12] Nishimura K, Fukagawa T, Takisawa H, Kakimoto T, Kanemaki M (2009). An auxin-based degron system for the rapid depletion of proteins in nonplant cells. Nat. Methods.

[13] Simpson LM (2020). Inducible degradation of target proteins through a tractable affinity-directed protein missile system. Cell Chem. Biol..

[14] Okitsu K (2018). Development of a small hybrid molecule that mediates degradation of His-Tag fused proteins. J. Med. Chem..

[15] Bond AG (2021). Development of BromoTag: a “Bump-and-Hole”-PROTAC system to induce potent, rapid, and selective degradation of tagged target proteins. J. Med. Chem..

[16] Erb MA (2017). Transcription control by the ENL YEATS domain in acute leukaemia. Nature.

[17] Winter GE (2015). DRUG DEVELOPMENT. Phthalimide conjugation as a strategy for in vivo target protein degradation. Science.

[18] Buckley DL (2015). HaloPROTACS: use of small molecule PROTACs to induce degradation of HaloTag fusion proteins. ACS Chem. Biol..

[19] Tovell H (2019). Rapid and reversible knockdown of endogenously tagged endosomal proteins via an optimized HaloPROTAC degrader. ACS Chem. Biol..

[20] Tomoshige S, Hashimoto Y, Ishikawa M (2016). Efficient protein knockdown of HaloTag-fused proteins using hybrid molecules consisting of IAP antagonist and HaloTag ligand. Bioorg. Med. Chem..

[21] Clackson T (1998). Redesigning an FKBP-ligand interface to generate chemical dimerizers with novel specificity. Proc. Natl Acad. Sci. USA.

[22] Los GV (2008). HaloTag: a novel protein labeling technology for cell imaging and protein analysis. ACS Chem. Biol..

[23] Ohana RF (2009). HaloTag7: a genetically engineered tag that enhances bacterial expression of soluble proteins and improves protein purification. Protein Expr. Purif..

[24] Caine EA (2020). Targeted protein degradation phenotypic studies using HaloTag CRISPR/Cas9 endogenous tagging coupled with HaloPROTAC3. Curr. Protoc. Pharmacol..

[25] England CG, Ehlerding EB, Cai W (2016). NanoLuc: a small luciferase is brightening up the field of bioluminescence. Bioconjug. Chem..

[26] Su Y (2020). Novel NanoLuc substrates enable bright two-population bioluminescence imaging in animals. Nat. Methods.

[27] Walker JR (2017). Highly potent cell-permeable and impermeable NanoLuc luciferase inhibitors. ACS Chem. Biol..

[28] Pettersson M, Crews CM (2019). PROteolysis TArgeting Chimeras (PROTACs)—past, present and future. Drug Discov. Today Technol..

[29] Foley CA, Potjewyd F, Lamb KN, James LI, Frye SV (2020). Assessing the cell permeability of bivalent chemical degraders using the chloroalkane penetration assay. ACS Chem. Biol..

[30] Murphy JM (2020). The killer pseudokinase mixed lineage kinase domain-like protein (MLKL). Cold Spring Harb. Perspect. Biol..

[31] Vince JE (2007). IAP antagonists target cIAP1 to induce TNFalpha-dependent apoptosis. Cell.

[32] Petrie EJ (2018). Conformational switching of the pseudokinase domain promotes human MLKL tetramerization and cell death by necroptosis. Nat. Commun..

[33] Tanzer MC (2016). Evolutionary divergence of the necroptosis effector MLKL. Cell Death Differ..

[34] Guo WH (2020). Enhancing intracellular accumulation and target engagement of PROTACs with reversible covalent chemistry. Nat. Commun..

[35] Tinworth CP (2019). PROTAC-mediated degradation of Bruton’s tyrosine kinaseis inhibited by covalent binding. ACS Chem. Biol..

[36] Bond MJ, Chu L, Nalawansha DA, Li K, Crews CM (2020). Targeted degradation of oncogenic KRAS(G12C) by VHL-recruiting PROTACs. ACS Cent. Sci..

[37] Gabizon R (2020). Correction to efficient targeted degradation via reversible and irreversible covalent PROTACs. J. Am. Chem. Soc..

[38] Xue G (2020). Protein degradation through covalent inhibitor-based PROTACs. Chem. Commun. (Camb.).

[39] Murphy JM (2013). The pseudokinase MLKL mediates necroptosis via a molecular switch mechanism. Immunity.

[40] Sakuma T, Nakade S, Sakane Y, Suzuki KT, Yamamoto T (2016). MMEJ-assisted gene knock-in using TALENs and CRISPR-Cas9 with the PITCh systems. Nat. Protoc..

[41] Strimmer K (2008). fdrtool: a versatile R package for estimating local and tail area-based false discovery rates. Bioinformatics.

[42] Perez-Riverol Y (2022). The PRIDE database resources in 2022: a hub for mass spectrometry-based proteomics evidences. Nucleic Acids Res.

