# Supplementary material for: Development of NanoLuc-targeting protein degraders and a universal reporter system to benchmark tag-targeted degradation platforms
Source: Nat Commun. 2022 Apr 19;13:2073. doi: 10.1038/s41467-022-29670-1 (PMC9019100; doi:10.1038/s41467-022-29670-1)
Supplement: Supplementary file 1 — Supplementary Information [file 41467_2022_29670_MOESM1_ESM.pdf]

Supplementary Information: Development of NanoLuc-targeting protein degraders and a universal reporter system to benchmark tag-targeted degradation platforms

R. Feltham et al.

SUPPLEMENTARY FIGURES

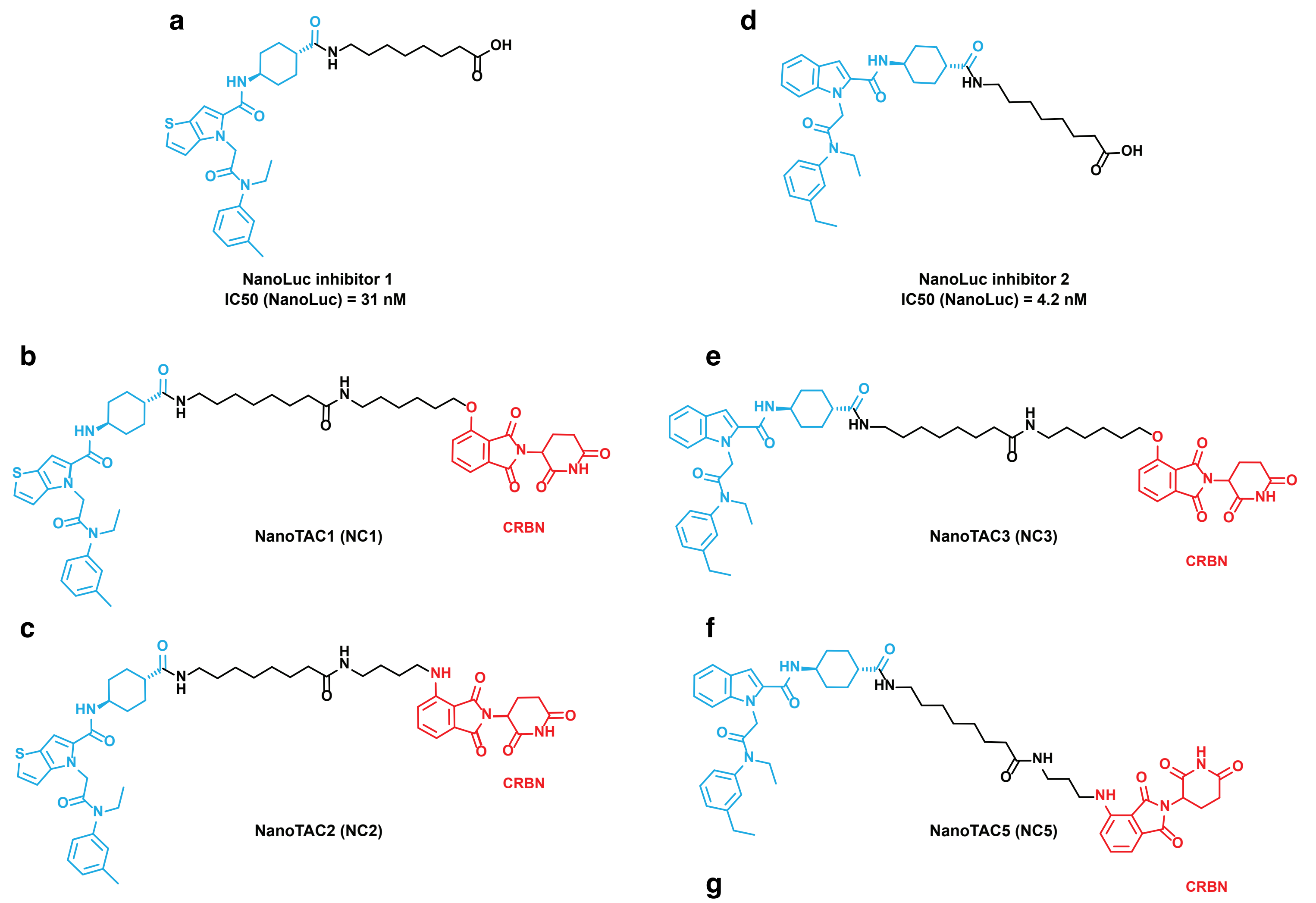

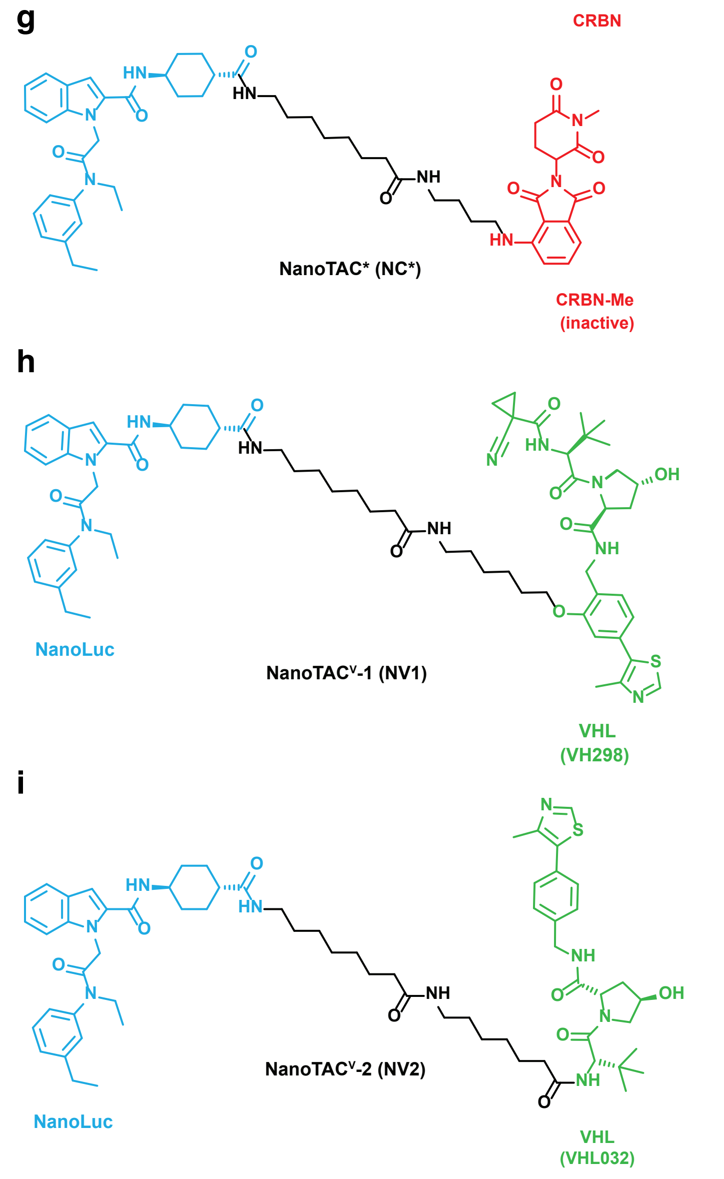

Supplementary Figure 1. Chemical structure of the NanoLuc inhibitors and NanoTACs

a. NanoLuc inhibitor 1. **b.** NanoTAC1 (NC1). **c.** NanoTAC2 (NC2). **d.** NanoLuc inhibitor 2. **e.** NanoTAC3 (NC3). **f.** NanoTAC5 (NC5). **g.** NanoTAC* (NC*), inactive control for CRBN recruitment. **h.** NanoTACV-1 (NV1). **i.** NanoT--ACV-2 (NV2).

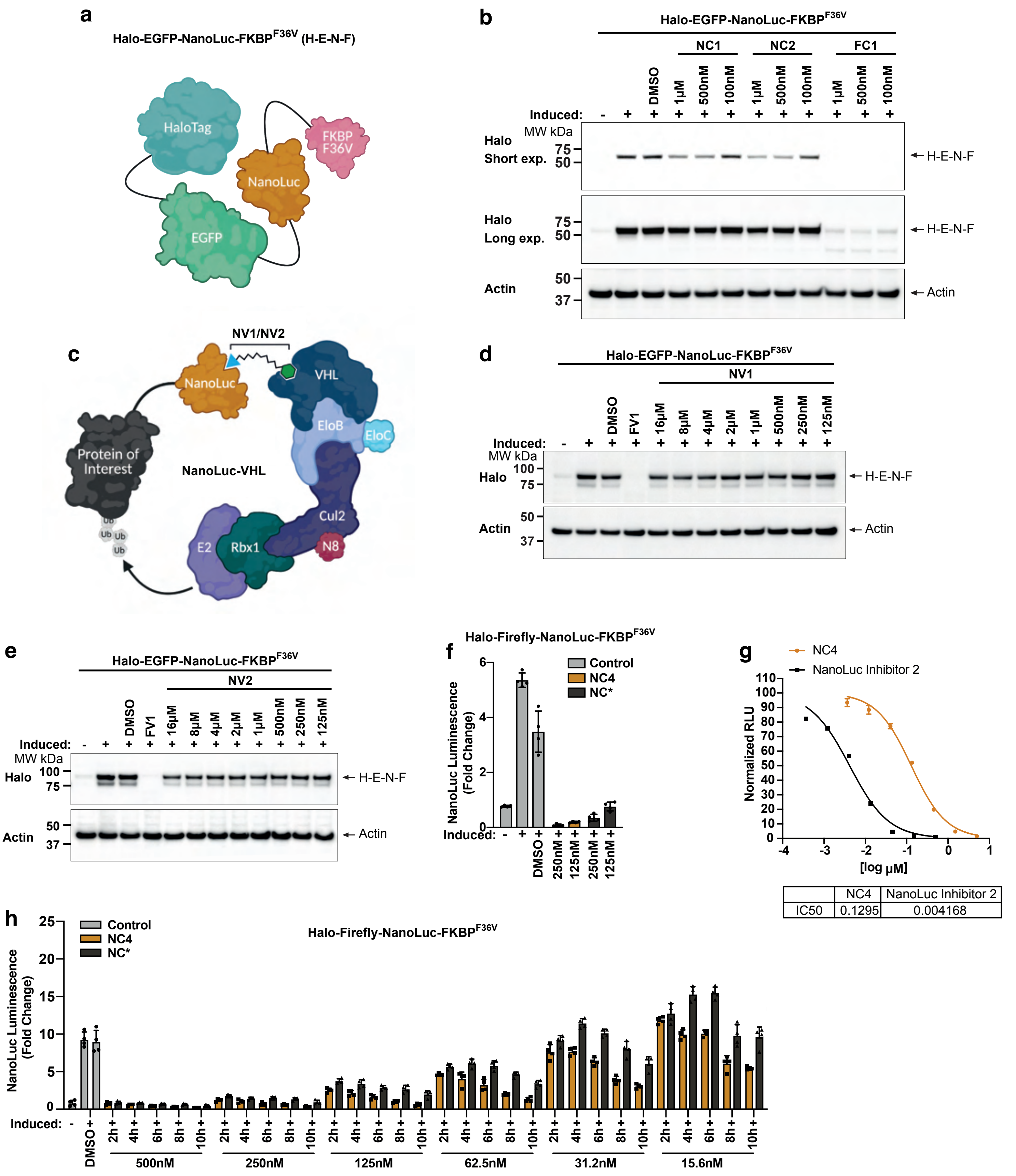

Supplementary Figure 2

Supplementary Figure 2. Identification of NC4 as a potent NanoTAC degrader

a. Schematic depicting the reporter construct H-E-N-F. **b.** Western blot analysis of total cell lysates from 293T cells stably expressing H-E-N-F, treated with 20 ng/mL doxycycline overnight (induced), then stimulated with the indicated concentrations of NC1, NC2 (NanoLuc-CRBN) or FC1 (F36V-CRBN) degrader compounds for 5 hours. **c.** Schematic depicting the NanoLuc-CRL2^{VHL} tTPD system. **d.** Western blot analysis of total cell lysates from 293T cells expressing H-E-N-F, treated with 20 ng/mL doxycycline overnight (induced), then stimulated with the indicated concentrations of NV1 or FV1 for 5 hours. **e.** Western blot analysis of total cell lysates from 293T cells expressing H-E-N-F, treated with 20 ng/mL doxycycline overnight (induced), then stimulated with the indicated concentrations of NV2 or FV1 for 5 hours. **f.** NanoLuc luminescence from cells expressing H-FF-N-F treated with 20 ng/mL doxycycline overnight (induced), then stimulated with DMSO or the indicated concentrations of NC4 (NanoLuc-CRBN) or NC*(NanoLuc-CRBN inactive) for 5 hours. **g.** Normalised relative light units (RLU) from recombinant NanoLuc enzyme incubated with NC4 or NanoLuc inhibitor 2 for 6 minutes. Where error bars are not shown this indicates that the symbol size is larger than the error bar. Experiment repeated independently 3 times. **h.** NanoLuc luminescence from HEK293T cells expressing H-FF-N-F, treated with 20 ng/mL doxycycline overnight (induced), then stimulated with the indicated concentrations of NC4 and NC* for the indicated times. **f, h.** EB represent mean ± SD from N = 4 (symbols), technical repeats. All experiments were repeated independently 3 times, and a representative figure is shown. Source data are provided as a Source Data file.

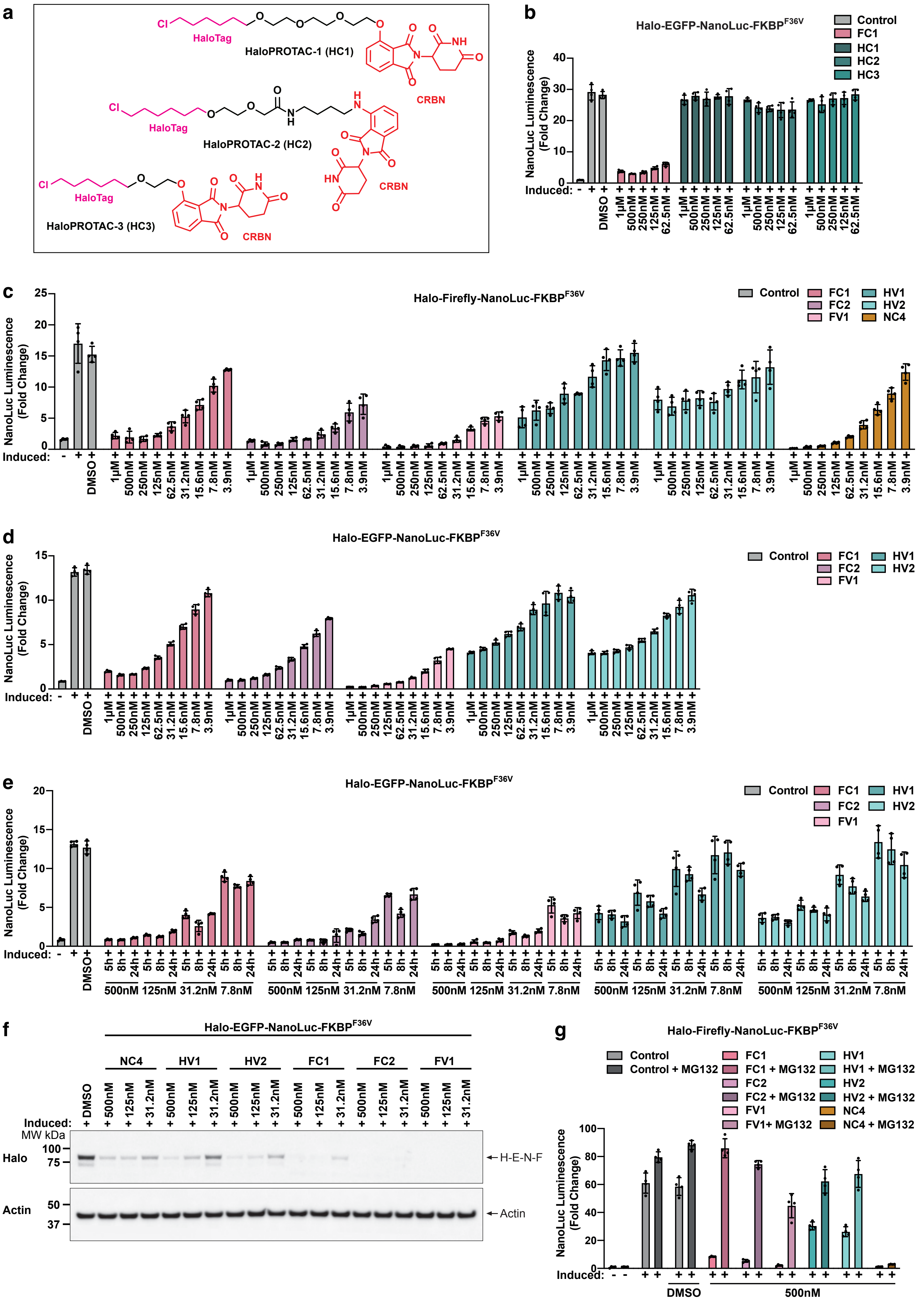

Supplementary Figure 3

Supplementary Figure 3. FKBP^{F36V} tTPD systems outperform other tTPD systems

a. Chemical structures of Halo-CRBN heterobifunctional degrader compounds; HaloPROTAC-1 (HC1), HaloPROTAC-2 (HC2), HaloPROTAC-3 (HC3). **b.** NanoLuc luminescence from 293T cells stably expressing doxycycline-inducible H-E-N-F, treated with 20 ng/mL doxycycline overnight (induced), then stimulated with the indicated concentrations HC1, HC2, HC3 and FC1. **c.** NanoLuc luminescence from cells stably expressing doxycycline-inducible H-FF-N-F, treated with 20 ng/mL doxycycline overnight (induced), following treatment with 20 μM MG132 for 1 hour prior to stimulation with the indicated concentrations of FC1 (F36V-CRBN), FC2 (F36V-CRBN), FV1 (F36V-VHL), HV1 (Halo-VHL), HV2 (Halo-VHL) or NC4 (NanoLuc-CRBN) for 5 hours. **d.** NanoLuc luminescence from 293T cells stably expressing doxycycline-inducible H-E-N-F, treated with 20 ng/mL doxycycline overnight (induced), then stimulated with the indicated concentrations FC1 (F36V-CRBN), FC2 (F36V-CRBN), FV1 (F36V-VHL), HV1 (Halo-VHL) or HV2 (Halo-VHL) for 5 hours. **e.** NanoLuc luminescence from 293T cells stably expressing doxycycline-inducible H-E-N-F, treated with 20 ng/mL doxycycline overnight (induced), then stimulated with the indicated concentrations FC1 (F36V-CRBN), FC2 (F36V-CRBN), FV1 (F36V-VHL), HV1 (Halo-VHL) or HV2 (Halo-VHL) for the indicated times. **f.** Western blot analysis of total cell lysates from cells expressing H-E-N-F, treated with 20 ng/mL doxycycline overnight (induced), then stimulated with the indicated concentrations of degraders for 4 hours. **g.** NanoLuc luminescence from cells stably expressing doxycycline-inducible H-FF-N-F, treated with 20 ng/mL doxycycline overnight (induced), following treatment with 20 μM MG132 for 1 hour prior to stimulation with the indicated concentrations of FC1 (F36V-CRBN), FC2 (F36V-CRBN), FV1 (F36V-VHL), HV1 (Halo-VHL), HV2 (Halo-VHL) or NC4 (NanoLuc-CRBN) for 5 hours. **b, c, d, e, g.** EB represent mean ± SD from N = 4 technical repeats. All experiments were repeated independently 3 times, and a representative figure is shown. Source data are provided as a Source Data file.

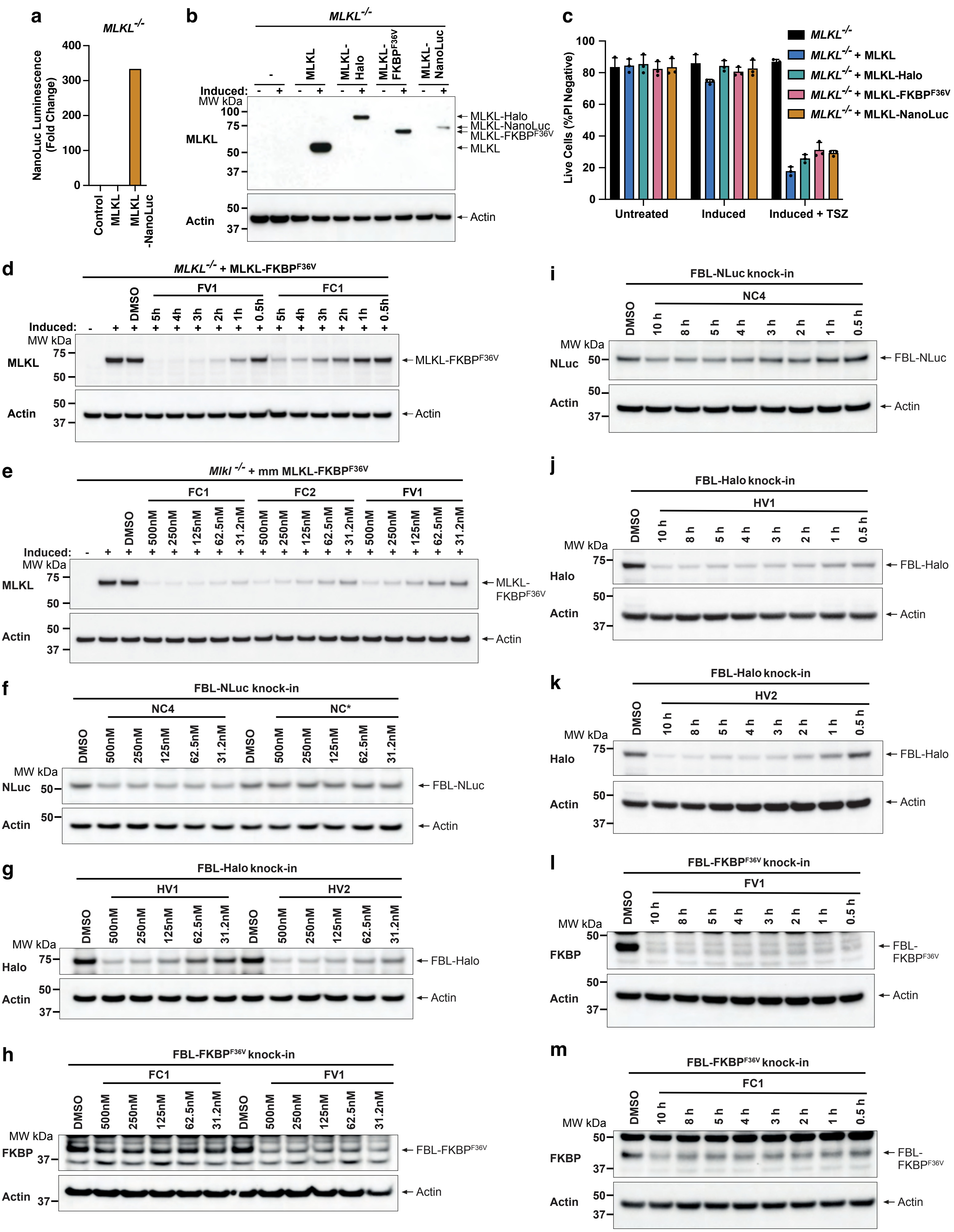

Supplementary Figure 4

Supplementary Figure 4. F36V-VHL degrader outperforms CRBN and Halo targeting degraders

a. NanoLuc luminescence from *MLKL*$^{-/-}$ HT29 cells (ctrl) and cells stably expressing doxycycline-inducible untagged MLKL or the C-terminal fusion protein MLKL-NanoLuc, treated with 40 ng/mL doxycycline (induced). **b.** Western blot analysis of total cell lysates from *MLKL*$^{-/-}$ HT29 cells stably expressing doxycycline-inducible untagged MLKL or C-terminal fusion proteins; MLKL-Halo, MLKL-FKBP^{F36V} and MLKL-NanoLuc, treated ± 40 ng/mL doxycycline (induced). **c.** *MLKL*$^{-/-}$ HT29 cells stably expressing doxycycline-inducible untagged MLKL or the C-terminal fusion proteins MLKL-Halo, MLKL-FKBP^{F36V} and MLKL-NanoLuc were treated ± 40 ng/mL doxycycline overnight to induce the constructs before the addition of TNF (100 ng/mL) + Smac mimetic (compound A; 500 nM-1 μM) + caspase inhibitor; z-VAD-fmk (10 μM) (TSZ), for 24 hours. Cell death was assessed by flow cytometric analysis of PI exclusion. N = 3 independent experiments (symbols), EB represent mean + SD. **d.** Western blot analysis of total cell lysates from *MLKL*$^{-/-}$ HT29 cells stably expressing the doxycycline-inducible C-terminal fusion protein MLKL-FKBP^{F36V}. Cells were treated with 40 ng/mL doxycycline overnight to induce the constructs, then stimulated with the 125 nM FV1 (F36V-VHL) or FC1 (F36V-CRBN). Representative figure from N = 3 independent experiments is shown. **e.** Western blot analysis of total cell lysates from *Mlkl*$^{-/-}$ mouse dermal fibroblasts stably expressing the doxycycline-inducible C-terminal fusion protein of murine MLKL-FKBP^{F36V}. Cells were treated with 40 ng/mL doxycycline overnight to induce the construct, then stimulated with FC1 (F36V-CRBN), FC2 (F36V-CRBN) or FV1 (F36V-VHL) for 5 hours. Representative figure from N = 3 independent experiments is shown. **f-h.** Western blot analysis of total cell lysates from 293T cells with the genomic locus of the FBL gene modified to express C-terminal fusions of Fibrillarin-Halo, Fibrillarin-NLuc or Fibrillarin-FKBP^{F36V}. Cells were stimulated for 5 hours. Representative figure from N = 3 independent experiments is shown. **i-m.** Western blot analysis of total cell lysates from 293T cells with the genomic locus of the FBL gene modified to express C-terminal fusions of Fibrillarin-Halo, Fibrillarin-NLuc or Fibrillarin-FKBP^{F36V}. Cells were stimulated with 500 nM of degrader compound. Representative figure from N = 3 independent experiments is shown. Source data are provided as a Source Data file.

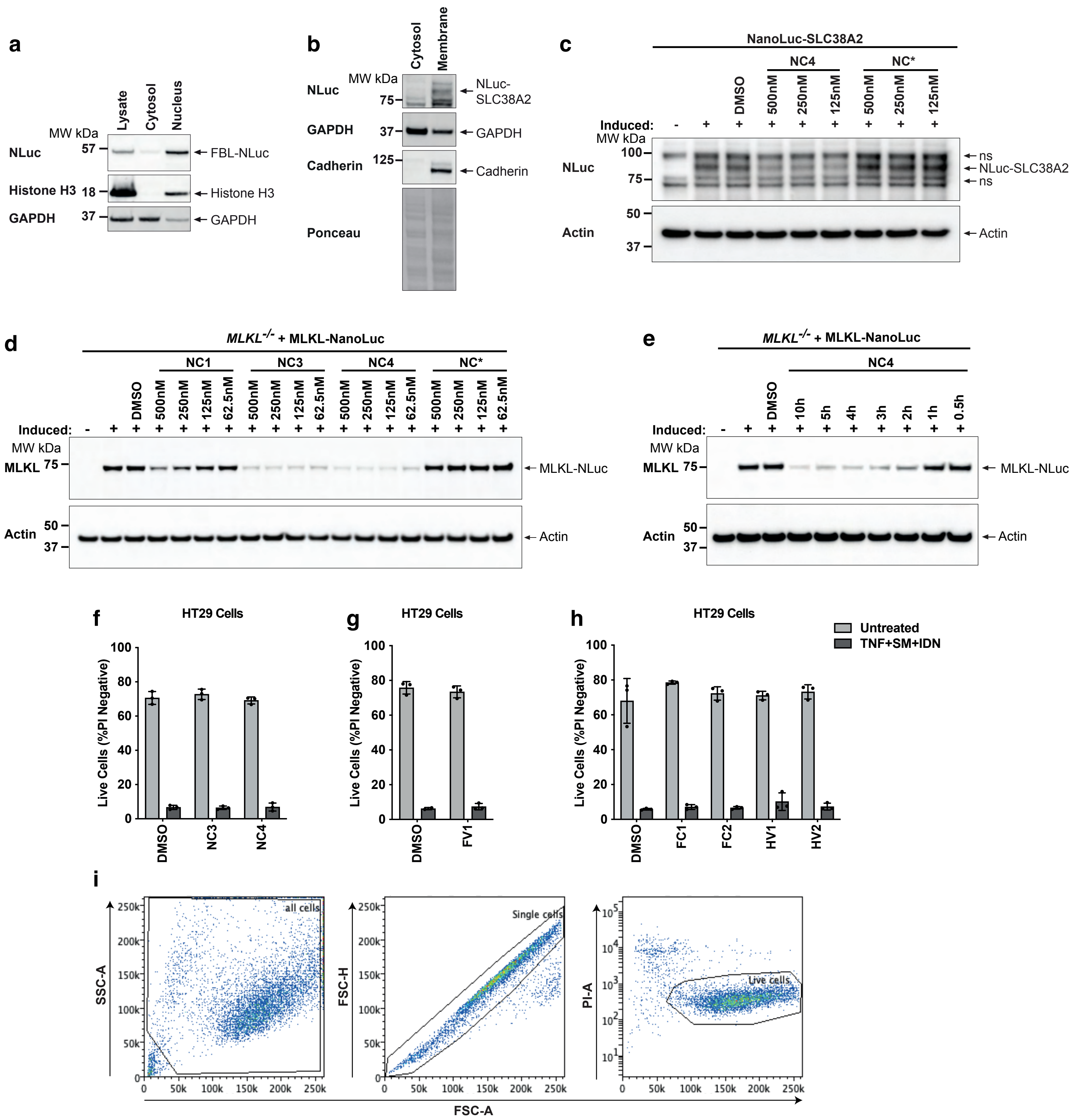

Supplementary Figure 5

Supplementary Figure 5. NanoTAC degraders trigger MLKL degradation and block necroptotic cell death

a. Western of cytosolic and nuclear fractions from 293T cells expressing Fibrillarin-FBL (from Figure S4). Data are representative of N = 3 independent experiments. **b.** Western of cytosolic and membrane fractions from 293T cells expressing NLuc-SLC38A2. Data are representative of N = 3 independent experiments. **c.** Western of 293T cells expressing NLuc-SLC38A2. Cells were induced with 20 ng/mL doxycycline overnight, then treated with DMSO or NC4 or NC* for 5 hours. Data are representative of N = 3 independent experiments. **d.** Western of lysates from *MLKL*$^{-/-}$ HT29 cells stably expressing the C-terminal fusion protein, MLKL-NanoLuc (MLKL-NLuc). Cells induced with 40 ng/mL doxycycline overnight, then stimulated for 5 hours. Data are representative of N = 3 independent experiments. **e.** Western cell lysates from A, cells were treated with 40 ng/mL doxycycline overnight, then stimulated with 125 nM of NC4. Data are representative of N = 3 independent experiments. **f-h.** HT29 cells were stimulated with DMSO (control) or 500 nM of degrader for 5 hours before the addition of TNF (100 ng/mL) + Smac mimetic (compound A; 500 nM-1 μM) + caspase inhibitor; Emricasan (IDN) (5 μM), for 24 hours. Cell death was assessed by flow cytometric analysis of PI exclusion. N = 3 replicates (symbols) is shown, EB; mean ± SD. Data are representative of N = 3 independent experiments. **i.** Representative flow cytometry gating strategy. Source data are provided as a Source Data file.

SUPPLEMENTARY METHODS

When requesting compounds used in this study please reference the WEHI-# code (Table 1).

Supplementary Table 1. WEHI numbers for requesting compounds

Ab	Name	WEHI #
NC1	NanoTAC1	WEHI-1655689
NC2	NanoTAC2	WEHI-1655698
NC3	NanoTAC3	WEHI-1880458
NC4	NanoTAC4	WEHI-1880459
NC5	NanoTAC5	WEHI-1880797
NC*	NanoTAC*	WEHI-1880796
NV1	NanoTAC^{V}1	WEHI-1880456
NV2	NanoTAC^{V}2	WEHI-1881306

General chemistry

Anhydrous solvents were obtained commercially (SIGMA-ALDRICH, Missouri, USA) and used without further purification. HATU and DIPEA were obtained commercially (CHEM-IMPEX, Illinois, USA) and used as supplied. All other commercial reagents were used as supplied. All non-aqueous reactions were performed in oven-dried glassware under inert atmosphere (nitrogen gas), unless otherwise specified. Analytical thin-layer chromatography was performed on silica gel $^{60}F_{254}$ aluminum-backed plates (MERCK MILLIPORE) and were visualized by fluorescence quenching under UV light or by $KMnO_4$ staining. Chromatography was performed with silica gel 60 (particle size 0.040 – 0.063 µm) using an automated purification system (ISCO TELEDYNE). NMR spectra were recorded on an Bruker Ascend-300 300 MHz at 298 K unless otherwise specified. Chemical shifts are reported in ppm on the δ scale and referenced to the appropriate solvent peak. DMSO-d_6, MeOD and $CDCl_3$ contain H_2O. HRMS analyses were carried out at the Monash University Mass Spectrometry Facility on an Agilent 6224 TOF LC/MS Mass Spectrometer coupled to an Agilent 1290 Infinity (Agilent, Palo Alto, CA). All data were acquired and reference mass corrected via a dual-spray electrospray ionisation (ESI) source. LCMS were recorded on an Agilent LCMS system composed of an Agilent G6120B Mass Detector, 1260 Infinity G1312B Binary pump, 1260 Infinity G1367E HiPALS autosampler and 1260 Infinity G4212B Diode Array Detector. Conditions for LCMS were as follows, (Method A) column:

Poroshell 120 EC-C18, 2.1 x 50 mm 2.7 Micron at 20 C, injection volume 2 μL, gradient: 5–100% B over 3 min (solvent A: water 0.1% formic acid; solvent B: acetonitrile 0.1% formic acid), flow rate: 0.8 mL/min, detection: 254 nm, acquisition time: 5 min. HPLC conditions used to assess purity of final compounds were as follows, column: Phenomenex Gemini C18, 2.0 x 50 mm; injection volume 20 μL; gradient: 0–100% Buffer B over 6 min (buffer A: 0.1% formic acid in autoclaved MilliQ water; buffer B: 0.1% formic acid in 100% acetonitrile), flow rate: 1.0 mL/min, detection: 214 or 224 nm.

dTag and HaloPROTAC synthesis

The previously described compounds dTag13 and dTag48[1], dTag^{V}-1[2], HaloPROTAC A and E [3, 4] were synthesized according to the literature and their analytical data obtained matched the reported.

NanoLuc PROTACs (NanoTACs)

Nanoluc inhibitors **1** and **2** for the synthesis of NanoLuc targeting PROTAC degraders (NanoTACs) were kindly provided by Joel R. Walker (PROMEGA) and their synthesis had been described earlier in the literature.[5]

The CRBN[1] and VHL[3] ligands used in the attachment of the E3 ligand part via varying linkers were synthesized as reported in the literature. The general procedure for the generation of NanoTAC compounds in summarised below **(Supplementary Figure 6).**

General procedure to obtain NanoTAC final compounds:

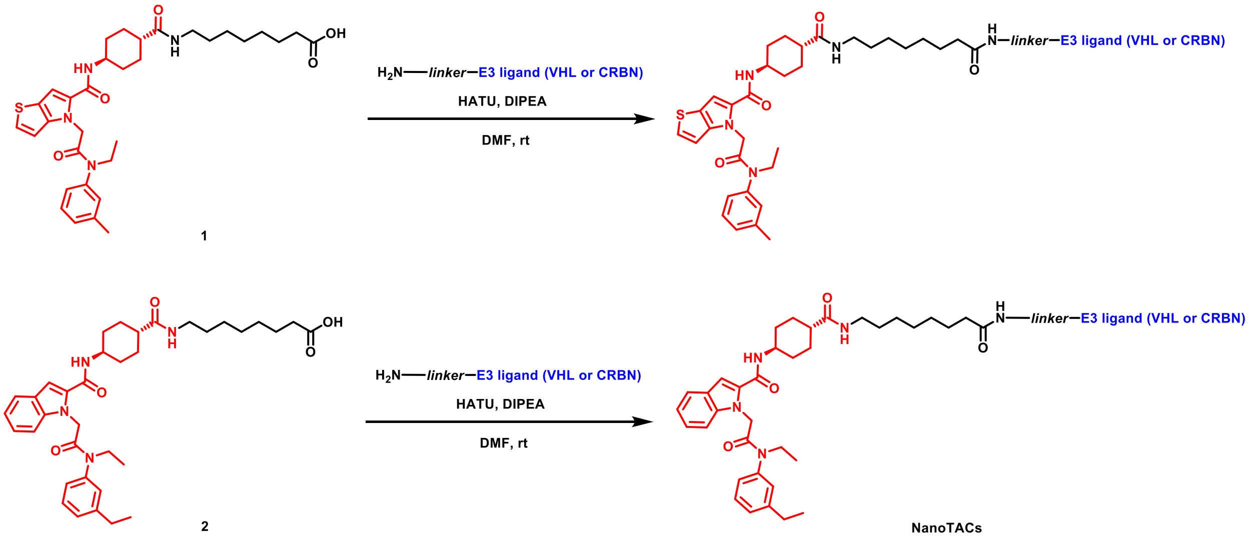

Supplementary Figure 6. General synthetic route to NanoLuc PROTAC (NanoTAC) degraders.

Terminal carboxylic acid group bearing NanoLuc inhibitors **1** or **2** (1.1 equiv) and HATU (1.1 equiv.) were dissolved in DMF (1 mL) and DIPEA (3 equiv.) was added. The reaction mixture was stirred for 5 min at ambient temperature until the solution was clear. A solution of the

respective benzylamine hydrochloride salt (1 equiv.) in DMF (0.5 mL) was then added and the resulting mixture was stirred for a minimum of 30 min at ambient temperature and reaction progress was monitored via HPLC-MS. Upon completion of the reaction, the solvent was removed in vacuum and the residue absorbed on silica gel. Purification via flash column chromatography (ISCO, eluting with gradient of 1 – 10% MeOH in CH_2Cl_2) afforded the final title compounds.

N-((1r,4r)-4-((8-((6-((2-(2,6-dioxopiperidin-3-yl)-1,3-dioxoisoindolin-4-yl)oxy)hexyl)amino)-8-oxooctyl)carbamoyl)cyclohexyl)-4-(2-(ethyl(m-tolyl)amino)-2-oxoethyl)-4H-thieno[3,2-b]pyrrole-5-carboxamide (NC1; WEHI-1655689)

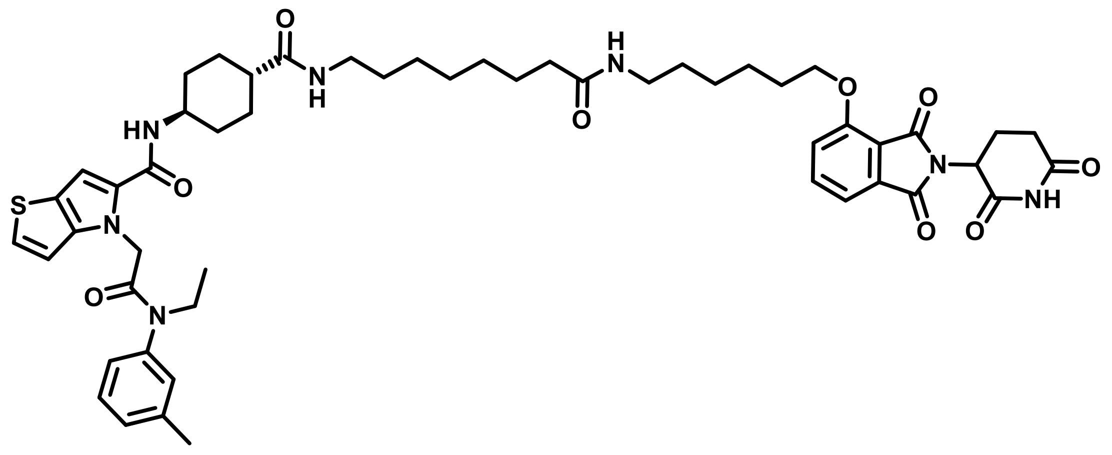

Following the general procedure, 8-[[4-[[4-[2-(N-ethyl-3-methyl-anilino)-2-oxo-ethyl]thieno[3,2-b]pyrrole-5-carbonyl]amino]cyclohexanecarbonyl]amino]octanoic acid **1** (15.0 mg, 0.0246 mmol), HATU (10.3 mg, 0.0271 mmol) and DIPEA (0.0129 mL, 0.0739 mmol) were dissolved in DMF (1 mL). The reaction mixture was stirred at ambient temperature for 5 min, before adding 6-[2-(2,6-dioxo-3-piperidyl)-1,3-dioxo-isoindolin-4-yl]oxyhexylammonium chloride (11.1 mg, 0.0271 mmol). The reaction mixture was stirred at ambient temperature for 16 h until HPLC-MS indicated consumption of starting material and product formation. The solvent was evaporated under a stream of nitrogen gas and the residue purified by flash column chromatography (ISCO, 12 g silica gel, 0 to 10% MeOH in CH_2Cl_2). The title compound was obtained as off-white sticky solid (22 mg, 93% yield).

1H NMR (300 MHz, Chloroform-d) δ 8.65 (s, 1H), 7.66 (dd, J = 8.5, 7.3 Hz, 1H), 7.42 (d, J = 7.2 Hz, 1H), 7.36 (t, J = 7.6 Hz, 1H), 7.24 – 7.10 (m, 4H), 6.79 (s, 1H), 6.74 (d, J = 5.3 Hz, 1H), 6.27 (d, J = 8.1 Hz, 1H), 5.96 (t, J = 5.8 Hz, 1H), 5.86 (t, J = 5.7 Hz, 1H), 4.97 (s, 2H), 4.91 (dd, J = 12.1, 5.1 Hz, 1H), 4.16 (t, J = 6.3 Hz, 2H), 3.93 – 3.77 (m, 0H), 3.71 (q, J = 7.2 Hz, 2H), 3.60 (p, J = 6.7 Hz, 2H), 3.28 – 3.16 (m, 4H), 3.09 (q, J = 7.4 Hz, 2H), 2.91 – 2.63 (m, 4H), 2.41 (s, 3H), 2.21 – 2.00 (m, 6H), 1.96 – 1.79 (m, 4H), 1.68 – 1.39 (m, 8H), 1.27 (dd, J = 10.3, 5.6 Hz, 10H), 1.10 (t, J = 7.1 Hz, 3H).

13C NMR (75 MHz, CDCl3) δ 175.78, 173.63, 171.33, 168.52, 167.45, 167.22, 165.98, 161.73, 156.77, 144.13, 140.71, 140.35, 136.76, 133.86, 131.14, 129.95, 129.48, 128.98, 125.52, 121.93, 119.18, 117.15, 115.86, 110.13, 103.77, 69.48, 55.38, 49.24, 47.89, 44.76, 44.64, 43.38, 39.49, 39.32, 36.70, 32.31, 31.49, 29.81, 29.52, 29.46, 28.99, 28.81, 28.67, 26.60, 26.50, 25.72, 25.64, 22.75, 21.47, 13.08, 12.59.

HRMS (TOFMS ES+, m/z) exact mass ([Chemical Formula: $C_{52}H_{65}N_7O_9S$]+H)+ 963.4564, found 963.4569; LCMS (Method A, UV, ES) RT = 2.71 min, [M+H]+ = 964.4.

N-((1r,4r)-4-((8-((4-((2-(2,6-dioxopiperidin-3-yl)-1,3-dioxoisoindolin-4-yl)amino)butyl)amino)-8-oxooctyl)carbamoyl)cyclohexyl)-4-(2-(ethyl(m-tolyl)amino)-2-oxoethyl)-4H-thieno[3,2-b]pyrrole-5-carboxamide (NC2; WEHI-1655698)

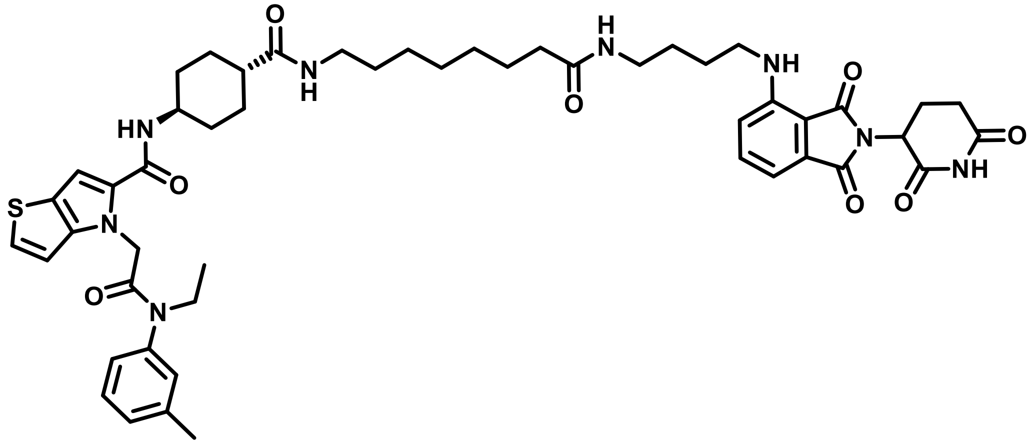

Following the general procedure, 8-[[4-[[4-[2-(N-ethyl-3-methyl-anilino)-2-oxo-ethyl]thieno[3,2-b]pyrrole-5-carbonyl]amino]cyclohexanecarbonyl]amino]octanoic acid **1** (15.0 mg, 0.0246 mmol), HATU (10.3 mg, 0.0271 mmol) and DIPEA (0.0129 mL, 0.0739 mmol) were dissolved in

DMF (1 mL). The reaction mixture was stirred at ambient temperature for 5 min, before adding 4-[[2-(2,6-dioxo-3-piperidyl)-1,3-dioxo-isoindolin-4-yl]amino]butylammonium chloride (10.3 mg, 0.0271 mmol). The reaction mixture was stirred at ambient temperature for 16 h until HPLC-MS indicated consumption of starting material and product formation. The solvent was evaporated under a stream of nitrogen gas and the residue purified by flash column chromatography (ISCO, 12 g silica gel, 0 to 10% MeOH in CH_2Cl_2). The title compound was obtained as off-white sticky solid (21 mg, 91% yield).

1H NMR (300 MHz, Chloroform-d) δ 8.65 (s, 1H), 7.47 (dd, J = 8.5, 7.1 Hz, 1H), 7.36 (t, J = 7.6 Hz, 1H), 7.24 – 7.10 (m, 4H), 7.06 (d, J = 7.0 Hz, 1H), 6.89 (d, J = 8.6 Hz, 1H), 6.80 (s, 1H), 6.74 (d, J = 5.4 Hz, 1H), 6.28 (d, J = 8.0 Hz, 1H), 6.22 (t, J = 5.7 Hz, 1H), 6.09 (t, J = 5.9 Hz, 1H), 5.89 (t, J = 5.6 Hz, 1H), 4.97 (s, 2H), 4.88 (dd, J = 12.0, 5.2 Hz, 1H), 3.92 – 3.76 (m, 1H), 3.71 (q, J = 7.1 Hz, 2H), 3.59 (p, J = 6.6 Hz, 2H), 3.35 – 3.23 (m, 4H), 3.23 – 3.14 (m, 2H), 3.08 (q, J = 7.4 Hz, 2H), 2.86 – 2.58 (m, 3H), 2.41 (s, 3H), 2.20 – 2.00 (m, 4H), 1.96 – 1.82 (m, 2H), 1.71 – 1.53 (m, 8H), 1.42 (d, J = 18.1 Hz, 2H), 1.27 (dd, J = 9.1, 4.4 Hz, 8H), 1.10 (t, J = 7.1 Hz, 3H).

13C NMR (75 MHz, CDCl3) δ 175.65, 173.60, 171.31, 169.55, 168.73, 167.66, 167.35, 161.58, 146.89, 144.02, 140.58, 140.24, 136.24, 132.44, 131.03, 129.84, 129.38, 128.85, 127.19, 125.39, 121.81, 116.80, 111.47, 110.04, 109.87, 103.66, 55.31, 48.91, 47.77, 44.65, 44.49, 43.30, 42.21, 39.17, 38.91, 36.54, 32.18, 31.39, 29.35, 28.86, 28.57, 27.05, 26.59, 26.36, 25.50, 22.78, 21.36, 12.97, 12.49.

HRMS (TOFMS ES+, m/z) exact mass ([Chemical Formula: $C_{50}H_{62}N_8O_8S$]+H)+ 934.4411, found 934.4406; LCMS (Method A, UV, ES) RT = 2.66 min, [M+H]+ = 935.4.

N-((1r,4r)-4-((8-((6-((2-(2,6-dioxopiperidin-3-yl)-1,3-dioxoisoindolin-4-yl)oxy)hexyl)amino)-8-oxooctyl)carbamoyl)cyclohexyl)-1-(2-(ethyl(3-ethylphenyl)amino)-2-oxoethyl)-1H-indole-2-carboxamide (NC3; WEHI-1880458)

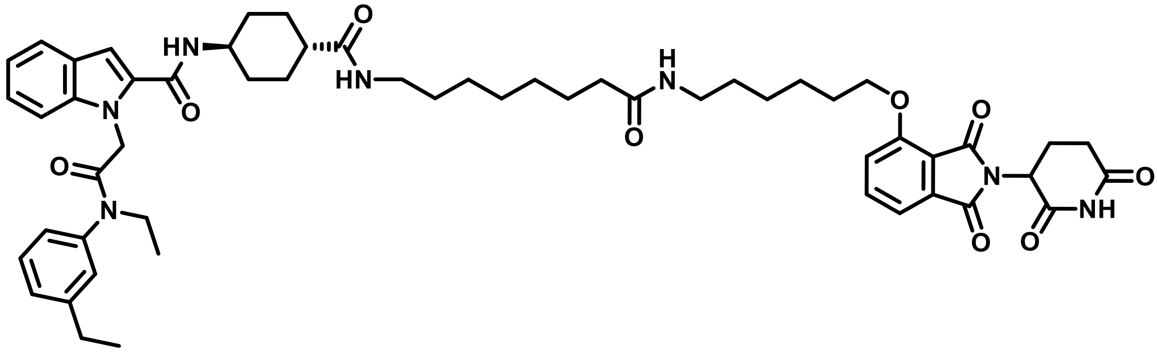

Following the general procedure, 8-[[4-[[1-[2-(N,3-diethylanilino)-2-oxo-ethyl]indole-2-carbonyl]amino]cyclohexanecarbonyl]amino]octanoic acid **2** (17 mg, 0.027 mmol), HATU (10 mg, 0.027 mmol) and DIPEA (0.0085 mL, 0.049 mmol) were dissolved in DMF (1 mL). The reaction mixture was stirred at ambient temperature for 5 min, before 6-[2-(2,6-dioxo-3-piperidyl)-1,3-dioxo-isoindolin-4-yl]oxyhexylammonium chloride (10 mg, 0.024 mmol) was added. The reaction mixture was stirred at ambient temperature for 16 h until HPLC-MS indicated consumption of starting material and product formation. The solvent was evaporated under a stream of nitrogen gas and the residue purified by flash column chromatography (ISCO, 12 g silica gel, 0 to 10% MeOH in CH2Cl2). The title compound was obtained as off-white sticky solid (13 mg, 55% yield).

1H NMR (300 MHz, Chloroform-d) δ 8.44 (s, 1H), 7.66 (dd, J = 8.5, 7.3 Hz, 1H), 7.58 (d, J = 7.6 Hz, 1H), 7.48 – 7.39 (m, 2H), 7.23 – 7.19 (m, 3H), 7.09 (t, J = 7.5 Hz, 2H), 6.82 (s, 1H), 6.43 (d, J = 8.0 Hz, 1H), 5.69 (t, J = 5.6 Hz, 1H), 5.59 (t, J = 5.6 Hz, 1H), 5.02 (s, 2H), 4.89 (dd, J = 12.2, 5.4 Hz, 1H), 4.18 (t, J = 6.0 Hz, 1H), 3.97 – 3.83 (m, 1H), 3.73 (q, J = 7.1 Hz, 2H), 3.24 (p, J = 6.6 Hz, 4H), 2.86 – 2.53 (m, 5H), 2.24 – 1.83 (m, 10H), 1.68 – 1.38 (m, 11H), 1.34 – 1.23 (m, 11H), 1.11 (t, J = 7.1 Hz, 3H).

13C NMR (75 MHz, CDCl3) δ 175.36, 173.25, 171.02, 168.32, 167.54, 167.20, 165.96, 156.80, 146.80, 141.08, 138.79, 136.71, 133.97, 133.30, 130.10, 128.29, 127.90, 126.50, 125.78, 124.06, 122.03, 120.69, 119.14, 117.30, 115.94, 109.63, 104.48, 69.54, 49.28, 48.08, 46.57, 44.84, 44.70,

39.46, 39.30, 36.79, 32.43, 31.49, 29.67, 29.61, 29.05, 28.86, 28.81, 28.75, 28.67, 26.65, 26.53, 25.79, 25.59, 22.76, 15.63, 13.17.

HRMS (TOFMS ES+, m/z) exact mass ([Chemical Formula: $C_{55}H_{69}N_7O_9$]+H)+ 971.5157, found 971.5151; LCMS (Method A, UV, ES) RT = 2.93 min, [M+H]+ = 972.4.

N-((1r,4r)-4-((8-((4-((2-(2,6-dioxopiperidin-3-yl)-1,3-dioxoisoindolin-4-yl)amino)butyl)amino)-8-oxooctyl)carbamoyl)cyclohexyl)-1-(2-(ethyl(3-ethylphenyl)amino)-2-oxoethyl)-1H-indole-2-carboxamide (NC4; WEHI-1880459)

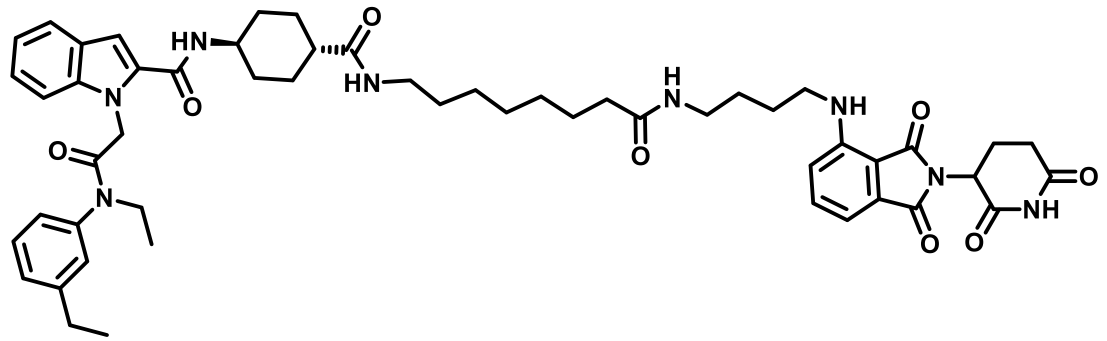

Following the general procedure, [[4-[[1-[2-(N,3-diethylanilino)-2-oxo-ethyl]indole-2-carbonyl]amino]cyclohexanecarbonyl]amino]octanoic acid **2** (17 mg, 0.027 mmol), HATU (10 mg, 0.027 mmol) and DIPEA (0.00850 mL, 0.0488 mmol) were dissolved in DMF (1 mL). The reaction mixture was stirred at ambient temperature for 5 min, before 6-[2-(2,6-dioxo-3-piperidyl)-1,3-dioxo-isoindolin-4-yl]oxyhexylammonium chloride (10.0 mg, 0.0244 mmol). The reaction mixture was stirred at ambient temperature for 16 h until HPLC-MS indicated consumption of starting material and product formation. The solvent was evaporated under a stream of nitrogen gas and the residue purified by flash column chromatography (ISCO, 12 g silica gel, 0 to 10% MeOH in CH_2Cl_2). The title compound was obtained as off-white sticky solid (15 mg, 65% yield).

1H NMR (300 MHz, Chloroform-d) δ 8.48 (s, 1H), 7.62 – 7.54 (m, 1H), 7.49 (dd, J = 8.5, 7.1 Hz, 1H), 7.43 (t, J = 8.1 Hz, 1H), 7.23 – 7.18 (m, 2H), 7.13 – 7.04 (m, 3H), 6.89 (d, J = 8.6 Hz, 1H), 6.83 (s, 1H), 6.46 (d, J = 8.0 Hz, 1H), 6.23 (t, J = 5.7 Hz, 1H), 5.82 (t, J = 5.9 Hz, 1H), 5.63 (t, J = 5.7 Hz, 1H), 5.02 (s, 2H), 4.85 (dd, J = 12.2, 5.2 Hz, 1H), 3.97 – 3.82 (m, 1H), 3.73 (q, J = 7.1 Hz, 2H), 3.35 – 3.16 (m, 6H), 2.82 – 2.50 (m, 5H), 2.15 (t, J = 7.5 Hz, 4H), 2.11 – 1.87 (m, 4H), 1.73 – 1.54 (m, 10H), 1.47 (t, J = 6.8 Hz, 2H), 1.39 – 1.20 (m, 11H), 1.11 (t, J = 7.1 Hz, 3H).

13C NMR (75 MHz, CDCl3) δ 175.38, 173.42, 171.05, 169.73, 168.59, 167.74, 167.56, 162.33, 147.03, 146.80, 141.06, 138.79, 136.36, 133.31, 132.64, 130.12, 128.31, 127.89, 126.49, 125.77, 124.08, 122.02, 120.71, 120.71, 118.95, 116.84, 111.73, 109.64, 104.50, 49.05, 48.06, 44.84, 44.71, 42.35, 39.23, 39.02, 36.73, 32.42, 31.48, 29.59, 28.98, 28.81, 28.66, 28.61, 27.37, 26.74, 26.43, 25.51, 22.91, 15.63, 13.17.

HRMS (TOFMS ES+, m/z) exact mass ([Chemical Formula: $C_{53}H_{66}N_8O_8$]+H)+ 942.5004, found 942.5003; LCMS (Method A, UV, ES) RT = 2.87 min, [M+H]+ = 943.4.

N-((1r,4r)-4-((8-((3-((2-(2,6-dioxopiperidin-3-yl)-1,3-dioxoisoindolin-4-yl)amino)propyl)amino)-8-oxooctyl)carbamoyl)cyclohexyl)-1-(2-(ethyl(3-ethylphenyl)amino)-2-oxoethyl)-1H-indole-2-carboxamide (NC5; WEHI-1880797)

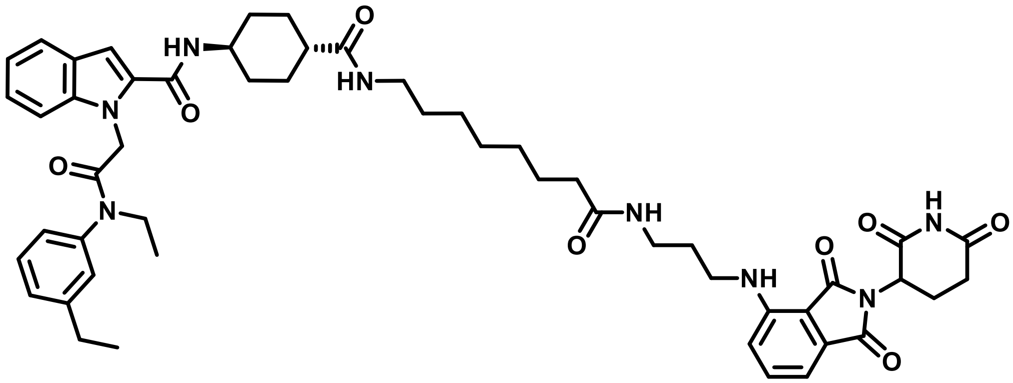

Following the general procedure, 8-[[4-[[1-[2-(N,3-diethylanilino)-2-oxo-ethyl]indole-2-carbonyl]amino]cyclohexanecarbonyl]amino]octanoic acid **2** (17 mg, 0.027 mmol), HATU (10 mg, 0.027 mmol) and DIPEA (0.0142 mL, 0.0818 mmol) were dissolved in DMF (1 mL). The reaction mixture was stirred at ambient temperature for 5 min, before 3-[[2-(2,6-dioxo-3-piperidyl)-1,3-dioxo-isoindolin-4-yl]amino]propylammonium chloride (10 mg, 0.027 mmol) was added. The reaction mixture was stirred at ambient temperature for 16 h until HPLC-MS indicated consumption of starting material and product formation. The solvent was evaporated under a stream of nitrogen gas and the residue purified by flash column chromatography (ISCO, 12 g silica gel, 0 to 10% MeOH in CH2Cl2). The title compound was obtained as off-white sticky solid (11.0 mg, 43% yield).

1H NMR (300 MHz, Chloroform-d) δ 8.74 (s, 1H), 7.57 (d, J = 7.6 Hz, 1H), 7.47 (dd, J = 8.5, 7.1 Hz, 1H), 7.41 (t, J = 8.0 Hz, 1H), 7.23 – 7.19 (m, 2H), 7.13 – 7.03 (m, 3H), 6.92 – 6.82 (m, 2H), 6.61 (d, J = 8.0 Hz, 1H), 6.37 – 6.27 (m, 1H), 5.97 – 5.81 (m, 1H), 5.01 (s, 2H), 4.84 (dd, J = 12.3, 5.3 Hz, 1H), 3.95 – 3.80 (m, 1H), 3.73 (q, J = 7.2 Hz, 2H), 3.32 (p, J = 6.4 Hz, 4H), 3.21 (q, J = 6.6 Hz, 2H), 2.82 – 2.47 (m, 5H), 2.22 – 1.76 (m, 13H), 1.69 – 1.38 (m, 6H), 1.39 – 1.17 (m, 10H), 1.11 (t, J = 7.1 Hz, 3H).

13C NMR (75 MHz, CDCl3) δ 175.80, 174.24, 171.35, 169.62, 168.85, 167.78, 167.69, 162.33, 146.83, 140.97, 138.76, 136.33, 133.19, 132.69, 130.12, 128.34, 127.85, 126.45, 125.74, 124.10, 122.01, 120.72, 116.76, 111.67, 110.26, 109.65, 104.69, 49.07, 48.04, 46.56, 44.73, 44.63, 40.19, 39.20, 37.12, 36.41, 32.24, 31.45, 29.49, 28.90, 28.79, 28.62, 28.45, 26.30, 25.51, 22.83, 15.62, 13.14.

HRMS (TOFMS ES+, m/z) exact mass ([Chemical Formula: $C_{52}H_{64}N_8O_8$]+H)+ 928.4847, found 928.4845; LCMS (Method A, UV, ES) RT = 3.08 min, [M+H]+ = 929.4

1-(2-(ethyl(3-ethylphenyl)amino)-2-oxoethyl)-N-((1r,4r)-4-((8-((4-((2-(1-methyl-2,6-dioxopiperidin-3-yl)-1,3-dioxoisoindolin-4-yl)amino)butyl)amino)-8-oxooctyl)carbamoyl)cyclohexyl)-1H-indole-2-carboxamide (NC*; WEHI-1880796)

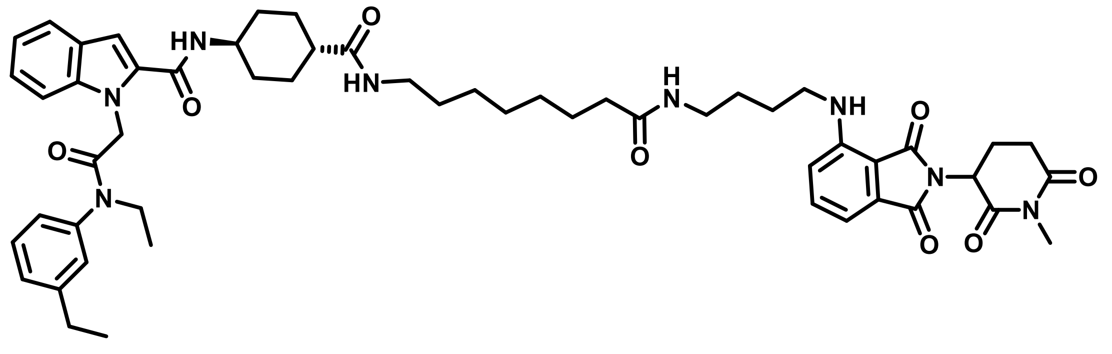

Following the general procedure, 8-[[4-[[1-[2-(N,3-diethylanilino)-2-oxo-ethyl]indole-2-carbonyl]amino]cyclohexanecarbonyl]amino]octanoic acid **2** (27 mg, 0.043 mmol), HATU (16 mg, 0.043 mmol) and DIPEA (0.0225 mL, 0.129 mmol) were dissolved in DMF (1 mL). The reaction mixture was stirred at ambient temperature for 5 min, before 4-[[2-(1-methyl-2,6-dioxo-3-piperidyl)-1,3-dioxo-isoindolin-4-yl]amino]butylammonium chloride (17.0 mg, 0.0431 mmol) was added. The reaction mixture was stirred at ambient temperature for 16 h until HPLC-MS

indicated consumption of starting material and product formation. The solvent was evaporated under a stream of nitrogen gas and the residue purified by flash column chromatography (ISCO, 12 g silica gel, 0 to 10% MeOH in CH2Cl2). The title compound was obtained as off-white sticky solid (23 mg, 56% yield).

1H NMR (300 MHz, Chloroform-d) δ 7.58 (d, J = 7.4 Hz, 1H), 7.52 – 7.38 (m, 2H), 7.23 – 7.17 (m, 2H), 7.13 – 7.05 (m, 3H), 6.89 (d, J = 8.6 Hz, 1H), 6.83 (s, 1H), 6.35 (d, J = 8.0 Hz, 1H), 6.23 (t, J = 5.8 Hz, 1H), 5.69 (t, J = 6.0 Hz, 1H), 5.56 (t, J = 5.8 Hz, 1H), 5.03 (s, 2H), 4.93 – 4.83 (m, 1H), 3.98 – 3.83 (m, 1H), 3.73 (q, J = 7.2 Hz, 2H), 3.30 (q, J = 6.3 Hz, 4H), 3.24 – 3.17 (m, 5H), 2.99 – 2.88 (m, 1H), 2.80 – 2.66 (m, 4H), 2.23 – 2.12 (m, 4H), 2.10 – 1.90 (m, 4H), 1.73 – 1.55 (m, 11H), 1.52 – 1.42 (m, 2H), 1.37 – 1.25 (m, 10H), 1.12 (t, J = 7.1 Hz, 3H).

13C NMR (75 MHz, CDCl3) δ 175.29, 173.36, 171.38, 169.85, 169.25, 167.87, 167.47, 162.15, 146.97, 146.79, 141.06, 138.86, 136.31, 133.11, 132.66, 130.10, 128.29, 127.89, 126.46, 125.76, 124.14, 122.06, 120.73, 116.76, 111.67, 110.20, 109.60, 104.48, 49.78, 48.06, 46.64, 44.81, 44.66, 42.33, 39.29, 39.02, 36.74, 32.47, 32.02, 29.65, 29.06, 28.80, 28.74, 28.66, 27.40, 27.36, 26.74, 26.54, 25.59, 22.28, 15.63, 13.18.

HRMS (TOFMS ES+, m/z) exact mass ([Chemical Formula: $C_{54}H_{68}N_8O_8$]+H)+ 956.5160, found 956.5167; LCMS (Method A, UV, ES) RT = 3.25 min, [M+H]+ = 957.4

N-((1r,4r)-4-((8-((6-(2-(((2S,4R)-1-((S)-2-(1-cyanocyclopropanecarboxamido)-3,3-dimethylbutanoyl)-4-hydroxypyrrolidine-2-carboxamido)methyl)-5-(4-methylthiazol-5-yl)phenoxy)hexyl)amino)-8-oxooctyl)carbamoyl)cyclohexyl)-1-(2-(ethyl(3-ethylphenyl)amino)-2-oxoethyl)-1H-indole-2-carboxamide (NV1; WEHI-1880456)

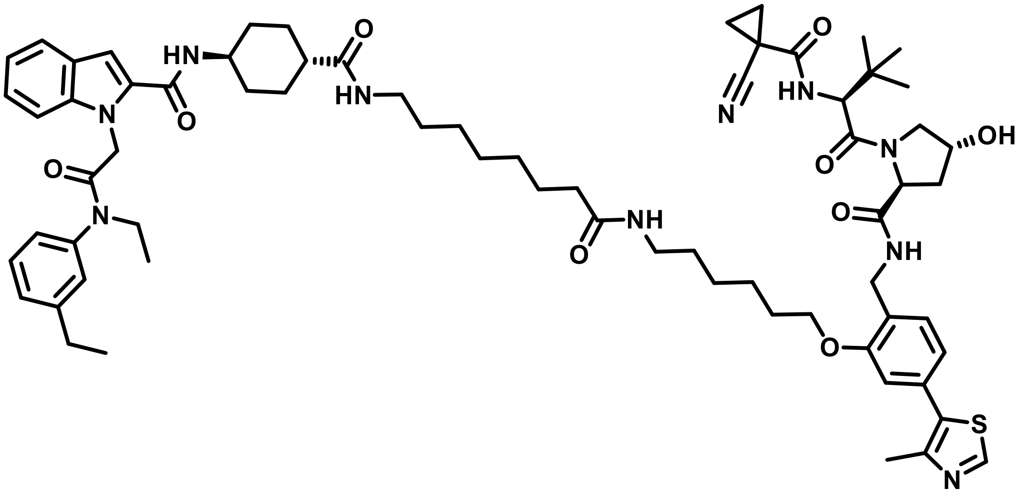

Following the general procedure, 8-[[4-[[1-[2-(N,3-diethylanilino)-2-oxo-ethyl]indole-2-carbonyl]amino]cyclohexanecarbonyl]amino]octanoic acid **2** (12 mg, 0.019 mmol), HATU (8.1 mg, 0.021 mmol) and DIPEA (0.0101 mL, 0.0578 mmol) were dissolved in DMF (1 mL). The reaction mixture was stirred at ambient temperature for 5 min, before 6-[2-[[[(2S,4R)-1-[(2S)-2-[(1-cyanocyclopropanecarbonyl)amino]-3,3-dimethyl-butanoyl]-4-hydroxy-pyrrolidine-2-carbonyl]amino]methyl]-5-(4-methylthiazol-5-yl)phenoxy]hexylammonium chloride (14 mg, 0.021 mmol) was added. The reaction mixture was stirred at ambient temperature for 16 h until HPLC-MS indicated consumption of starting material and product formation. The solvent was evaporated under a stream of nitrogen gas and the residue purified by flash column chromatography (ISCO, 12 g silica gel, 0 to 10% MeOH in CH_2Cl_2). The title compound was obtained as off-white sticky solid (16 mg, 67% yield).

1H NMR (300 MHz, Chloroform-d) δ 8.67 (s, 1H), 7.56 (d, J = 7.5 Hz, 1H), 7.43 (t, J = 8.0 Hz, 1H), 7.36 – 7.27 (m, 3H), 7.20 (dd, J = 6.6, 1.4 Hz, 2H), 7.13 – 7.01 (m, 3H), 6.94 (dd, J = 7.7, 1.6 Hz, 1H), 6.86 (d, J = 1.6 Hz, 1H), 6.80 (s, 1H), 6.62 (d, J = 8.0 Hz, 1H), 5.93 (t, J = 5.8 Hz, 1H), 5.66 (t, J = 5.8 Hz, 1H), 5.18 – 4.93 (m, 2H), 4.64 (t, J = 7.6 Hz, 1H), 4.53 – 4.42 (m, 4H), 4.02 (t, J = 6.0 Hz, 2H), 3.96 – 3.85 (m, 1H), 3.79 (d, J = 11.0 Hz, 1H), 3.69 (q, J = 7.1 Hz, 2H), 3.59 (dd, J = 11.0, 4.2 Hz, 1H), 3.38 (d, J = 5.0 Hz, 1H), 3.31 – 3.17 (m, 4H), 2.74 (d, J = 7.6 Hz, 2H), 2.52

(s, 3H), 2.50 – 2.42 (m, 1H), 2.24 – 1.80 (m, 10H), 1.73 – 1.38 (m, 17H), 1.37 – 1.22 (m, 12H), 1.08 (t, J = 7.1 Hz, 3H), 0.91 (s, 8H).

HRMS (TOFMS ES+, m/z) exact mass ([Chemical Formula: $C_{69}H_{92}N_{10}O_9S$]+H)+ 1236.6769, found 1236.6772; LCMS (Method A, UV, ES) RT = 3.10 min, [M+H]+ = 1234.6

Note: The amine-linked VHL ligand (2S,4R)-N-(2-((16-amino-2-oxo-7,10,13-trioxa-3-azahexadecyl)oxy)-4-(4-methylthiazol-5-yl)benzyl)-1-((S)-2-(1-cyanocyclopropanecarboxamido)-3,3-dimethylbutanoyl)-4-hydroxypyrrolidine-2-carboxamide was synthesized according to literature reports[3].

1-(2-(ethyl(3-ethylphenyl)amino)-2-oxoethyl)-N-((1r,4r)-4-((8-((7-(((S)-1-((2S,4R)-4-hydroxy-2-((4-(4-methylthiazol-5-yl)benzyl)carbamoyl)pyrrolidin-1-yl)-3,3-dimethyl-1-oxobutan-2-yl)amino)-7-oxoheptyl)amino)-8-oxooctyl)carbamoyl)cyclohexyl)-1H-indole-2-carboxamide (NV2; WEHI-1881306)

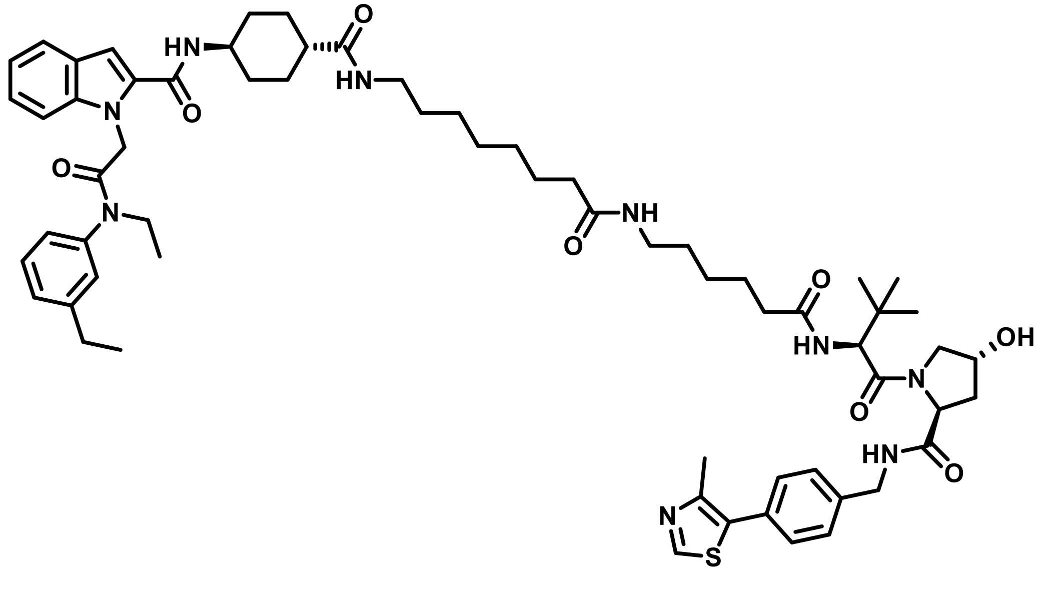

Following the general procedure, 8-[[4-[[1-[2-(N,3-diethylanilino)-2-oxo-ethyl]indole-2-carbonyl]amino]cyclohexanecarbonyl]amino]octanoic acid **2** (5.0 mg, 0.0081 mmol), HATU (3.7 mg, 0.0097 mmol) and DIPEA (0.00424 mL, 0.0243 mmol) were dissolved in DMF (1 mL). The reaction mixture was stirred at ambient temperature for 5 min, before [7-[[(1S)-1-[(2S,4R)-4-

hydroxy-2-[[4-(4-methylthiazol-5-yl)phenyl]methylcarbamoyl]pyrrolidine-1-carbonyl]-2,2-dimethyl-propyl]amino]-7-oxo-heptyl]ammonium chloride (5.07 mg, 0.00811 mmol) was added. The reaction mixture was stirred at ambient temperature for 16 h until HPLC-MS indicated consumption of starting material and product formation. The solvent was evaporated under a stream of nitrogen gas and the residue purified by flash column chromatography (ISCO, 12 g silica gel, 0 to 10% MeOH in CH2Cl2). The title compound was obtained as off-white sticky solid (4.0 mg, 43% yield).

1H NMR (300 MHz, Chloroform-d) δ 8.67 (s, 1H), 7.61 – 7.56 (m, 1H), 7.43 (t, J = 8.0 Hz, 1H), 7.38 – 7.27 (m, 5H), 7.24 – 7.18 (m, 2H), 7.09 (dd, J = 7.7, 6.4 Hz, 2H), 6.86 (s, 1H), 6.60 (d, J = 8.0 Hz, 1H), 6.24 (d, J = 8.8 Hz, 1H), 5.74 (dt, J = 10.7, 5.6 Hz, 2H), 5.04 (d, J = 4.0 Hz, 2H), 4.66 (t, J = 8.0 Hz, 1H), 4.58 – 4.41 (m, 3H), 4.33 (dd, J = 15.0, 5.4 Hz, 1H), 4.01 – 3.85 (m, 2H), 3.72 (q, J = 7.2 Hz, 3H), 3.53 (dd, J = 11.3, 3.6 Hz, 1H), 3.22 (p, J = 6.8 Hz, 4H), 2.74 (q, J = 7.6 Hz, 2H), 2.51 (s, 3H), 2.48 – 2.35 (m, 1H), 2.30 – 2.02 (m, 8H), 1.96 (d, J = 13.3 Hz, 2H), 1.74 – 1.55 (m, 9H), 1.54 – 1.41 (m, 4H), 1.39 – 1.21 (m, 15H), 1.11 (t, J = 7.1 Hz, 3H), 0.94 (s, 8H).

13C NMR (75 MHz, CDCl3) δ 175.48, 173.67, 173.48, 171.89, 171.07, 167.64, 162.25, 150.42, 148.61, 146.83, 140.99, 138.80, 138.34, 133.19, 131.78, 131.04, 130.14, 129.63, 128.35, 128.21, 127.85, 126.48, 125.74, 124.11, 122.13, 120.71, 109.54, 104.62, 70.09, 58.71, 57.57, 56.91, 48.04, 46.61, 44.85, 44.71, 43.32, 39.29, 39.21, 36.69, 36.43, 36.25, 35.27, 32.42, 29.55, 29.36, 28.85, 28.81, 28.70, 28.56, 28.46, 26.58, 26.39, 26.38, 25.47, 16.22, 15.63, 13.16.

HRMS (TOFMS ES+, m/z) exact mass ([Chemical Formula: $C_{65}H_{89}N_9O_8S$]+H)+ 1155.6555, found 1155.6565; LCMS (Method A, UV, ES) RT = 2.49 min, [M+H]+ = 1156.4

SUPPLEMENTARY REFERENCES

1. Nabet B*, et al.* The dTAG system for immediate and target-specific protein degradation. *Nat Chem Biol* **14**, 431-441 (2018).

2. Nabet B*, et al.* Rapid and direct control of target protein levels with VHL-recruiting dTAG molecules. *Nat Commun* **11**, 4687 (2020).

3. Tovell H*, et al.* Rapid and Reversible Knockdown of Endogenously Tagged Endosomal Proteins via an Optimized HaloPROTAC Degrader. *ACS Chem Biol* **14**, 882-892 (2019).

4. Buckley DL*, et al.* HaloPROTACS: Use of Small Molecule PROTACs to Induce Degradation of HaloTag Fusion Proteins. *ACS Chem Biol* **10**, 1831-1837 (2015).

5. Walker JR*, et al.* Highly Potent Cell-Permeable and Impermeable NanoLuc Luciferase Inhibitors. *ACS Chem Biol* **12**, 1028-1037 (2017).
