# Supplementary material for: Development of NanoLuc-targeting protein degraders and a universal reporter system to benchmark tag-targeted degradation platforms
Source: Nat Commun. 2022 Apr 19;13:2073. doi: 10.1038/s41467-022-29670-1 (PMC9019100; doi:10.1038/s41467-022-29670-1)
Supplement: Supplementary file 2 — Reporting Summary [file 41467_2022_29670_MOESM2_ESM.pdf]

nature portfolio

Corresponding author(s): Dr. Rebecca Feltham

Last updated by author(s): 17/3/22

Reporting Summary

Nature Portfolio wishes to improve the reproducibility of the work that we publish. This form provides structure for consistency and transparency in reporting. For further information on Nature Portfolio policies, see our Editorial Policies and the Editorial Policy Checklist.

Statistics

For all statistical analyses, confirm that the following items are present in the figure legend, table legend, main text, or Methods section.

n/a	Confirmed	
☐	☒	The exact sample size (*n*) for each experimental group/condition, given as a discrete number and unit of measurement
☐	☒	A statement on whether measurements were taken from distinct samples or whether the same sample was measured repeatedly
☐	☒	The statistical test(s) used AND whether they are one- or two-sided<br>*Only common tests should be described solely by name; describe more complex techniques in the Methods section.*
☐	☒	A description of all covariates tested
☐	☒	A description of any assumptions or corrections, such as tests of normality and adjustment for multiple comparisons
☐	☒	A full description of the statistical parameters including central tendency (e.g. means) or other basic estimates (e.g. regression coefficient) AND variation (e.g. standard deviation) or associated estimates of uncertainty (e.g. confidence intervals)
☒	☐	For null hypothesis testing, the test statistic (e.g. *F*, *t*, *r*) with confidence intervals, effect sizes, degrees of freedom and *P* value noted<br>*Give P values as exact values whenever suitable.*
☒	☐	For Bayesian analysis, information on the choice of priors and Markov chain Monte Carlo settings
☒	☐	For hierarchical and complex designs, identification of the appropriate level for tests and full reporting of outcomes
☒	☐	Estimates of effect sizes (e.g. Cohen's *d*, Pearson's *r*), indicating how they were calculated

Our web collection on statistics for biologists contains articles on many of the points above.

Software and code

Policy information about availability of computer code

Data collection	IncuCyte S3 System<br>CLARIOstar Plus Plate Reader<br>ChemiDoc MP Imagine system<br>LSR II flow cytometer (Becton Dickinson, NJ)<br>Acuity M-class UPLC (Waters)<br>timsTOF pro II (Bruker)
Data analysis	Prism version 9<br>Weasel Version version 2.7<br>Incucyte Software v2019B<br>Image Lab version 6.1<br>MSfragger (v3.1) - NB: MaxLFQ is an algorithm used within this program<br>Fragpipe framework (v17.0)<br>limma package 41<br>BD FACSDiva v9.0

For manuscripts utilizing custom algorithms or software that are central to the research but not yet described in published literature, software must be made available to editors and reviewers. We strongly encourage code deposition in a community repository (e.g. GitHub). See the Nature Portfolio guidelines for submitting code & software for further information.

Data

Policy information about availability of data

All manuscripts must include a data availability statement. This statement should provide the following information, where applicable:

- Accession codes, unique identifiers, or web links for publicly available datasets
- A description of any restrictions on data availability
- For clinical datasets or third party data, please ensure that the statement adheres to our policy

Source data are provided with this paper. Reagents are available upon request. All NanoTACs are available upon request. Uncropped western blots are provided in the source data file. The mass spectrometry proteomics data generated in this study have been deposited in the ProteomeXchange Consortium via the PRIDE42 partner repository with the dataset identifier PXD031371. [https://www.ebi.ac.uk/pride/archive/projects/PXD031371/private]. Access can be obtained via login details: Username: reviewer_pxd031371@ebi.ac.uk Password: JIzUqDN8. Proteins were identified using the Uniprot Homo Sapiens Database (https://www.uniprot.org/uniprot/?query=proteome:UP000005640). Further information on research design is available in the Nature Research Reporting Summary linked to this article.

Field-specific reporting

Please select the one below that is the best fit for your research. If you are not sure, read the appropriate sections before making your selection.

☒ Life sciences ☐ Behavioural & social sciences ☐ Ecological, evolutionary & environmental sciences

For a reference copy of the document with all sections, see nature.com/documents/nr-reporting-summary-flat.pdf

Life sciences study design

All studies must disclose on these points even when the disclosure is negative.

Sample size	For in vitro assays no statistical analysis was used to determine sample size, but each experiment was performed in triplicate or quadruplicate technical repeats and repeated a minimum of 3 times independently to verify the results. Given the differing protein expression levels between experiments, samples sizes were taken based on consistent trends across biological replicates which were clear within the replicates performed.
Data exclusions	No data was excluded from the analyses.
Replication	All attempts to replicate experimental data were successful, where possible each individual experimental data point is indicated. Western blots: 3 independent replicates. Luminescence assays: 3 independent replicates with 4 technical replicate wells per experiment. Flow cytometry analysis: 3 independent replicates.
Randomization	For all experiments other than those mentioned here randomisation is always employed. In vitro experimental samples to treatment groups were designated at random using numerical identifiers.
Blinding	Blinding was not necessary as all samples were analysed simultaneously using identical assay conditions and any analysis performed was not through subjective scoring methods.

Reporting for specific materials, systems and methods

We require information from authors about some types of materials, experimental systems and methods used in many studies. Here, indicate whether each material, system or method listed is relevant to your study. If you are not sure if a list item applies to your research, read the appropriate section before selecting a response.

Materials & experimental systems

n/a	Involved in the study	
☐	☒	Antibodies
☐	☒	Eukaryotic cell lines
☒	☐	Palaeontology and archaeology
☐	☒	Animals and other organisms
☒	☐	Human research participants
☒	☐	Clinical data
☒	☐	Dual use research of concern

Methods

n/a	Involved in the study	
☒	☐	ChIP-seq
☐	☒	Flow cytometry
☒	☐	MRI-based neuroimaging

Antibodies

Antibodies used	Primary antibodies:

Antibodies used

HaloTag (Promega; Cat#: G9211; Lot#: 386205), NanoLuc (Promega; Cat#: N7000; Lot#: 442302), beta-actin (Santa Cruz; Cat#: sc-47778; Clone 4; Lot#: G031940), MLKL (produced in house; 3H1 clone), FKBP (R&D systems; Cat#: MAB3777; Clone 422513; Lot#: CBLL0118121), caspase-8 (Proteintech; Cat#: 13423-1-AP), Histone H3 (Abcam; Cat#: ab10799), Cadherin (Cell Signalling Technologies; Cat#: 4068T; Lot#: 2).

Secondary antibodies:
Peroxidase-AffiniPure Goat anti-Mouse IgG H+L (Jackson ImmunoResearch Labs; Cat#: 115-035-003), Peroxidase-AffiniPure Goat anti-Rabbit IgG H+L (Jackson ImmunoResearch Labs; Cat#: 111-035-003), Peroxidase-AffiniPure Goat anti-Rat IgG H+L (Jackson ImmunoResearch Labs; Cat#: 112-035-003).

Validation

HaloTag and NanoLuc antibodies were validated using cDNA encoding the HaloTag or NanoLuc with inducible expression systems and appropriate molecular weight markers to ensure that each antibody was on target. When expressed in HEK293T and MDF cells, the appropriate sized bands were only observed upon HaloTag- or NanoLuc-tagged protein induction.

From manufacturers website:
HaloTag: Little to no cross-reactivity with other non-HaloTag proteins.
NanoLuc: Mobility approximated the expected mobility for each (NanoLuc) fusion protein. Background bands are evident in all lanes, including HEK293 cell lysate, which lacks expression of a NanoLuc fusion protein, but the NanoLuc fusion bands are more prominent.

MLKL antibody has been previously validated using a knockout model for MLKL (reference included in manuscript) and was further validated through inducible induction in knockout cells in our manuscript.

FKBP, from manufacturers website: Detects human, mouse, and rat FKBP12 in Western blots. In Western blots, no cross-reactivity with other FKBP family members is observed.

Caspase-8, from manufacturers website: Positive Western detected in Staurosporine treated Jurkat cells, RAW264.7, HeLa cells, Raji cells, Jurkat cells, UV treated Hela, Sp2/0 cells.

Histone H3, from manufacturers website: Positive Western detected in HeLa, Calf Thymus Histone and NIH/3T3, whole cell lysates. PC12 nuclear lysate. IP: HeLa whole cell extract; IHC-P: FFPE human breast adenocarcinoma.

Cadherin, from manufacturers website: Positive Western detected in extracts from A431, MCF-7, C2C12, HUVEC, BAEC, NIH/3T3, C6, H-4-II-E, Cos and PC3 cells

Eukaryotic cell lines

Policy information about cell lines

Cell line source(s)

293T: ATCC
HT29: ATCC
Immortalized mouse dermal fibroblast (iMDFs): Were isolated from C57BL/6 mouse tails and transformed with SV40 large-T antigen in house

Authentication

293Ts and HT29s were verified through the Garvan Institute of Medical Research using STR profiling. MDFs were isolated in house from C57BL/6 mice, as per established protocols.

Mycoplasma contamination

All cell lines are routinely tested for mycoplasma and the cell lines used in this study were negative for mycoplasma.

Commonly misidentified lines
(See ICLAC register)

There were no commonly misidentified cell lines used in this study.

Animals and other organisms

Policy information about studies involving animals; ARRIVE guidelines recommended for reporting animal research

Laboratory animals

Species: Mouse. Strain: C57BL/6J mice. Animals were selected independently of their gender for generating MDFs, as we find gender does not impact signalling responses in MDFs. Female and male mice were at least 6-weeks old at the time of experimentation. None of the mice used in our experiments had been previously used for other procedures.

Wild animals

The study did not involve wild animals.

Field-collected samples

The study did not involve samples collected from the field.

Ethics oversight

All procedures for this study were approved by the Walter and Eliza Hall Institute (WEHI) Animal Ethics Committee, Australia.

Note that full information on the approval of the study protocol must also be provided in the manuscript.

Flow Cytometry

Plots

Confirm that:

☒ The axis labels state the marker and fluorochrome used (e.g. CD4-FITC).

☒ The axis scales are clearly visible. Include numbers along axes only for bottom left plot of group (a 'group' is an analysis of identical markers).

☒ All plots are contour plots with outliers or pseudocolor plots.

☒ A numerical value for number of cells or percentage (with statistics) is provided.

Methodology

Sample preparation	Cells were seeded into 96-well plates 40 ng/mL doxycycline, to induce construct expression, and treated with 100 ng/mL FLAG-TNF (recombinant human, in-house), 500 nM compound A Smac mimetic (kindly gifted by TetraLogics Pharmaceuticals) and 5 uM IDN-6556 (Cayman) or 10uM Z-VAD-fmk (Selleckchem) overnight (16-24 h). Degrader compounds or a DMSO vehicle control were added for 5 h, as stated in the figure legends. Cells were detached using Trypsin-EDTA (Merck) and resuspended in cell supernatants containing 10 ug/mL propidium iodide (PI)
Instrument	LSR II flow cytometer (Becton Dickinson, NJ)
Software	Flow cytometry data were collected using the BD FACSDiva v9.0 Software.<br>Flow cytometry data were analyzed using WEASEL version 2.7 software (Frank Battye).
Cell population abundance	10,000 single cell events were collected per sample.
Gating strategy	All cells were gated using FSC/SSC. This population was then gated on single cells using FSC-H/FSC-A. PI negative cells were then gated using FSC-A/PI.

☒ Tick this box to confirm that a figure exemplifying the gating strategy is provided in the Supplementary Information.
