# Supplementary material for: Development of NanoLuc-targeting protein degraders and a universal reporter system to benchmark tag-targeted degradation platforms
Source: Nat Commun. 2022 Apr 19;13:2073. doi: 10.1038/s41467-022-29670-1 (PMC9019100; doi:10.1038/s41467-022-29670-1)

S2B

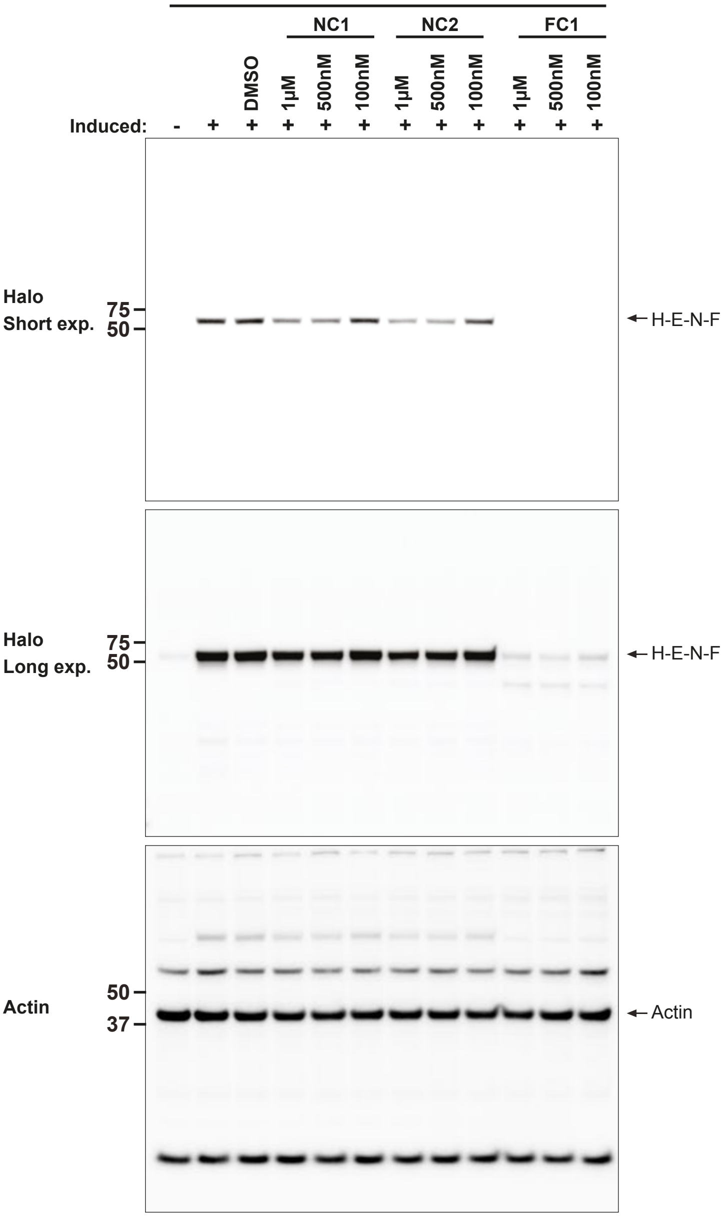
Halo-EGFP-NanoLuc-FKBP^{F36V}
NC1
NC2
FC1
DMSO
1μM
500nM
100nM
1μM
500nM
100nM
1μM
500nM
100nM
Induced: - + + + + + + + + + + +
Halo
Short exp.
75
50
H-E-N-F
Halo
Long exp.
75
50
H-E-N-F
Actin
50
37
Actin

S2D

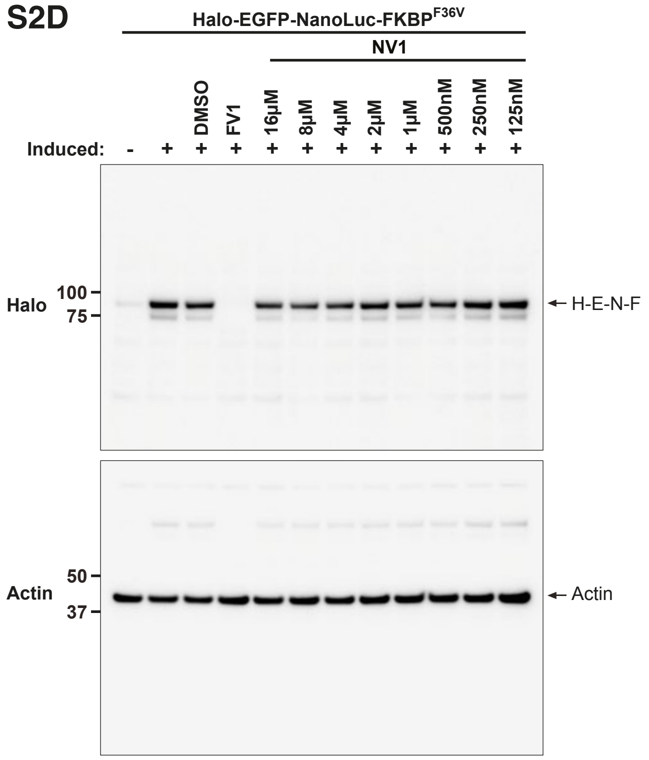
Halo-EGFP-NanoLuc-FKBP^{F36V}
NV1
DMSO
FV1
16μM
8μM
4μM
2μM
1μM
500nM
250nM
125nM
Induced: - + + + + + + + + + + +
Halo
100
75
H-E-N-F
Actin
50
37
Actin

S2E

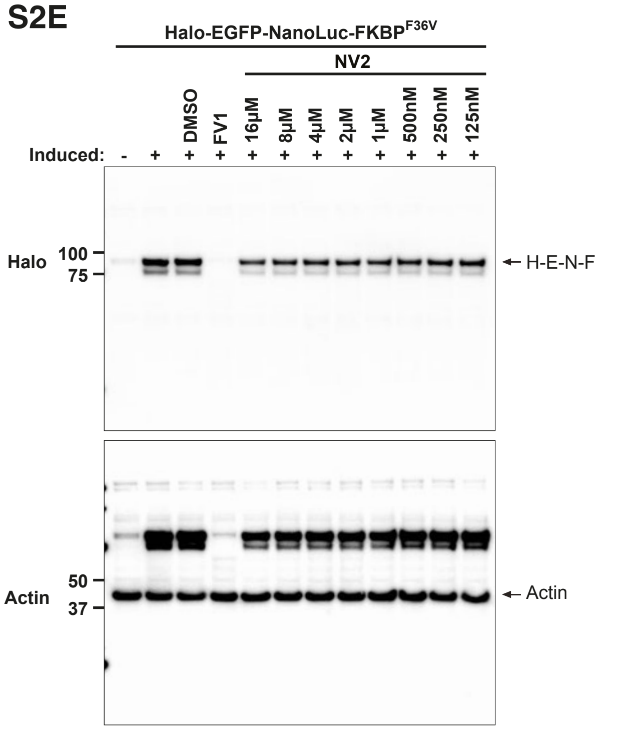
Halo-EGFP-NanoLuc-FKBP^{F36V}
NV2
DMSO
FV1
16μM
8μM
4μM
2μM
1μM
500nM
250nM
125nM
Induced: - + + + + + + + + + + +
Halo
100
75
H-E-N-F
Actin
50
37
Actin

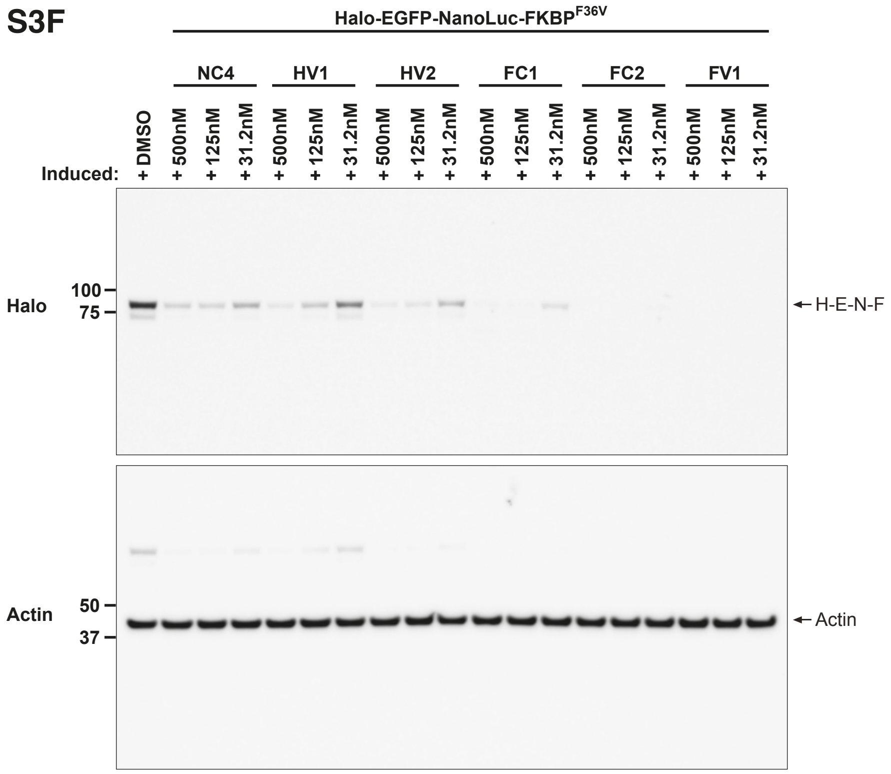
S3F
Halo-EGFP-NanoLuc-FKBPF36V
NC4
HV1
HV2
FC1
FC2
FV1
Induced:
+ DMSO
+ 500nM
+ 125nM
+ 31.2nM
Halo
100
75
H-E-N-F
Actin
50
37
Actin

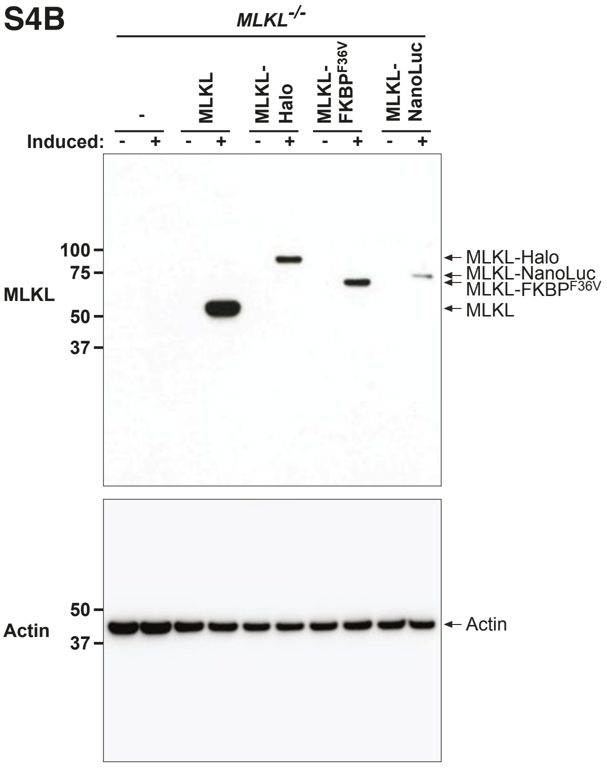
S4B
MLKL-/-
-
MLKL
MLKL-Halo
MLKL-FKBPF36V
MLKL-NanoLuc
Induced:
- +
MLKL
100
75
50
37
MLKL-Halo
MLKL-NanoLuc
MLKL-FKBPF36V
MLKL
Actin
50
37
Actin

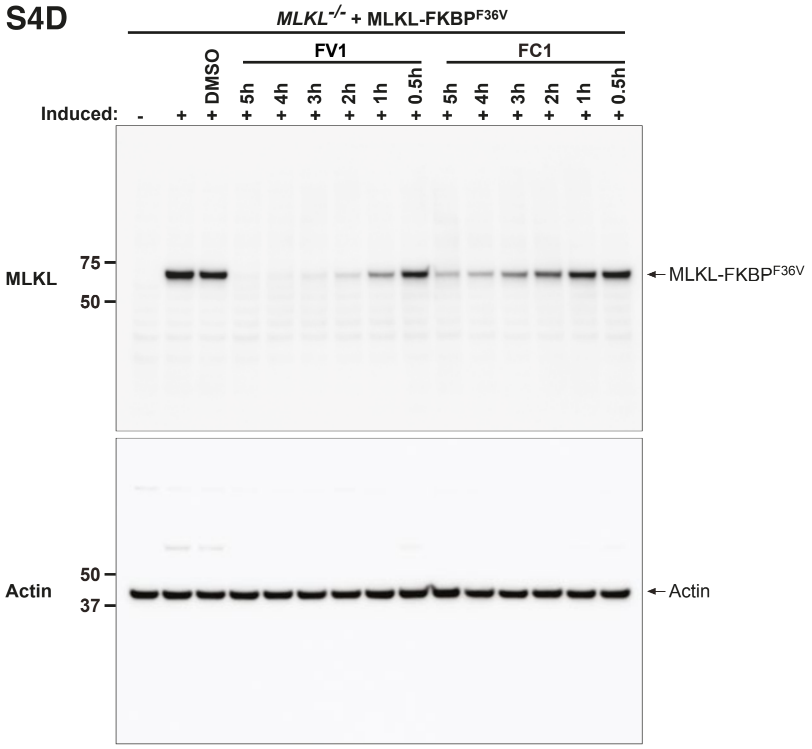
S4D
MLKL-/- + MLKL-FKBPF36V
FV1
FC1
Induced:
-
+
+ DMSO
+ 5h
+ 4h
+ 3h
+ 2h
+ 1h
+ 0.5h
MLKL
75
50
MLKL-FKBPF36V
Actin
50
37
Actin

S4E

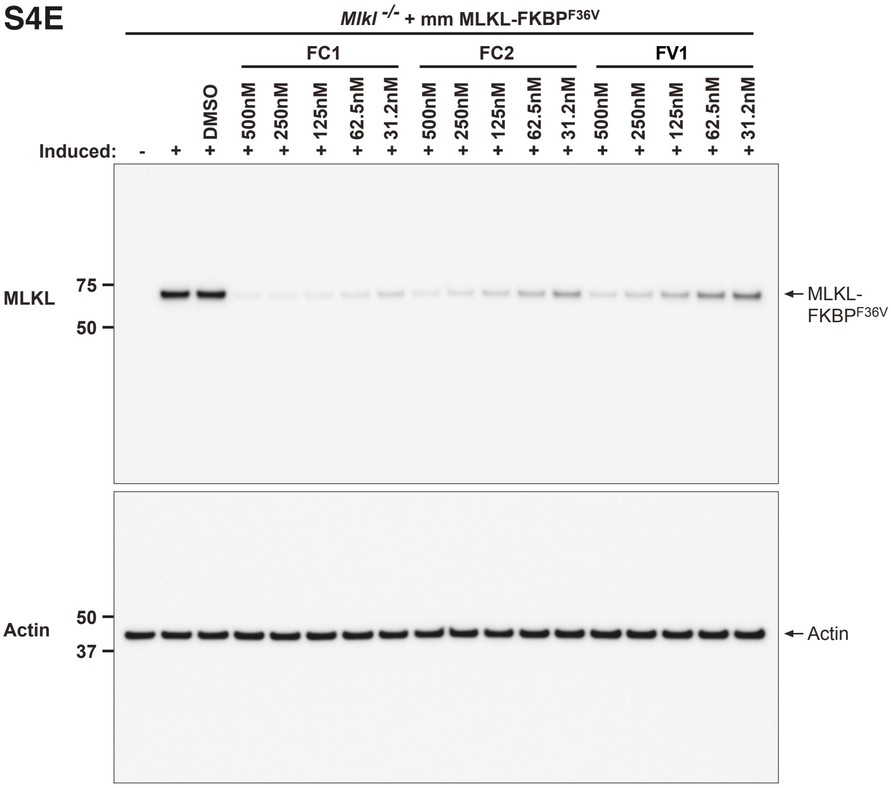

S4I

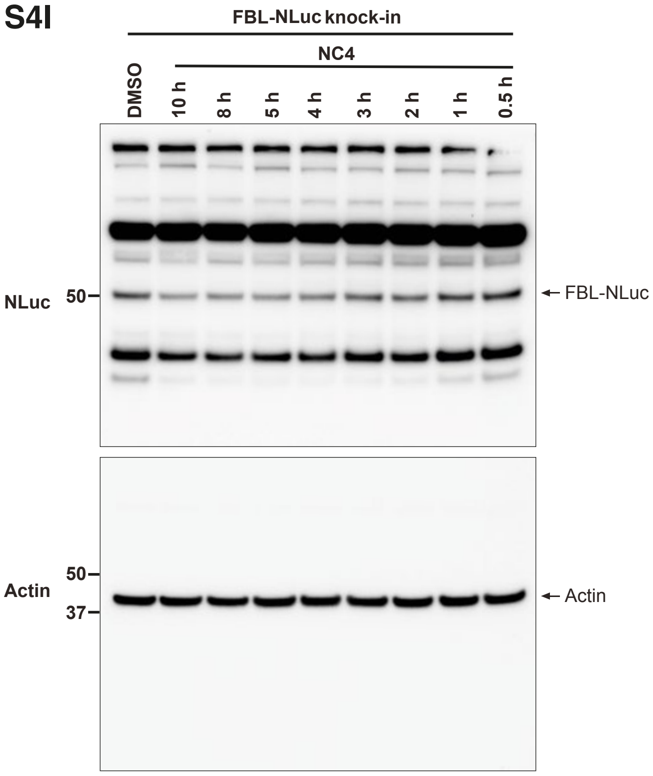

S4F

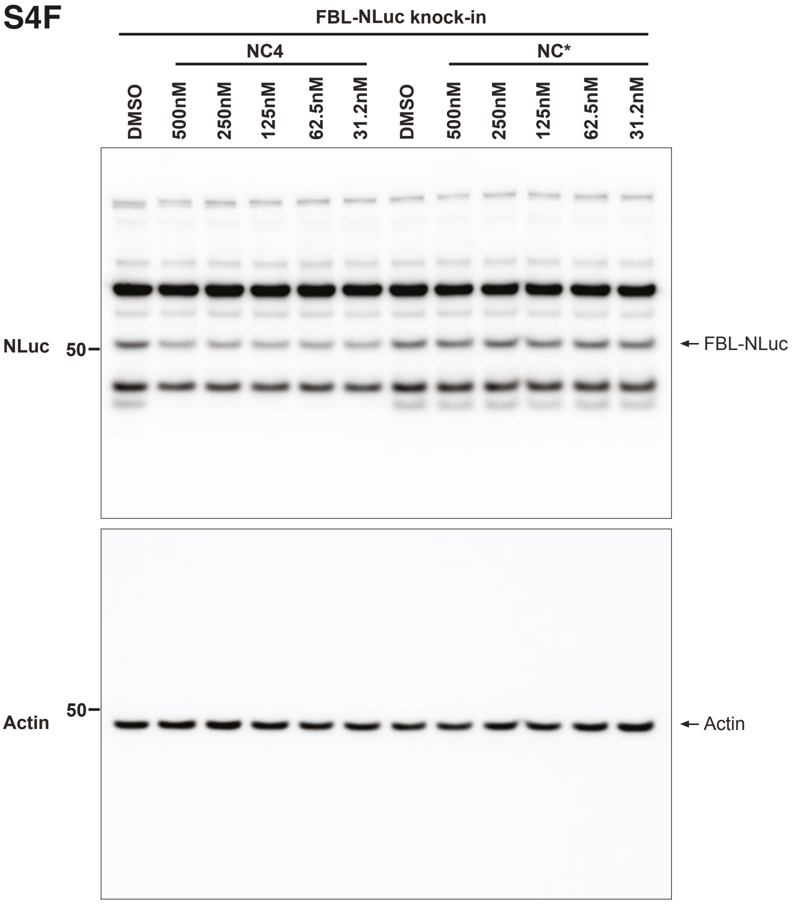

S4J

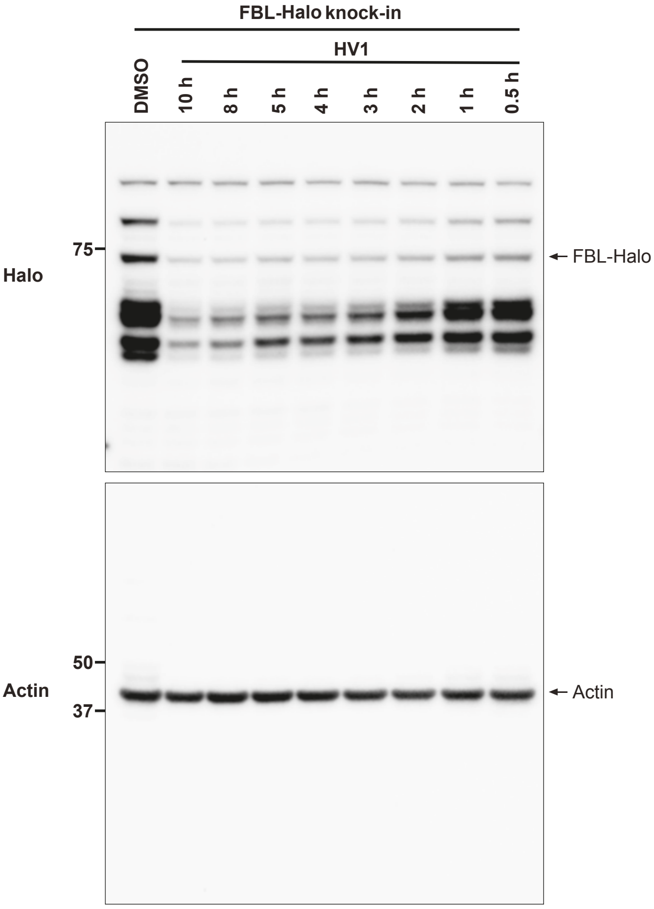

S4G

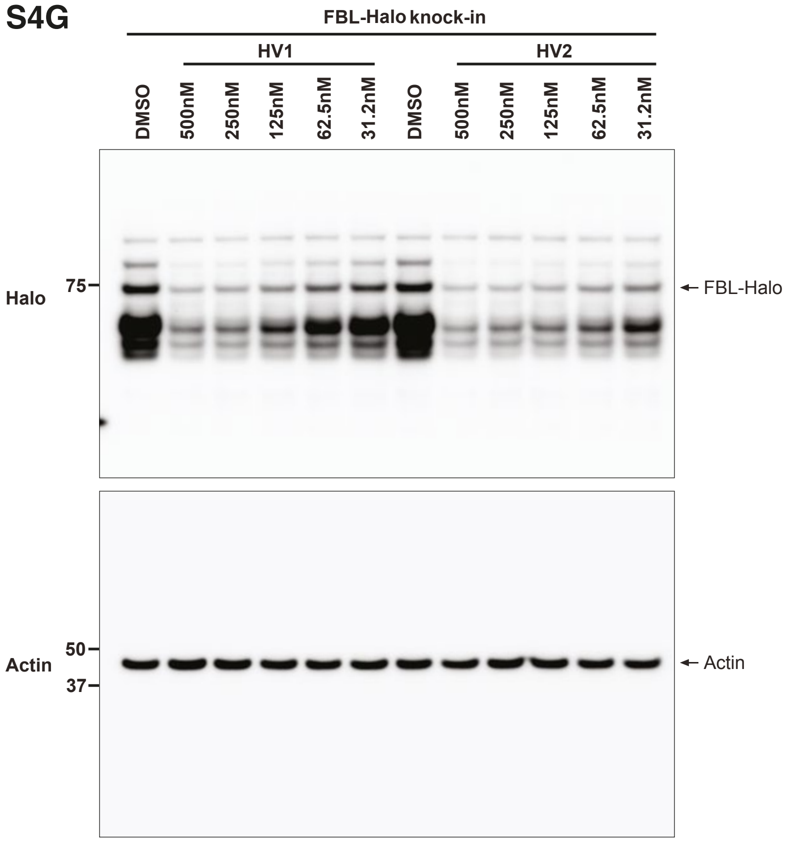

S4H

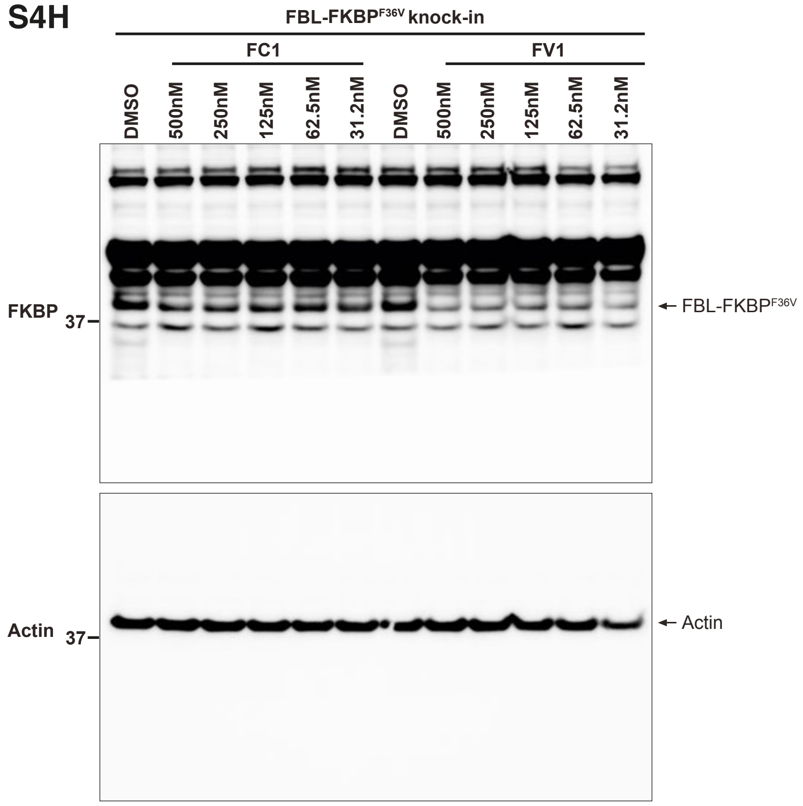

S4K

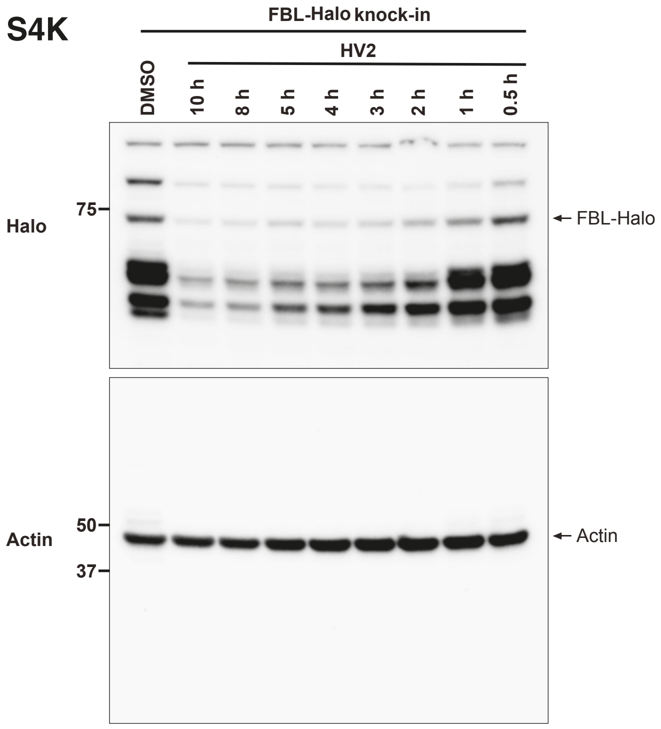

S4L

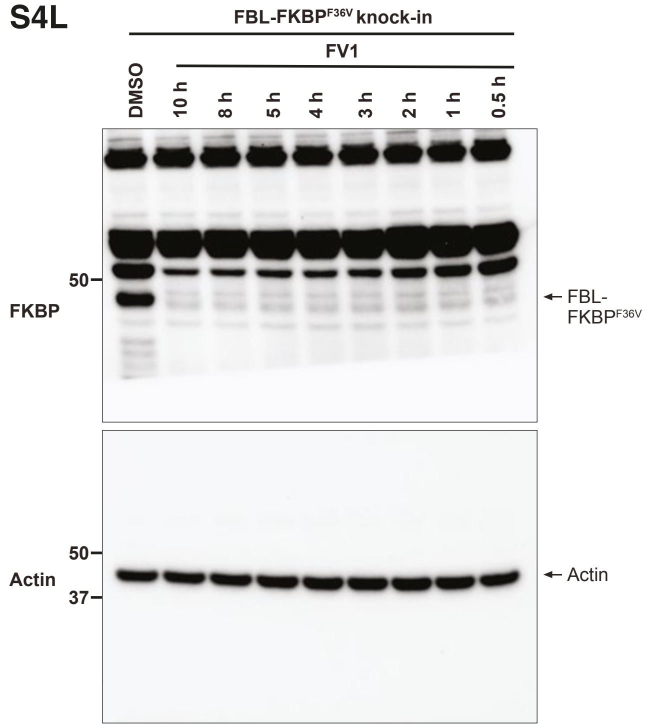

2B

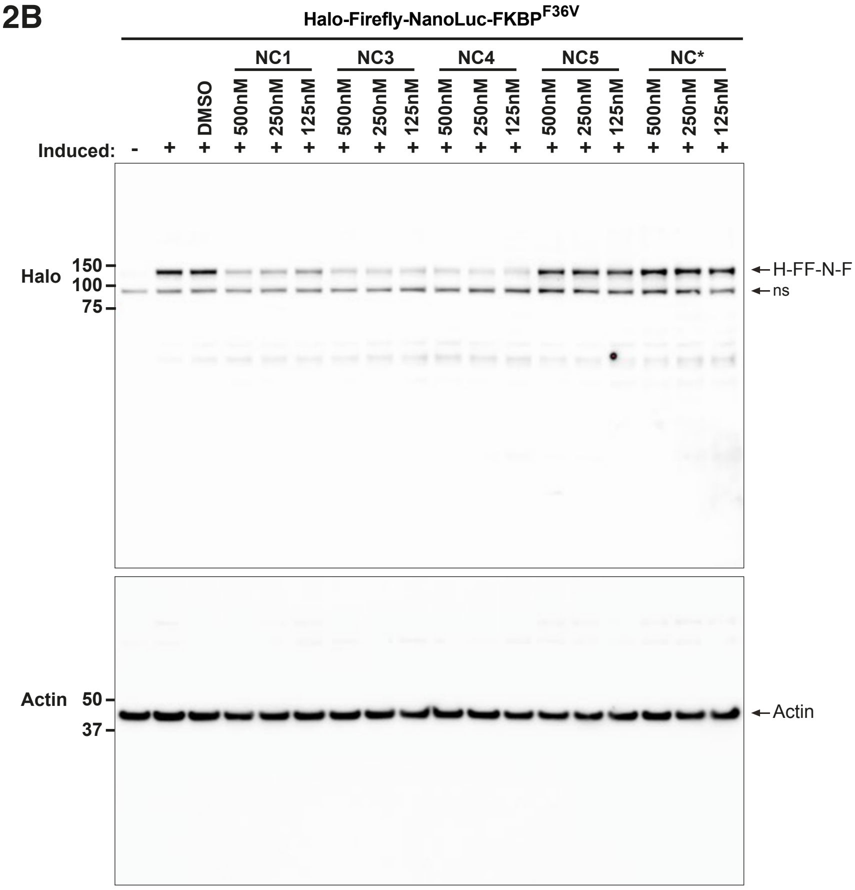
Halo-Firefly-NanoLuc-FKBP^{F36V}
NC1
NC3
NC4
NC5
NC*
Induced: - + + + + + + + + + + + + + + + + +
DMSO
500nM 250nM 125nM 500nM 250nM 125nM 500nM 250nM 125nM 500nM 250nM 125nM 500nM 250nM 125nM
Halo
150
100
75
H-FF-N-F
ns
Actin
50
37
Actin

2E

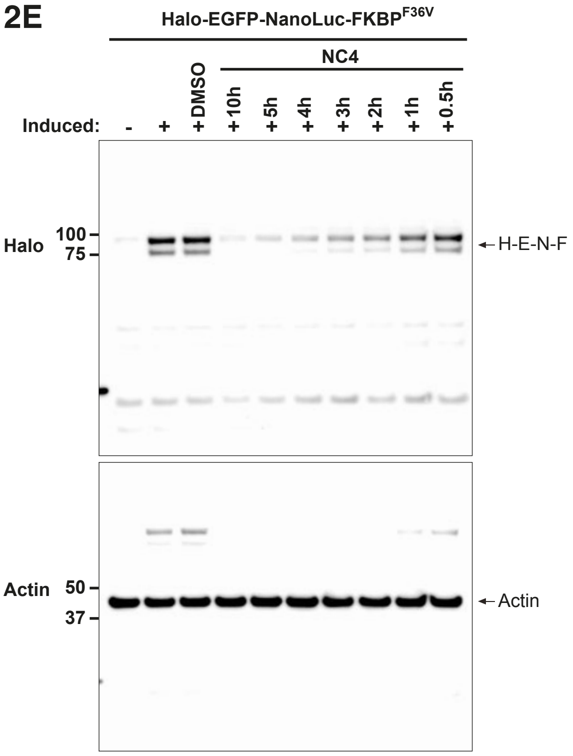
Halo-EGFP-NanoLuc-FKBP^{F36V}
NC4
Induced: - + + + + + + + + +
DMSO
10h 5h 4h 3h 2h 1h 0.5h
Halo
100
75
H-E-N-F
Actin
50
37
Actin

2D

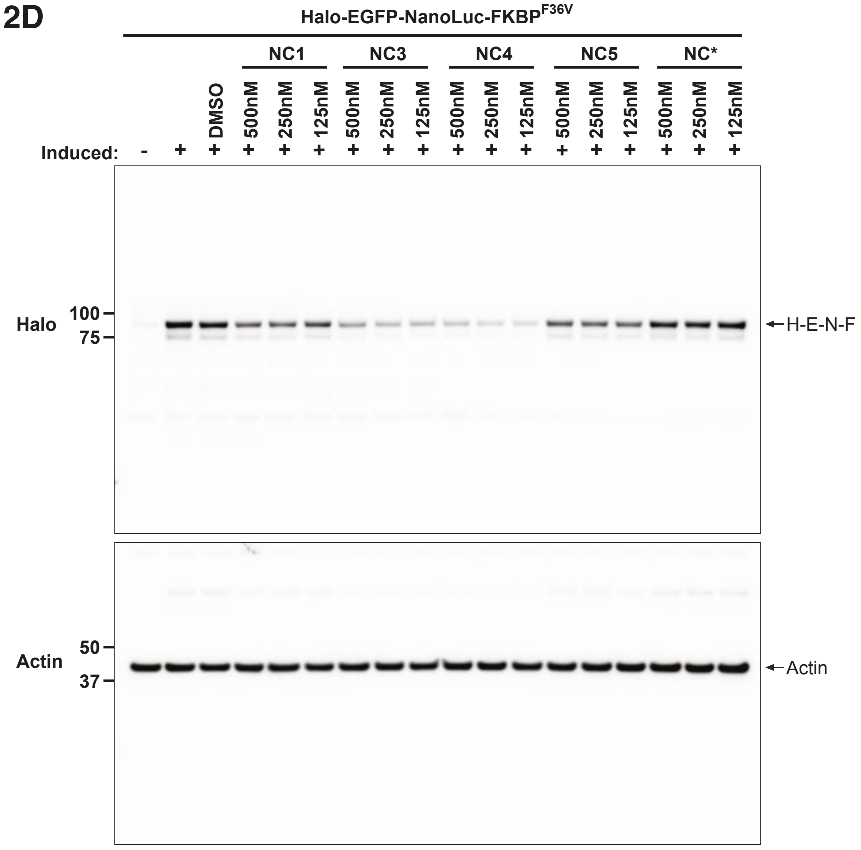
Halo-EGFP-NanoLuc-FKBP^{F36V}
NC1
NC3
NC4
NC5
NC*
Induced: - + + + + + + + + + + + + + + + + +
DMSO
500nM 250nM 125nM 500nM 250nM 125nM 500nM 250nM 125nM 500nM 250nM 125nM 500nM 250nM 125nM
Halo
100
75
H-E-N-F
Actin
50
37
Actin

2G

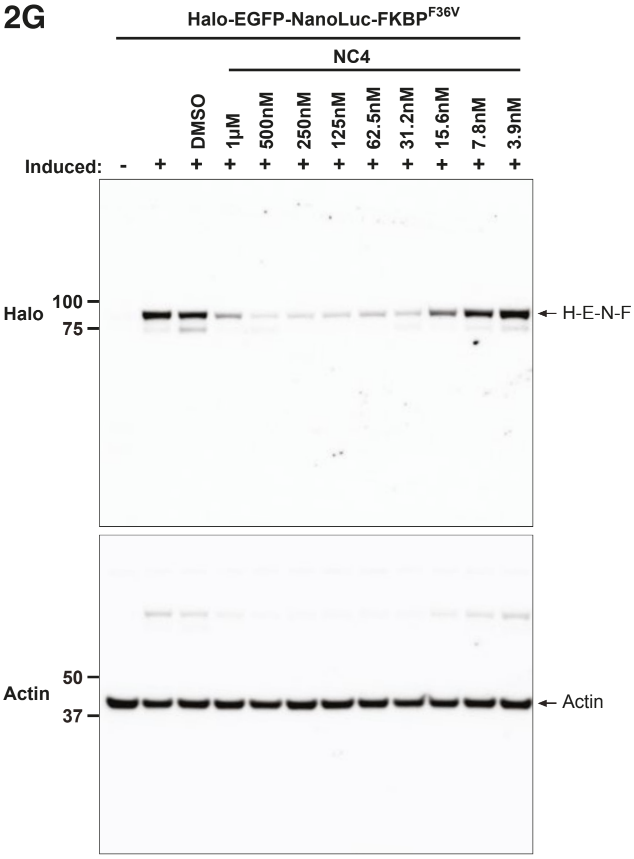
Halo-EGFP-NanoLuc-FKBP^{F36V}
NC4
Induced: - + + + + + + + + + + +
DMSO
1µM 500nM 250nM 125nM 62.5nM 31.2nM 15.6nM 7.8nM 3.9nM
Halo
100
75
H-E-N-F
Actin
50
37
Actin

2F

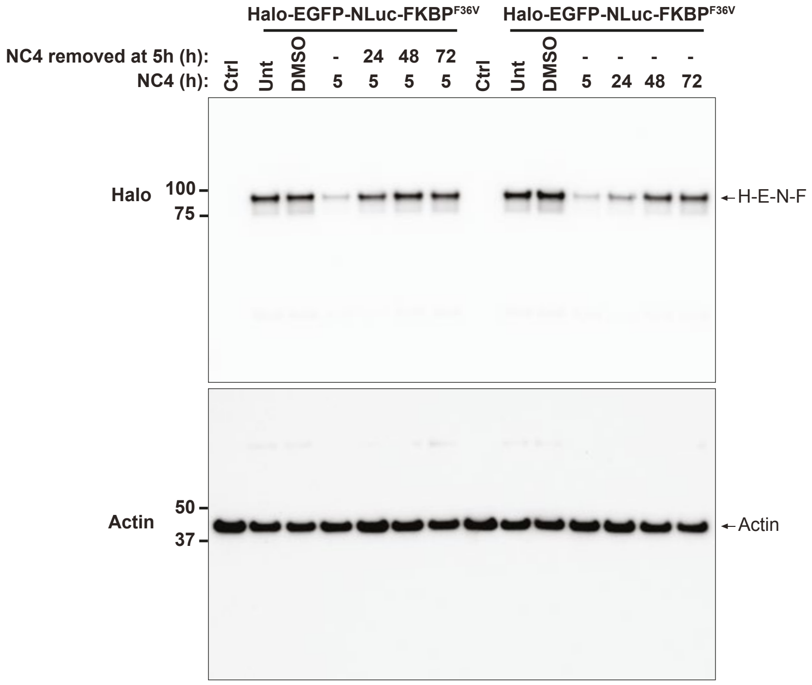
Halo-EGFP-NLuc-FKBP F36V
Halo-EGFP-NLuc-FKBP F36V
NC4 removed at 5h (h):
NC4 (h):
Ctrl
Unt
DMSO
Halo
Actin
100
75
50
37
H-E-N-F
Actin

2J

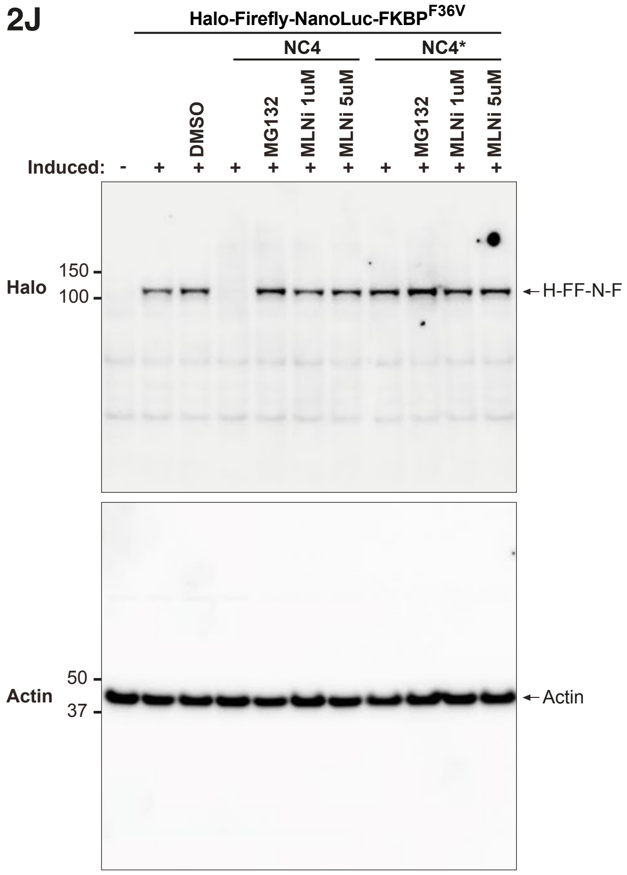
Halo-Firefly-NanoLuc-FKBP F36V
NC4
NC4*
Induced:
DMSO
MG132
MLNi 1uM
MLNi 5uM
Halo
Actin
150
100
50
37
H-FF-N-F
Actin

3D

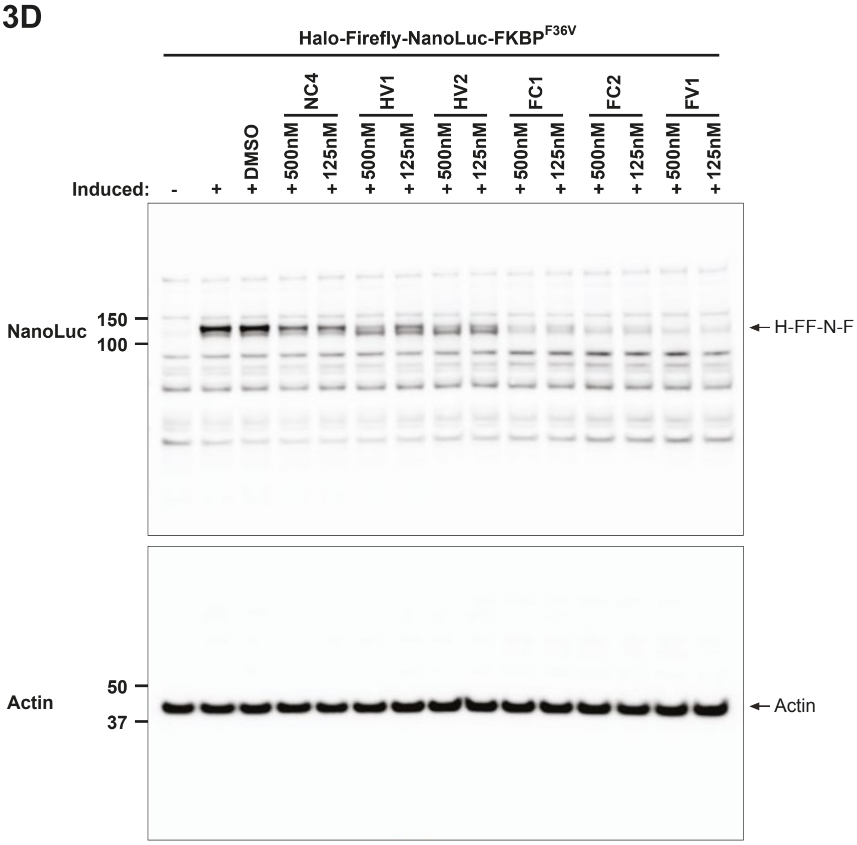
Halo-Firefly-NanoLuc-FKBP F36V
NC4
HV1
HV2
FC1
FC2
FV1
DMSO
500nM
125nM
Induced:
NanoLuc
Actin
150
100
50
37
H-FF-N-F
Actin

4A

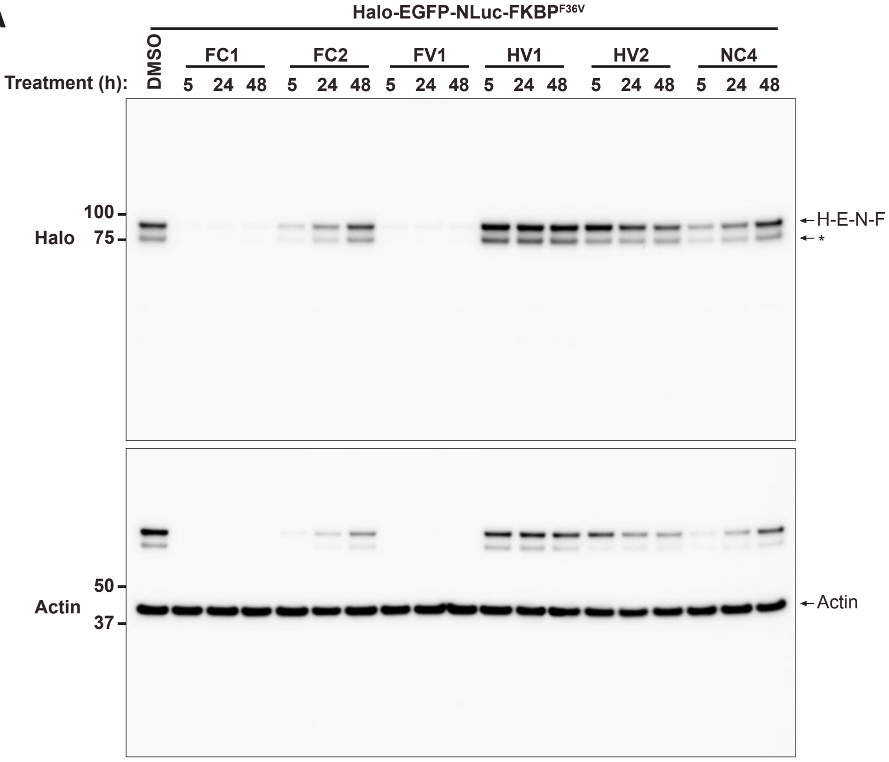

Halo-EGFP-NLuc-FKBP^{F36V}
DMSO
FC1 FC2 FV1 HV1 HV2 NC4
Treatment (h): 5 24 48 5 24 48 5 24 48 5 24 48 5 24 48 5 24 48
100
75
Halo
H-E-N-F
*
50
37
Actin
Actin

4B

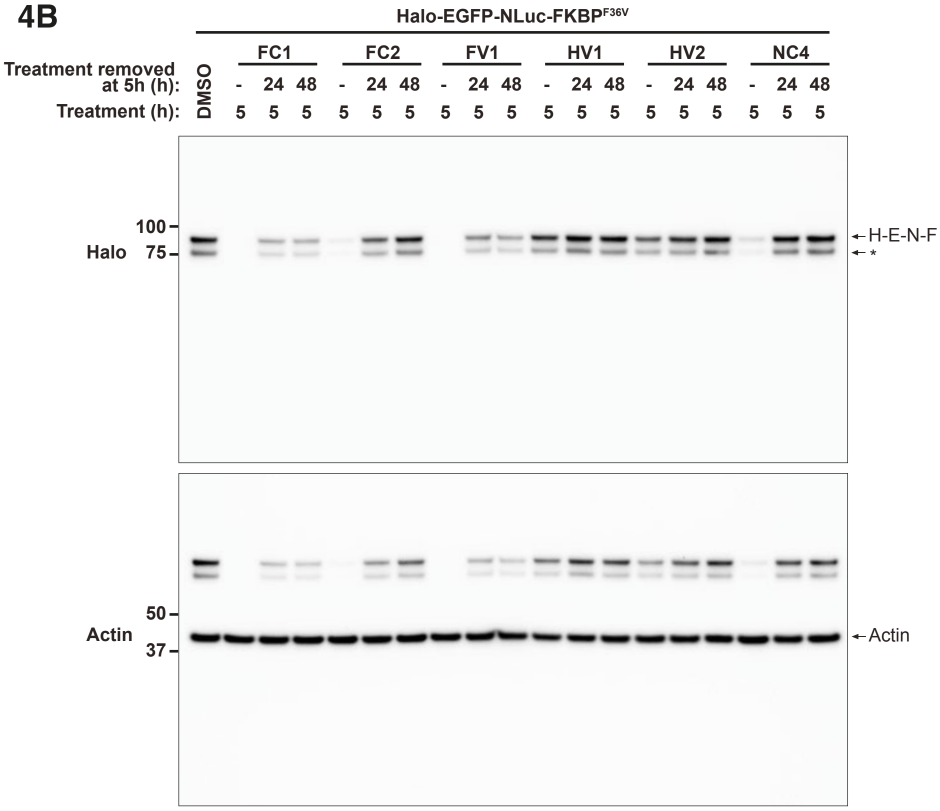

Halo-EGFP-NLuc-FKBP^{F36V}
DMSO
FC1 FC2 FV1 HV1 HV2 NC4
Treatment removed at 5h (h): - 24 48 - 24 48 - 24 48 - 24 48 - 24 48 - 24 48
Treatment (h): 5 5 5 5 5 5 5 5 5 5 5 5 5 5 5 5 5 5
100
75
Halo
H-E-N-F
*
50
37
Actin
Actin

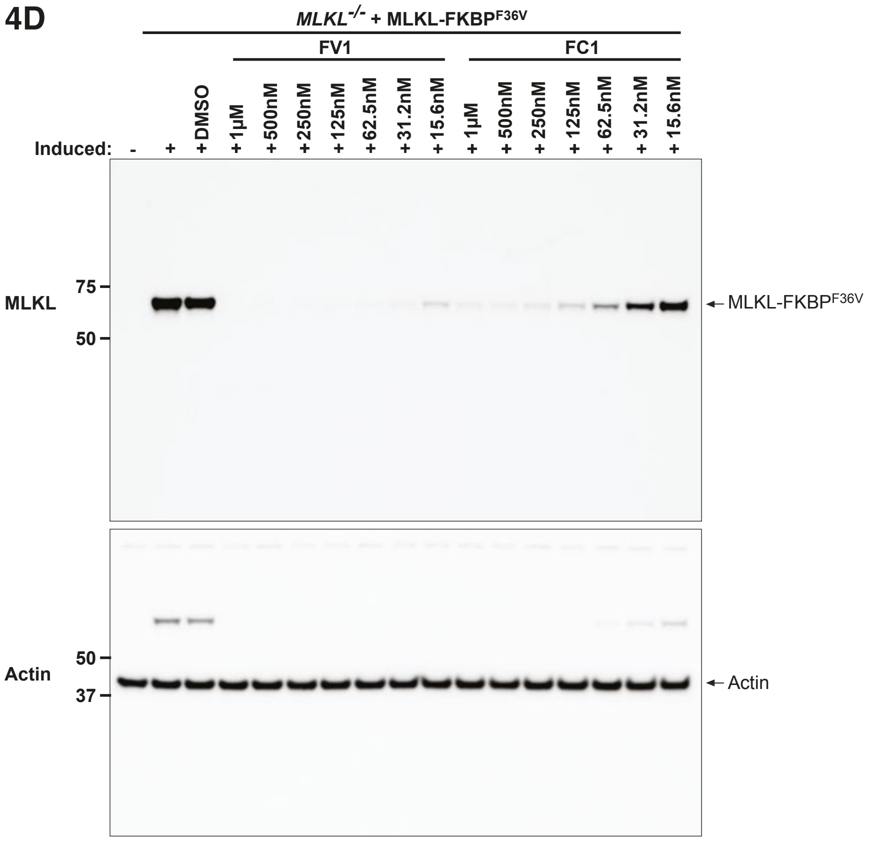
4D
MLKL-/- + MLKL-FKBPF36V
FV1
FC1
Induced:
-
+
+DMSO
+1µM
+500nM
+250nM
+125nM
+62.5nM
+31.2nM
+15.6nM
+1µM
+500nM
+250nM
+125nM
+62.5nM
+31.2nM
+15.6nM
MLKL
75
50
MLKL-FKBPF36V
Actin
50
37
Actin

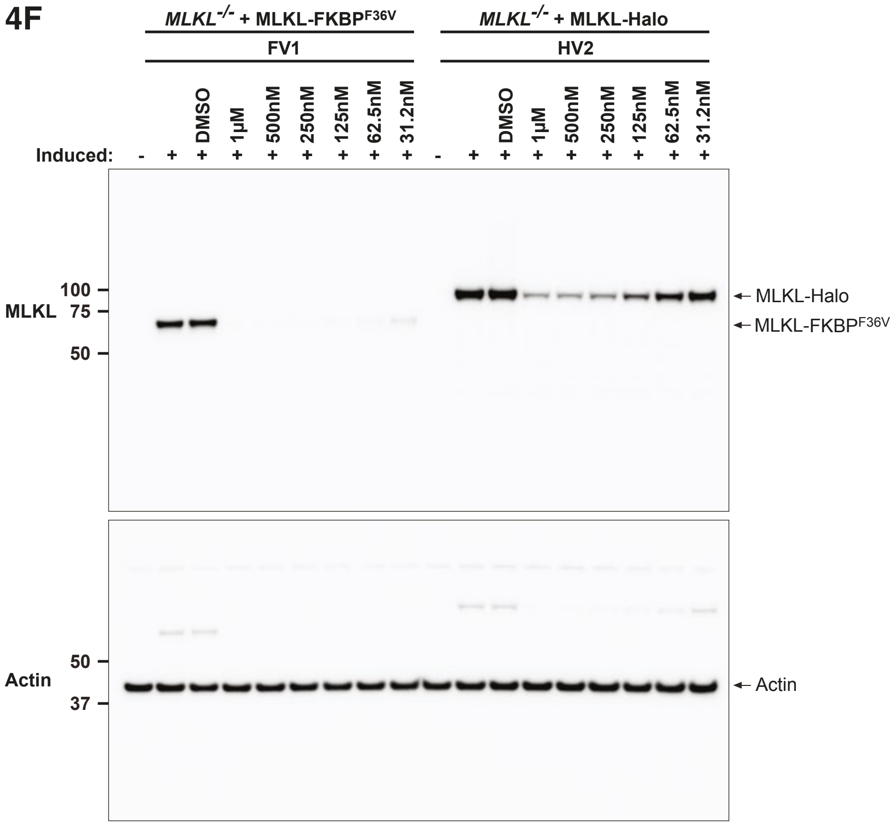
4F
MLKL-/- + MLKL-FKBPF36V
FV1
MLKL-/- + MLKL-Halo
HV2
Induced:
-
+
+ DMSO
+ 1µM
+ 500nM
+ 250nM
+ 125nM
+ 62.5nM
+ 31.2nM
-
+
+ DMSO
+ 1µM
+ 500nM
+ 250nM
+ 125nM
+ 62.5nM
+ 31.2nM
MLKL
100
75
50
MLKL-Halo
MLKL-FKBPF36V
Actin
50
37
Actin

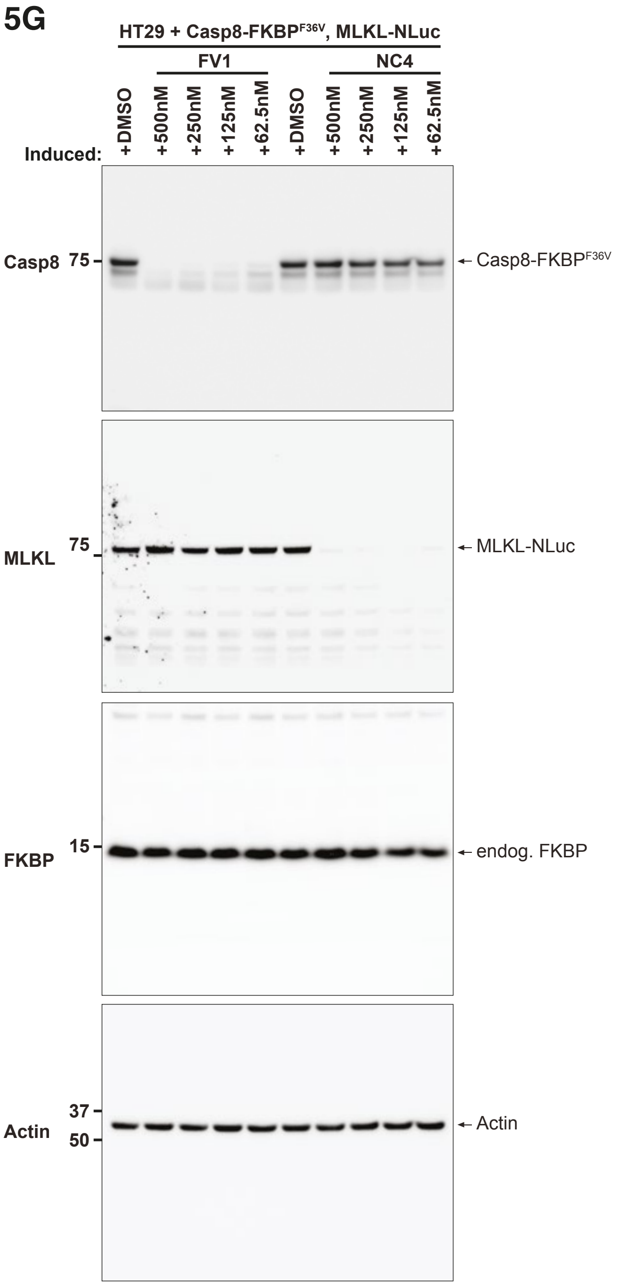
5G
HT29 + Casp8-FKBPF36V, MLKL-NLuc
FV1
NC4
Induced:
+DMSO
+500nM
+250nM
+125nM
+62.5nM
+DMSO
+500nM
+250nM
+125nM
+62.5nM
Casp8
75
Casp8-FKBPF36V
MLKL
75
MLKL-NLuc
FKBP
15
endog. FKBP
Actin
37
50
Actin

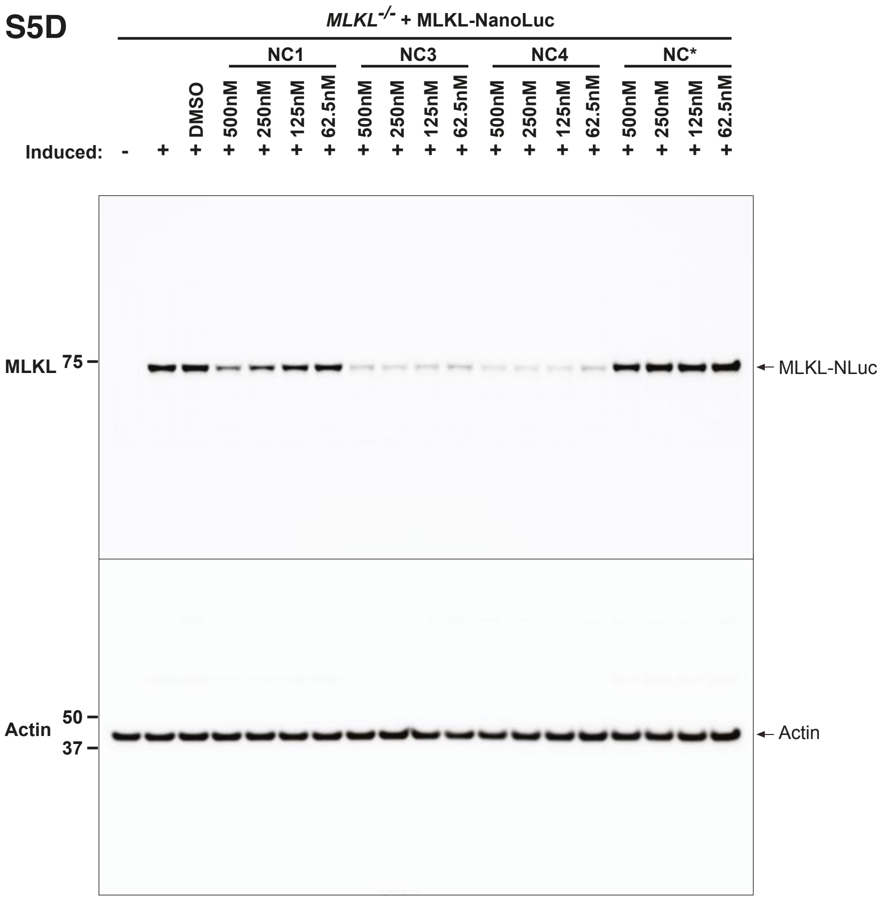
S5D
MLKL-/- + MLKL-NanoLuc
NC1
NC3
NC4
NC*
DMSO
500nM
250nM
125nM
62.5nM
Induced:
MLKL
75
MLKL-NLuc
Actin
50
37
Actin

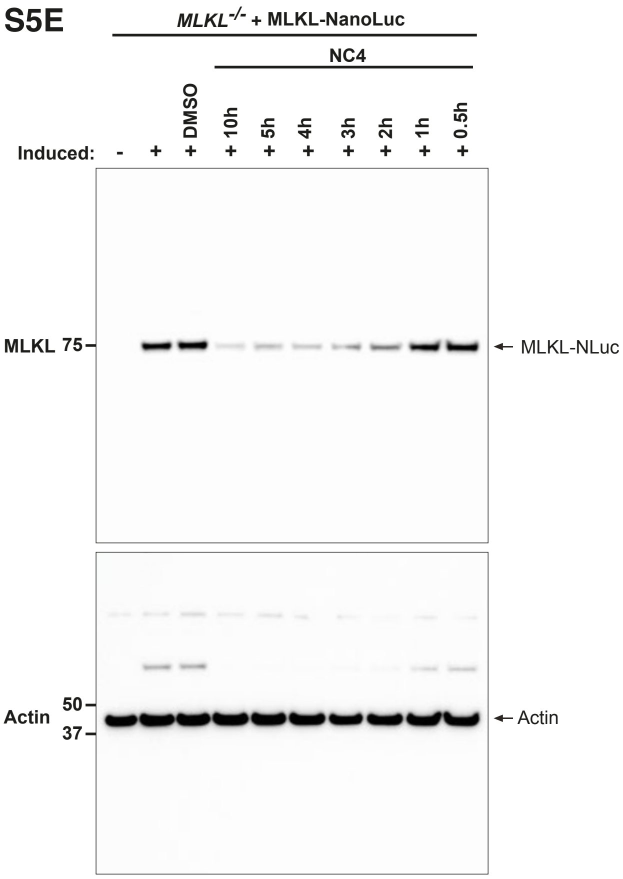
S5E
MLKL-/- + MLKL-NanoLuc
NC4
DMSO
10h
5h
4h
3h
2h
1h
0.5h
Induced:
MLKL
75
MLKL-NLuc
Actin
50
37
Actin

S4M

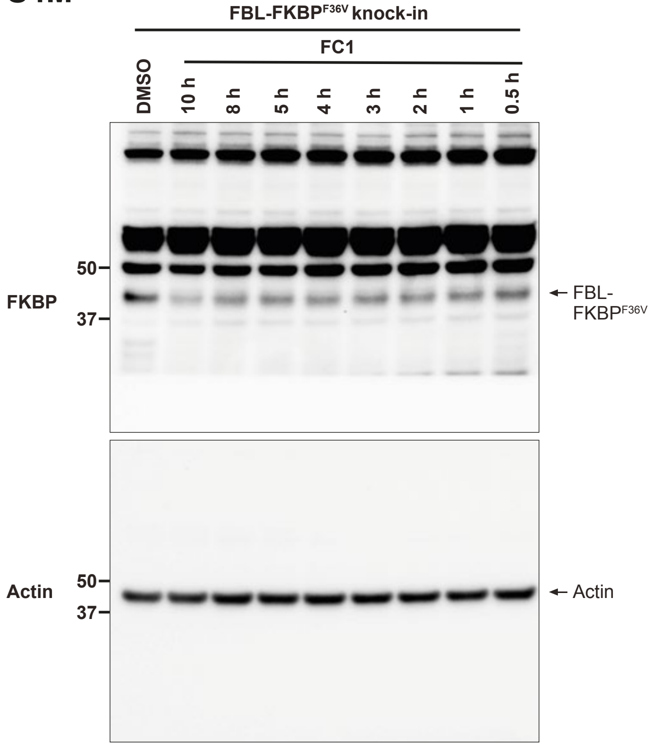

S5A

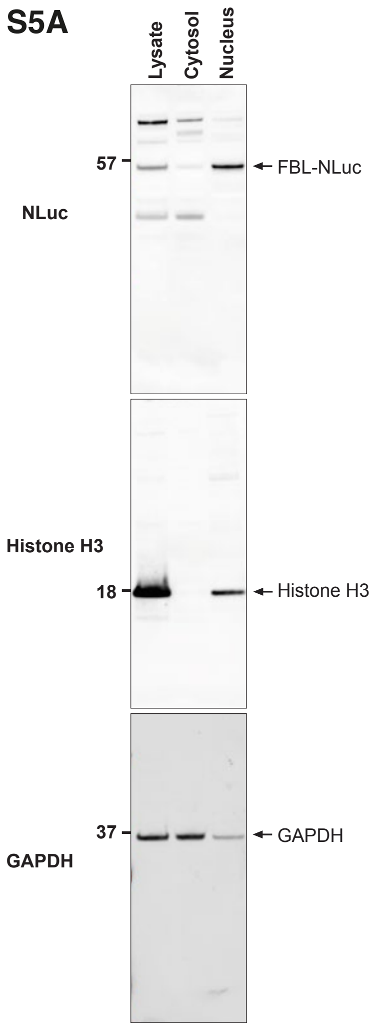

S5B

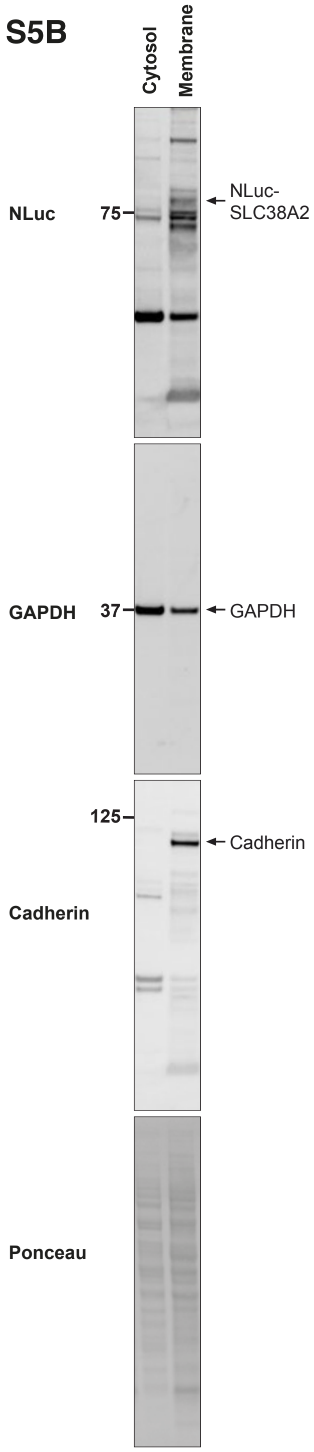

S5C

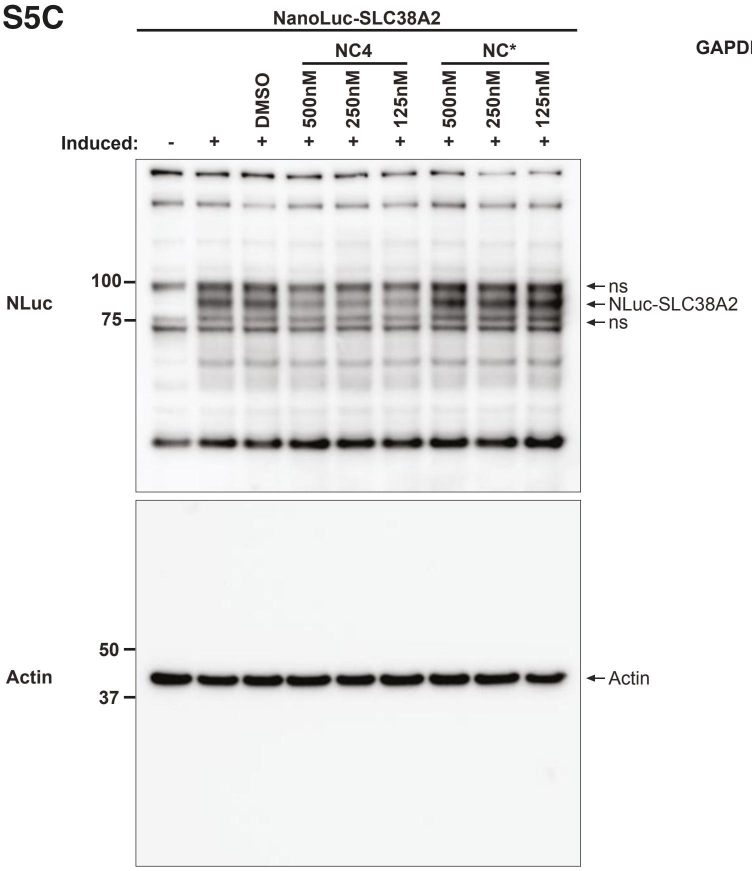

Supplement: Supplementary file 3 — Source Data [file 41467_2022_29670_MOESM3_ESM.zip › Source Data - Raw WBs.pdf]
